# In vivo membrane engineering traps Gd-based MRI contrast agents for detecting microhepatocellular carcinoma

**DOI:** 10.1126/sciadv.aec9913

**Published:** 2026-04-29

**Authors:** Chunping Mao, Jie Yi, Fuan Deng, Wanning Zhu, Jiaru Zhao, Hualing Li, Luoxing Xia, Jianing Ji, Yuxiang Xiong, Jiayao Cai, Lingdong Jiang, Jun Shen, Guobin Hong, Lu Zhang

**Affiliations:** ^1^ Department of Radiology, Zhujiang Hospital, Southern Medical University, Guangzhou 510280, China.; ^2^ Guangdong Provincial Key Laboratory of Advanced Biomaterials, Department of Biomedical Engineering, Southern University of Science and Technology, Shenzhen 518055, China.; ^3^ Department of Gastroenterology, Jingmen Central Hospital, Jingmen 448000, China.; ^4^ Department of Radiology, Sun Yat-Sen Memorial Hospital, Sun Yat-Sen University, Guangzhou 510120, China.

## Abstract

Accurate detection of microhepatocellular carcinoma (HCC) remains a major clinical challenge owing to the limited specificity and sensitivity of current imaging modalities. Herein, we present a dual-injection magnetic resonance imaging (MRI) peptidic probe based on in vivo membrane engineering, achieving in situ signal amplification and molecularly precise imaging. The first injection of programmable nanoparticles coassembled from two peptide monomers, incorporating a GPC3-targeting ligand, a β sheet–forming motif, a dibenzocyclooctyne (DBCO) handle, and porphyrin IX (PpIX) for fluorescence tracking. Following systemic administration, the nanoparticles high-specifically bind to GPC3-overexpressing tumor membranes and transform into surface-anchored nanofibrils, exposing confined DBCO groups. A second injection of azide-modified Gd-DOTA enables rapid copper-free click conjugation on the nanofibrillar scaffold, yielding a nearly fourfold increase in longitudinal relaxivity. MRI demonstrated strong *T*_1_-weighted signal enhancement and high tumor-to-liver contrast in Hepa1-6 tumor-bearing mice. In vivo membrane engineering strategy establishes a generalizable platform for receptor-guided molecular imaging and early cancer detection.

## Teaser

In vivo self-assembling peptides engineer tumor membranes to trap Gd contrast agents for sensitive MRI of micro-HCC.

## INTRODUCTION

Hepatocellular carcinoma (HCC) represents the dominant histological subtype of primary liver cancer, accounting for 75 to 90% of all liver cancer cases (*1*–*3*). Despite therapeutic advances, including surgical resection, liver transplantation, percutaneous ablation, transarterial chemoembolization (TACE) or transarterial radioembolization (TARE), as well as molecular targeted therapies and immunotherapy (*4*), the 5-year survival rate for unresectable intermediate-to-advanced HCC remains below 15% (*5*). In contrast, early detection and intervention in micro-HCC (<1 cm in diameter) can achieve a 5-year survival rate exceeding 98.5%, approaching curative outcomes (*6*, *7*). However, the accurate identification of micro-liver lesions remains a substantial challenge in clinical practice. Currently available diagnostic modalities for detecting micro-HCC exhibit substantial limitations (*8*). First, serum biomarkers such as alpha-fetoprotein (AFP), commonly quantified using chemiluminescence immunoassay (CLIA), exhibit poor sensitivity (<20%) for the detection of early-stage HCC (*9*). Second, although histopathological examination remains the diagnostic gold standard, preoperative needle biopsy is often unreliable for small nodules, thereby limiting its diagnostic value in early-stage HCC. Third, imaging modalities present critical limitations: Positron emission tomography/computed tomography (PET/CT) involves substantial radiation exposure and offers limited sensitivity (*10*), whereas conventional ultrasonography is hindered by inadequate spatial resolution and operator-dependent variability, reducing its utility for detecting small lesion (*11*).

Magnetic resonance imaging (MRI) is a primary modality for diagnosing and staging HCC (*10*, *12*). Clinically approved gadolinium-based contrast agents (GBCAs), classified as extracellular fluid agents, are routinely administered in dynamic contrast–enhanced MRI (DCE-MRI) to enhance *T*_1_-weighted contrast by shortening the longitudinal relaxation time (*T*_1_) of water protons (*13*, *14*). However, conventional GBCAs lack molecular specificity, limiting their ability to characterize lesions accurately. Even hepatobiliary-specific agents, such as gadoxetate disodium (Eovist or Primovist), demonstrate suboptimal sensitivity (<53%) for detecting liver nodules 1 to 2 cm in diameter and fail to reliably identify subcentimeter lesions (*15*). Moreover, their inherently low longitudinal relaxivity (*r*_1_), together with the poor arterial perfusion characteristic of early-stage HCC, diminishes the detection performance of DCE-MRI (*16*). To date, no clinically approved GBCAs enable robust and reliable detection of micro-HCC, underscoring a critical unmet need for next-generation contrast agents with enhanced sensitivity and molecular specificity.

Nanoscale MRI molecular imaging probes have attracted increasing interest as next-generation contrast agents for detecting early-stage HCC. These probes are engineered to overcome the inherent limitations of clinically approved GBCAs, including low molecular specificity, limited *r*_1_ relaxivity, and poor sensitivity. Multiple strategies have been explored to enhance early-stage HCC detection. For example, an aptamer-conjugated ultrasmall superparamagnetic iron oxide probe (Apt-USPIO) was developed for HCC-specific targeting (*17*). A Gd-based self-assembling nanoprobe (DOTA-Gd-CBT-NP) responsive to γ-glutamyl transpeptidase (GGT) overexpression markedly increased local *r*_1_ relaxivity through in situ nanofiber formation (*18*). A MnFe_2_O_4_-EOB-PEG nanocontrast agent targeting organic anion-transporting polypeptides (OATPs) on normal hepatocytes enabled negative contrast of micro-HCC via multiphase imaging (*15*). Although these pioneering efforts represent meaningful advances in targeted MRI contrast agent development, most fail to deliver both high specificity for micro-HCC biomarkers and substantial relaxivity enhancement. Consequently, a critical unmet clinical need persists for molecular imaging probes that combine robust *r*_1_ relaxivity with high tumor specificity—particularly those capable of generating positive contrast for the reliable detection of micro-HCC.

In vivo self-assembling peptides offer a versatile platform for constructing such next-generation molecular imaging probes. Typically incorporating β sheet–forming motifs such as VVVVV or FF (*19*), these peptides spontaneously assemble into stable nanofibrous architectures through noncovalent interactions, including hydrogen bonding and π-π stacking. Self-assembling peptide drugs (SPDs) are particularly attractive for imaging owing to their modular design, low systemic toxicity, and high biological specificity (*20*). SPDs are generally engineered with three functional domains: a hydrophilic targeting ligand, a hydrophobic β sheet motif, and a functional moiety such as an imaging or therapeutic agent. Upon engagement with overexpressed membrane receptors, initial SPD nanoparticles undergo conformational rearrangement into extended fibrillar structures that stably anchor to the cell membrane (*21*, *22*). Notably, incorporation of GBCAs into these fibrillar networks notably increases the rotational correlation time (τ_R_) of the Gd chelates—a critical determinant of *r*_1_ relaxivity (*23*). When coupled with favorable water coordination dynamics (e.g., hydration number and residence time), this immobilization strategy markedly enhances local MRI signal intensity (*24*). We previously demonstrated that SPD-based systems targeting membrane receptors such as human epidermal growth factor receptor 2 (HER2), programmed cell death ligand 1 (PD-L1), and integrin α_3_β_1_ can form extensive fibrillar networks on the tumor surface, yielding enhanced fluorescence imaging and immunomodulatory effects (*25*–*27*). Nevertheless, it remains unclear how SPD platforms can be strategically engineered into tumor-specific, high-relaxivity MRI probes for the reliable detection of micro-HCC.

Enhancing probe specificity fundamentally depends on identifying of highly selective molecular targets. Glypican-3 (GPC3), a membrane-bound proteoglycan, is overexpressed in ~90% of HCC cases—including microlesions—while being virtually absent in normal hepatocytes (*28*). Owing to its pivotal role in tumor growth and metastasis, GPC3 represents an attractive target for both imaging and therapy for HCC. Conjugating GBCAs with self-assembling peptides bearing GPC3-binding motifs, such as DHLASLWWGTEL (*29*), provides a rational strategy to selectively label micro-HCC with high sensitivity. In the latest molecular imaging studies of HCC, GPC3 has emerged as a key targeting moiety (*30*–*32*). Despite this promise, the clinical translation of tumor-targeted MRI probes is frequently hindered by issues including complexity, suboptimal biocompatibility, and inefficient in vivo labeling. To address these limitations, we use bioorthogonal chemistry—specifically, copper-free click reactions, such as strain-promoted azide-alkyne cycloaddition (SPAAC)—to enable high-efficiency, site-specific probe conjugation in living systems (*33*–*35*). This catalyst-free reaction proceeds rapidly under physiological conditions and minimizes off-target effects, rendering it particularly suitable for two-step in vivo targeting systems (*35*–*38*).

**Fig. 1.**
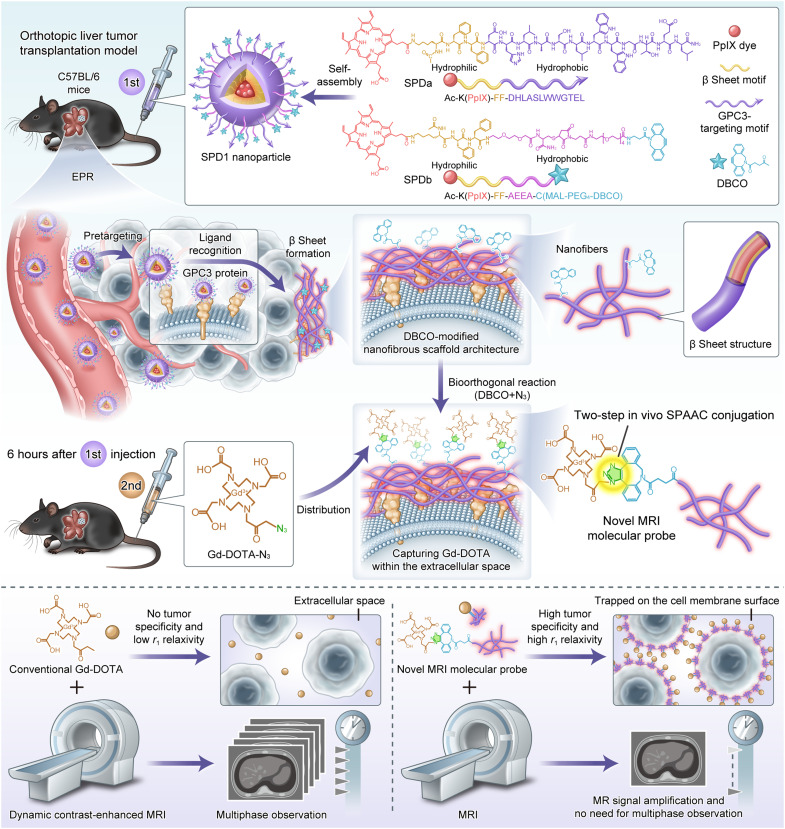
Schematic illustration of an MRI molecular probe based on in vivo membrane engineering and bioorthogonal click chemistry for micro-HCC detection. SPD1 comprises two peptides [SPDa, Ac-K(PpIX)-FF-DHLASLWWGTEL; SPDb, Ac-K(PpIX)-FF-AEEA-C(MAL-PEG_4_-DBCO)], which are composed of four discrete functional domains: (i) DHLASLWWGTEL, a GPC3-targeted motif; (ii) FF, a β sheet motif; (iii) PpIX, a fluorescent dye; (iv) DBCO, a click chemistry group. SPD1 self-assembles into nanoparticles, and SPD1 and Gd-DOTA-N_3_ are administered via two sequential injections. SPD1 nanoparticles (first intravenous administration) first accumulate and transform into fibrillar networks on the cell membrane of high GPC3-expressing orthotopic liver tumor through intermolecular π-π stacking between FF. The fibrillar networks, functionalized with DBCO, trapped Gd-DOTA-N_3_ (following a second intravenous administration) in the extracellular space via a bioorthogonal reaction and immobilized it in an ordered arrangement, thereby enhancing the *T*_1_-weighted signal for the precise detection of micro-HCC. Schematic elements were created by eBioart.

To address the unmet need for accurate MRI detection of micro-HCC, we propose a sequential in vivo membrane engineering strategy that integrates the tumor specificity of SPDs with the chemical precision of bioorthogonal click chemistry (Fig. 1). We designed two peptide monomers [SPDa, Ac-K(PpIX)-FF-DHLASLWWGTEL; SPDb, Ac-K(PpIX)-FF-AEEA-C(MAL-PEG_4_-DBCO)] that coassemble into SPD1 nanoparticles, which are composed of (i) a GPC3-targeting peptide (DHLASLWWGTEL) for selective recognition of HCC, (ii) a β sheet–forming motif (FF) for fibrillar transformation, (iii) a dibenzocyclooctyne (DBCO) handle enabling copper-free click chemistry, and (iv) a protoporphyrin IX (PpIX) core for fluorescence guidance. Following intravenous administration, SPD1 preferentially accumulates in tumor tissue via the enhanced permeability and retention (EPR) effect. Upon engagement with membrane-expressed GPC3, SPD1 undergoes fibrillar transformation and displays DBCO handles on the tumor surface. A second systemic administration of azide-modified GBCAs (Gd-DOTA-N_3_) subsequently reacts with the anchored DBCO via SPAAC, generating MRI-active nanostructures precisely at the tumor site. This in situ assembly mechanism enhances local *r*_1_ relaxivity, yielding high-contrast MRI signals capable of revealing micro-HCC lesions. By integrating programmable peptide self-assembly with bioorthogonal signal amplification, our strategy provides a tumor-specific, clinically translatable platform for early HCC detection.

## RESULTS

### Synthesis and characterization of SPDs and Gd-DOTA-N_3_

SPDa [Ac-K(PpIX)-FF-DHLASLWWGTEL] and SPDb [Ac-K(PpIX)-FF-AEEA-C(MAL-PEG_4_-DBCO)] were synthesized via the standard solid-phase peptide synthesis (SPPS) method as previously described (*39*, *40*). During the synthesis of SPDb, a thiol-containing linker (AEEA-C) conjugated with FF and PpIX was further reacted with DBCO-PEG_4_-MAL via an addition reaction. The chemical structures of SPDa and SPDb are shown in fig. S1, and the detailed synthesis routes were provided by Taikubio (Hefei, China) (figs. S2 and S3). The molecular weights of SPDa and SPDb were respectively confirmed by matrix-assisted laser desorption/ionization–time-of-flight mass spectrometry (MALDI-TOF MS) (fig. S4) and electrospray ionization mass spectrometry (ESI-MS) (fig. S5), consistent with their theoretical values (fig. S1). The key intermediates involved in the synthesis routes of SPDa and SPDb were further confirmed by ESI-MS (fig. S6). The purities of SPDa and SPDb, determined by high-performance liquid chromatography (HPLC), exceed 95%, as shown in fig. S7. The proton nuclear magnetic resonance (^1^H NMR) spectra confirm the chemical structures of SPDa and SPDb (fig. S8). In addition, Gd-DOTA-N_3_, an azide-modified MRI contrast agent, was purchased from a commercial source (Ruixibio, R-315-208).

Grand average of hydropathy (GRAVY) analysis shows that the GPC3-targeting motif (DHLASLWWGTEL; GRAVY ≈ −0.06) is hydrophilic, and the AEEA and PEG_4_ linkers further enhance overall hydrophilicity, whereas the β sheet–forming motif (FF) displays pronounced hydrophobicity (GRAVY = 2.8). Together with the distinctly hydrophobic PpIX moiety (*41*), the incorporation of these five segments within a single peptide confers a well-defined amphiphilic character, thereby favoring spontaneous self-assembly in aqueous solution.

**Fig. 2.**
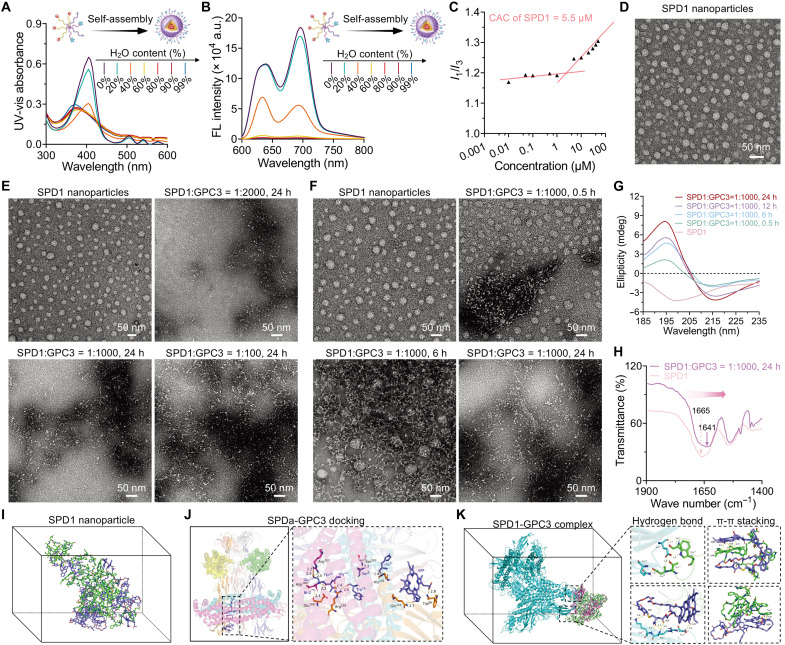
Self-assembly and GPC3-induced fibrillar transformation of SPD1. (**A** and **B**) UV-vis absorption spectra (A) and fluorescence (FL) emission spectra (B) of PpIX (excitation: 405 nm) following the gradual addition of H_2_O (from 0 to 99.5%) to a DMSO solution of SPD1 nanoparticles. a.u., arbitrary units. (**C**) CAC of SPD1 nanoparticles was determined using pyrene as a fluorescent probe. (**D**) Representative TEM image of self-assembled SPD1 nanoparticles (50 μM) in aqueous solution. (**E**) TEM images showing the initial SPD1 nanoparticles and nanofibers transformed from SPD1 nanoparticles (50 μM) after incubation with human GPC3 protein [molecular weight (MW) ≈ 61.6 kDa] at varying molar ratios. h, hours. (**F**) TEM images showing the initial SPD1 nanoparticles and nanofibers transformed from SPD1 nanoparticles (50 μM) after incubation with human GPC3 protein at different time points. The molar ratio of human GPC3 protein/SPD1 was ~1:1000. (**G**) CD spectra of SPD1 nanoparticles (50 μM) before and after incubation with human GPC3 protein (1:1000 molar ratio) for various durations, showing the secondary structure transition. mdeg, millidegrees. (**H**) FTIR spectra of SPD1 nanoparticles (50 μM) before and after 24 hours of incubation with GPC3 (1:1000 molar ratio), highlighting a shift in the amide I band consistent with β sheet formation. (**I**) Molecular simulation of the transformation of SPD1 into complex (i.e., SPD1 nanoparticles) in a water box based on the hydrophobic core of PpIX molecules, along with a hydrophilic corona formed by GPC3-targeted ligands. (**J**) Molecular docking simulation for SPD1 and GPC3. Rectangle: the possible binding sites between SPD1 and GPC3. (**K**) MD simulation of fibrillar transformation of SPD1 generated at *t* = 250 ns after interaction with GPC3. Rectangle: the interaction forces of the hydrogen bond and π-π stacking. All experiments were independently repeated three times with consistent and reproducible results.

SPD1, produced through the 1:1 coassembly of SPDa and SPDb, served as the primary functional peptide material, enabling both cell membrane editing and bioorthogonal click chemistry reactivity. By contrast, SPD2, assembled solely from SPDb, functioned as a nontargeting control that retained only click chemistry reactivity. In aqueous solution, SPD1 spontaneously self-assembled into core-shell micellar nanoparticles, driven by the amphiphilic structure comprising a hydrophilic GPC3-targeting peptide and hydrophobic PpIX. Similarly, SPD2 formed into nanoparticles through hydrophilic (DBCO) and hydrophobic (PpIX) interactions. To monitor the self-assembly process, ultraviolet-visible (UV-vis) and fluorescence spectroscopy were used on the basis of the aggregation-caused quenching (ACQ) behavior of PpIX. In a 50 μM SPD1 solution [H_2_O/dimethyl sulfoxide (DMSO)], characteristic absorption bands (300 to 600 nm) and fluorescence emission at 640 and 700 nm were observed (Fig. 2, A and B). Both absorption and fluorescence intensities diminished with increasing H_2_O content, indicating progressive PpIX aggregation and the transition from monomers to nanoparticles. Critical aggregation concentrations (CACs), determined by UV-vis spectroscopy, were 5.5 μM for SPD1 (Fig. 2C) and 9.8 μM for SPD2 (fig. S9A), confirming their intrinsic propensity for self-assembly in aqueous media. Dynamic light scattering (DLS) measurement yielded hydrodynamic diameters of ~28 nm for SPD1 nanoparticles and ~25 nm for SPD2 nanoparticles, with zeta potentials of 5.9 mV for SPD1 nanoparticles and −5.4 mV for SPD2 nanoparticles (fig. S10). Transmission electron microscopy (TEM) further verified the spherical morphology of both SPD1 and SPD2 nanoparticles (Fig. 2D and fig. S9B).

Upon incubation with hydrosoluble recombinant GPC3 proteins, SPD1 nanoparticles underwent a secondary transition into nanofibrils, driven by β sheet formation of the FF peptide and specific ligand-receptor interactions with GPC3. To optimize the conditions for ligand-receptor–mediated morphological transformation in vitro, SPD1 nanoparticles were incubated with recombinant human GPC3 protein at varying molar ratios and time intervals. As shown in Fig. 2E, TEM imaging revealed a gradual progression from spherical nanoparticles to fibrillar networks upon GPC3 engagement, with a minimum molar ratio of 1:1000 (GPC3:SPD1) sufficient to induce extensive nanofiber formation. Time-dependent studies further demonstrated that spherical SPD1 nanoparticles evolved into elongated fibrillar structures between 0.5 and 6 hours, ultimately forming well-defined nanofibrils after 24 hours of incubation (Fig. 2F). Notably, TEM analysis under physiologically mimicking conditions further confirmed that SPD1 nanoparticles undergo GPC3-induced fibrillar transformation in vitro. In phosphate-buffered saline (PBS) supplemented with 10% fetal bovine serum (FBS), SPD1 initially maintained a spherical nanoparticle morphology, whereas incubation with human GPC3 triggered a progressive, time-dependent transition into elongated nanofibrillar structures. The fibrillar networks became increasingly pronounced with prolonged incubation, demonstrating that GPC3 alone is sufficient to induce SPD1 fibrillation even in a protein-rich environment (fig. S11). As a negative control, SPD2 nanoparticles, lacking the GPC3-targeting motif, exhibited no detectable morphological alterations after 24 hours of incubation with recombinant GPC3 (fig. S12).

The binding affinity between SPD1 and recombinant human GPC3 was quantified using biolayer interferometry (BLI). The association rate constant (*k*_on_) was (8.6 ± 0.1) × 10^3^ M^−1^ s^−1^, whereas the dissociation rate constant (*k*_dis_) was (9.5 ± 0.4) × 10^−4^ s^−1^. The equilibrium dissociation constant (*K*_D_) was calculated to be 109.7 ± 4.8 nM (fig. S13), indicating rapid and high-affinity binding between SPD1 and GPC3.

Circular dichroism (CD) spectroscopy revealed a clear secondary structure transition in SPD1 nanoparticles following 24 hours of incubation with recombinant human GPC3. As shown in Fig. 2G, a characteristic β sheet signature emerged, evidenced by a negative peak at ~215 nm and a positive peak at ~195 nm. In contrast, initial SPD1 nanoparticles exhibited a random coil-like spectrum with a negative band near ~200 nm, indicating that the assemblies were initially disordered before protein engagement. Quantitative CD analysis further demonstrated a time-dependent increase in β sheet content: Initial SPD1 nanoparticles displayed only 18.1% β sheet structure, which increased to 38.6, 45.6, 53.3, and 58.1% after 0.5, 6, 12, and 24 hours of incubation with GPC3, respectively (fig. S14). As expected, SPD2 nanoparticles showed no detectable spectral changes regardless of incubation time, confirming the requirement of GPC3 recognition for conformational rearrangement (fig. S15). Fourier transform infrared (FTIR) spectroscopy provided additional evidence of β sheet formation. After 24 hours of incubation with GPC3, the amide I band of SPD1 shifted from 1665 to 1641 cm^−1^ (Fig. 2H), consistent with the establishment of intermolecular hydrogen bonding characteristic of β sheet structures. No comparable spectral shift was observed for SPD2 under identical conditions (fig. S16). To evaluate the intrinsic structural stability of the nanoparticles, DLS measurements were performed in serum-containing medium. Both SPD1 and SPD2 maintained stable hydrodynamic diameters (~28 and ~25 nm, respectively) over 24 hours (fig. S17, A and B). TEM imaging further confirmed that both systems retained spherical morphology at room temperature in the absence of protein stimuli (fig. S17, C and D), indicating that the fibrillar transformation is specifically triggered by ligand-receptor interactions rather than nonspecific aggregation.

### All-atom MD simulations of SPD1 self-assembly and GPC3-induced fibrillar transformation

All-atom molecular dynamics (MD) simulations were used to obtain a microscopic description of SPD1 intermolecular interactions, encompassing its spontaneous self-assembly in aqueous solution, the binding interface with GPC3, and the structural evolution underlying ligand-induced fibrillar transformation.

During a 100-ns MD simulation, eight SPD1 molecules spontaneously organized into spherical nanoparticles, with PpIX moieties forming a compact hydrophobic core (Fig. 2I). Structural analysis revealed substantial fluctuations in the early stages, followed by equilibration at ~60 ns. Between 80 and 100 ns, the nanoparticle displayed stable structural properties: The root mean square deviation (RMSD) averaged 6.36 nm (fig. S18), the solvent-accessible surface area (SASA) averaged 185.80 nm^2^ (fig. S19), and the radius of gyration (Rg) averaged 2.29 nm (fig. S20). The number of hydrogen bonds increased rapidly during the assembly process and stabilized at ~74 within the same equilibrium window (fig. S21). Together, these results confirm the formation of a structurally stable SPD1 nanoparticle by the end of the simulation.

Before MD simulation of the protein-peptide complex, molecular docking was conducted to identify the interaction patterns between SPDa and GPC3 [Protein Data Bank (PDB) ID: 7ZA1]. SPDa exhibited a binding energy of −9.451 kcal/mol and docked into a cavity formed by the C, F, and H chains of GPC3 (Fig. 2J). Multiple short-range interactions contributed to the stabilization of the complex. Trp^89^ (chain H) and Gln^120^ (chain H) engaged the PpIX moiety of SPDa at bond lengths of 2.8 and 2.7 Å, respectively. His^125^ (chain H) formed a hydrogen bond with His^5^ of SPDa (1.8 Å), whereas Glu^160^ (chain H) interacted with the NH_2_ terminus of SPDa (2.4 Å). Arg^156^ (chain H) formed hydrogen bonds with Trp^10^ and Gly^12^ of SPDa (2.5 and 2.4 Å, respectively). In addition, Ser^293^ (chain F) engaged with Ser^8^ of SPDa (2.2 Å), and Asp^160^ (chain C) interacted with Thr^13^ of SPDa (2.3 Å). Arg^227^ (chain C) established three hydrogen bonds with Glu^14^ of SPDa (2.2, 2.3, and 2.5 Å). Collectively, these interactions provide strong evidence for the stable binding of SPDa to GPC3.

As shown in Fig. 2K, SPD1 underwent ordered nanofibrillar aggregation upon engagement with GPC3, driven by extensive hydrogen bonding and π-π stacking. MD simulations further corroborated the structural stabilization of the GPC3-SPD1 complex. The RMSD exhibited pronounced fluctuations from 0 to 150 ns, followed by a gradual increase and subsequent plateau between 150 and 190 ns, eventually stabilizing with an average value of 2.28 nm during the 200- to 250-ns interval (fig. S22A). Consistently, SASA decreased steadily and reached a stable average of 1324.69 nm^2^ in the same terminal interval (fig. S22B). The Rg fluctuated markedly during the initial 0 to 150 ns, subsequently declined toward a plateau between 150 and 190 ns, and stabilized at 5.26 nm over 200 to 250 ns (fig. S22C). Hydrogen bond analysis revealed a relatively constant number of hydrogen bonds between 150 and 204 ns, followed by a pronounced increase after 204 ns, indicating the engagement of additional binding sites as fibrillar ordering progressed (fig. S22D). Collectively, these data demonstrate that SPD1 binds GPC3 to form a stable nanofibrillar complex, consistent with ligand-induced conformational reorganization observed experimentally.

### Bioorthogonal conjugation of Gd-DOTA-N_3_ to pretargeted nanofibers enhances MRI relaxivity

**Fig. 3.**
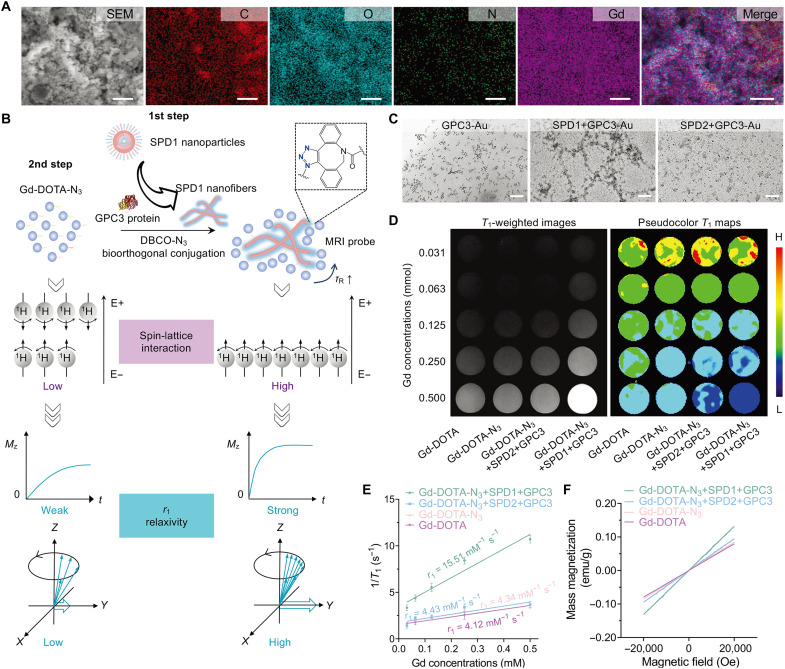
Bioorthogonal conjugation of Gd-DOTA to pretargeted nanofibers enhances MRI contrast performance. (**A**) SEM-EDX elemental mapping of the Gd-DOTA-N_3_+SPD1+GPC3 probe. False-color maps show the spatial distribution of carbon (C, red), oxygen (O, cyan), nitrogen (N, green), and Gd (magenta), confirming uniform Gd incorporation and colocalization with organic matrix elements. Scale bars, 25 μm. All experiments were independently repeated three times with consistent and reproducible results. (**B**) Schematic illustration of the covalent conjugation attachment of Gd-DOTA-N_3_ to SPD1 nanofibers via SPAAC click chemistry, designed to enhance *r*_1_ relaxivity. (**C**) TEM images showing GPC3-modified gold (Au) nanoparticles alone or after incubation with SPD1 or SPD2 nanoparticles (50 μM, 24 hours). Scale bars, 50 nm. (**D**) *T*_1_-weighted images and pseudocolor *T*_1_ maps comparing Gd-DOTA, Gd-DOTA-N_3_, Gd-DOTA-N_3_+SPD2-GPC3 (noncovalent incubation, molar ratio of 1000:1, 24 hours) for 6 hours, and Gd-DOTA-N_3_+SPD1-GPC3 (bioorthogonal conjugation, molar ratio of 1000:1, 24 hours) for 6 hours. Experiments were independently repeated three times, with consistent results observed across replicates. (**E**) *r*_1_ relaxivity of Gd-DOTA (purple), Gd-DOTA-N_3_ (pink), Gd-DOTA-N_3_+SPD2+GPC3 (blue), and Gd-DOTA-N_3_+SPD1+GPC3 (light green). Data are presented as means ± SD (*n* = 3 independent experiments). (**F**) Room-temperature VSM showing the enhanced paramagnetic signal from Gd-DOTA-N_3_+SPD1+GPC3 (light green), compared to Gd-DOTA-N_3_+SPD2+GPC3 (blue), Gd-DOTA-N_3_ (pink), and Gd-DOTA (purple).

Scanning electron microscopy coupled with energy-dispersive x-ray (SEM-EDX) spectroscopy confirmed the successful incorporation of Gd within the pretargeted nanofibers. Elemental mapping demonstrated uniform colocalization of carbon (C), oxygen (O), nitrogen (N), and Gd signals (Fig. 3A), indicating homogeneous integration of the Gd chelates within the nanofibrous structures. Such compositional uniformity is essential for maximizing *r*_1_ relaxivity.

To enhance *T*_1_ relaxivity, Gd-DOTA-N_3_ was covalently conjugated to DBCO-functionalized SPD1 nanofibers via SPAAC click chemistry (Fig. 3B). This bioorthogonal conjugation immobilized the Gd chelates on the rigid nanofibrillar scaffold, thereby prolonging τ_R_ and consequently enhancing *r*_1_ relaxivity and *T*_1_-weighted MRI signal. To visualize the spatial distribution of nanofibrils, GPC3-modified gold (Au) nanoparticles were used as surrogates for Gd chelates. TEM showed uniform dispersion of GPC3-Au nanoparticles in solution, with no aggregation (Fig. 3C, left). Upon incubation with SPD1 (50 μM, 24 hours), the GPC3-Au nanoparticles reorganized along fibrillar structures, demonstrating ligand-specific binding and nanofiber-guided spatial localization (Fig. 3C, middle). In contrast, SPD2 (50 μM, 24 hours) lacking the GPC3-targeting motif showed negligible interaction with GPC3-Au nanoparticles under identical conditions (Fig. 3C, right), confirming the specificity of SPD1-mediated molecular recognition.

MRI was then used to evaluate the contrast enhancement achieved by the bioorthogonally conjugated system. As shown in Fig. 3D, *T*_1_-weighted images and quantitative *T*_1_ maps revealed markedly stronger signal intensity and shorter *T*_1_ values in the Gd-DOTA-N_3_+SPD1+GPC3 group compared with the three control groups (Gd-DOTA-N_3_+SPD2+GPC3, free Gd-DOTA-N_3_, and clinical Gd-DOTA). Quantification of *r*_1_ relaxivity demonstrated that the Gd-DOTA-N_3_+SPD1+GPC3 group exhibited the highest *r*_1_ value of 15.51 mM^−1^ s^−1^ compared to the three control groups (*r*_1_ = 4.43, 4.34, and 4.12 mM^−1^ s^−1^, respectively, increased by 3.5-, 3.6-, and 3.8-fold; Fig. 3E). These data establish that pretargeted nanofibrillar immobilization markedly amplifies the MRI relaxivity of Gd chelates.

The paramagnetic properties of the bioorthogonally conjugated probe were further examined using vibrating sample magnetometry (VSM). The Gd-DOTA-N_3_+SPD1+GPC3 group showed a comparable magnetic response [Ms ≈ 0.131 electromagnetic units (emu)/g] to the control groups (Ms ≈ 0.090, 0.084, and 0.079 emu/g), indicating that the bioorthogonal conjugation preserves the intrinsic paramagnetic behavior of Gd-DOTA (Fig. 3F).

### Formation of the fibrillar skeleton on the membrane of HCC cells

**Fig. 4.**
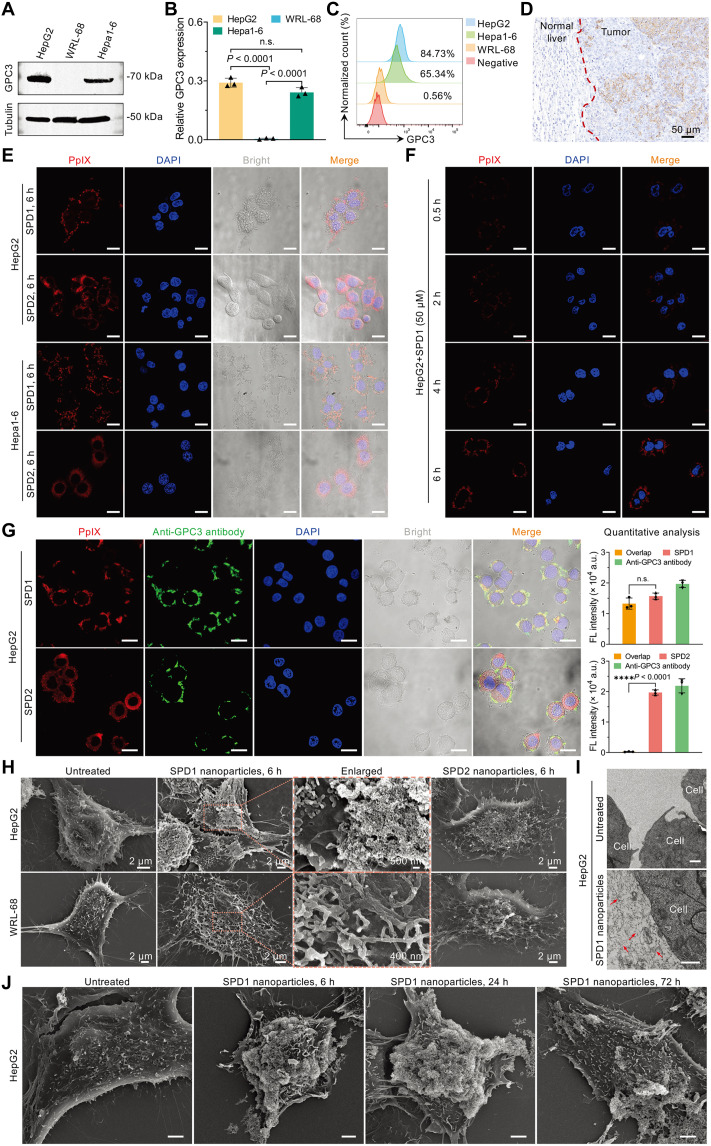
Formation of membrane-associated fibrillar networks on the HCC cell surface. (**A**) Representative Western blot analysis of GPC3 expression in HepG2, WRL-68, and Hepa1-6 cells (*n* = 3 independent experiments). (**B**) Quantitation of relative GPC3 protein level from (A). Data are presented as means ± SD (*n* = 3). Statistical analysis was performed using one-way ANOVA with a Tukey’s post hoc test. (**C**) Flow cytometry analysis of surface GPC3 expression in HepG2, WRL-68, and Hepa1-6 cells. (**D**) Representative IHC images of GPC3 expression (brown) in tumor tissues from orthotopic Hepa1-6 tumor-bearing mice. Scale bar, 50 μm. (**E**) CLSM images of HepG2 and Hepa1-6 cells treated with SPD1 or SPD2 nanoparticles (50 μM; red fluorescence) for 6 hours. Scale bars, 20 μm. (**F**) Time-dependent CLSM imaging of HepG2 cells treated with SPD1 nanoparticles (50 μM) showing membrane-localized fibrillar transformation. Scale bars, 20 μm. (**G**) CLSM analysis of HepG2 cells sequentially incubated with SPD1 or SPD2 nanoparticles (50 μM, 6 hours; red) and FITC-labeled anti-GPC3 antibody (green; 1:200; Abcam, #ab207080). Colocalization (yellow) indicates specific binding of SPD1 to membrane-bound GPC3. Fluorescence intensity and colocalization were quantified using MATLAB. Data are presented as means ± SD (*n* = 3); n.s., not significant (one-way ANOVA with Tukey’s post hoc test). Scale bars, 20 μm. (**H**) SEM images of untreated HepG2 and WRL-68 cells or incubated with SPD1 or SPD2 nanoparticles (50 μM, 6 hours). Magnified insets highlight membrane-associated fibrillar structures. (**I**) TEM images of untreated HepG2 cells (top) and those treated with SPD1 nanoparticles (50 μM, 24 hours; bottom). Red arrows indicate membrane-associated nanofibers. Scale bars, 500 nm. (**J**) SEM images showing the persistence of SPD1-derived fibrillar networks on HepG2 cells at 6, 24, and 72 hours posttreatment (50 μM). Scale bars, 2 μm. All experiments were independently repeated three times with consistent and reproducible results.

To investigate the fibrillar transformation of SPD1 nanoparticles on the membrane of HCC cells, we first examined GPC3 expression in representative human and murine HCC cells. Western blotting showed significantly higher GPC3 levels in human hepatoblastoma G2 (HepG2) and murine hepatoma 1-6 (Hepa1-6) cells compared to the human fetal hepatic epithelial cell line WRL-68 (*P* < 0.0001 for both comparisons; Fig. 4, A and B). Flow cytometry further confirmed these differences, revealing abundant GPC3 expression in HepG2 and Hepa1-6 cells but minimal expression in WRL-68 cells (Fig. 4C), consistent with the results from immunofluorescence (IF) staining (fig. S23). In orthotopic Hepa1-6 tumor-bearing mice, immunohistochemistry (IHC) demonstrated strong GPC3 staining in tumor tissues with negligible signal in adjacent normal liver tissue (Fig. 4D).

Following incubation with SPD1 nanoparticles (50 μM, 6 hours), HepG2 and Hepa1-6 cells exhibited intense PpIX-derived red fluorescence predominantly restricted to the plasma membrane, as visualized by confocal laser scanning microscopy (CLSM) (Fig. 4E). In contrast, WRL-68 cells displayed mainly cytoplasmic fluorescence under identical conditions (fig. S24). Notably, membrane-associated fibrillar structures were already evident in HepG2 cells within 6 hours of SPD1 incubation (Fig. 4F). Inhibitor studies demonstrated that SPD1 internalization was substantially reduced by β-cyclodextrin (β-CD; 5 mM), amiloride (2 mM), and hypertonic sucrose (450 mM) (fig. S25), indicating that uptake proceeds through clathrin-, caveolae-, and/or macropinocytosis-mediated endocytic pathways. In contrast, SPD2 nanoparticles accumulated intracellularly in all tested cell lines (HepG2, Hep1-6, and WRL-68) without notable membrane enrichment. Similar inhibition profiles were observed for SPD2 following treatment with β-CD, amiloride, and hypertonic sucrose (fig. S26), suggesting that SPD1 and SPD2 share common endocytic pathways.

To confirm the specificity of SPD1 binding to membrane-localized GPC3, we performed dual-channel CLSM in HepG2 cells. IF staining of GPC3 (green) and PpIX fluorescence from SPD1 (red) showed pronounced colocalization on the plasma membrane (yellow) (Fig. 4G, top row). Quantitative analysis of membrane fluorescence overlap using MATLAB confirmed significant colocalization between SPD1 and GPC3 signals, supporting GPC3-dependent membrane targeting. In contrast, SPD2-treated cells exhibited minimal overlap between PpIX and GPC3 fluorescence (Fig. 4G, bottom row).

SEM revealed well-defined fibrillar networks on the surface of HepG2 cells after 6 hours of SPD1 incubation (Fig. 4H). These membrane-associated structures were not observed in SPD2-treated HepG2 cells or in WRL-68 cells treated with either SPD1 or SPD2. TEM further confirmed the presence of extensive nanofiber bundles on and between HepG2 cell membranes following 24 hours of SPD1 treatment, whereas no such structures were detected in untreated control cells (Fig. 4I).

To characterize the persistence of these membrane-associated nanofibrils, HepG2 cells were imaged 24 to 72 hours after SPD1 treatment. CLSM revealed sustained membrane fluorescence for up to 72 hours (fig. S27), and SEM confirmed the structural integrity of the fibrillar networks at this time point (Fig. 4J). Notably, Western blotting analysis revealed that the prolonged retention of fibrillar structures on the HepG2 cell membrane did not result in any significant alteration in GPC3 expression levels (fig. S28, A and B). By contrast, most internalized SPD2 nanoparticles underwent lysosomal degradation after 24 hours (fig. S29). Collectively, these findings demonstrate that SPD1 nanoparticles selectively target GPC3-overexpressing tumor cells and undergo receptor-mediated transformation into stable membrane-associated fibrillar scaffolds.

### Cellular MRI performance of the bioorthogonally assembled fibrillar-Gd-DOTA-N_3_ probe

**Fig. 5.**
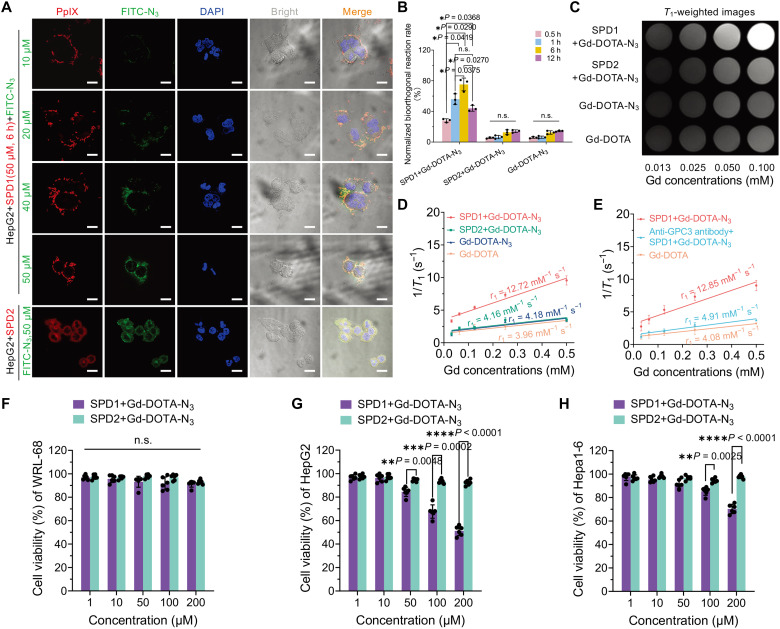
Cellular MRI performance and biosafety of the bioorthogonally assembled nanofibril-Gd-DOTA probe. (**A**) Representative CLSM images of HepG2 cells incubated with SPD1 nanoparticles (red, 50 μM) for 6 hours, followed by treatment with FITC-N_3_ (10 to 50 μM, green) for an additional 6 hours. Merged yellow fluorescence indicates successful copper-free click conjugation between DBCO and N_3_ on the cell membrane. Cells treated with SPD2+FITC-N_3_ (50 μM) served as the nontargeted controls, showing minimal colocalization. Scale bars, 20 μm. (**B**) Time-dependent kinetics of bioorthogonal conjugation quantified by BCA protein assay and ICP-MS. HepG2 cells were pretreated with SPD1 or SPD2 nanoparticles (50 μM, 6 hours), followed by incubation with Gd-DOTA-N_3_ (50 μM) for 0.5, 1, 6, or 12 hours. Cells treated with Gd-DOTA-N_3_ alone served as baseline controls. (**C**) *T*_1_-weighted MR images of HepG2 cells treated with Gd-DOTA (50 μM), Gd-DOTA-N_3_ (50 μM), or sequentially with SPD1 or SPD2 (50 μM, 6 hours) followed by Gd-DOTA-N_3_ (50 μM, 6 hours). (**D**) Quantitative *r*_1_ relaxivity under the corresponding treatment conditions in (C). (**E**) *r*_1_ relaxivity of HepG2 cells preblocked with anti-GPC3 antibody (5 μg/ml, 12 hours; Abcam, #ab207080) before SPD1 treatment (50 μM, 6 hours) followed by Gd-DOTA-N_3_ (50 μM, 6 hours). (**F** to **H**) Cell viability of WRL-68 (F), HepG2 (G), and Hepa1-6 (H) cells after sequential treatment with SPD1 or SPD2 for 6 hours followed by Gd-DOTA-N_3_ (50 μM, 6 hours). Cell viability was quantified using the CCK-8 assay. Data are presented as means ± SD {*n* = 3 for [(A) to (E)]; *n* = 6 for [(F) to (H)]}. Statistical significance was performed using one-way ANOVA followed by Tukey’s post hoc test. *P* < 0.05 was considered statistically significant; n.s., not significant. All experiments were independently repeated three times with consistent results.

To validate membrane localization and bioorthogonal conjugation of Gd-DOTA-N_3_, SPD1 nanoparticles were incubated with HepG2 cells for 6 hours to allow for cell-surface nanofiber formation. Subsequent treatment with fluorescein isothiocyanate (FITC)–N_3_, a fluorescent analog of Gd-DOTA-N_3_, produced concentration-dependent and membrane-restricted colocalization with SPD1 (red), as indicated by merged yellow fluorescence in CLSM images (Fig. 5A). In contrast, FITC and PpIX signals in SPD2-treated HepG2 cells were diffusely distributed within the cytoplasm.

To quantify conjugation kinetics, HepG2 cells were sequentially treated with SPD1 or SPD2 followed by Gd-DOTA-N_3_, and conjugation efficiency was determined using protein quantification and inductively coupled plasma mass spectrometry (ICP-MS). SPD1-treated cells showed a time-dependent increase in normalized Gd content, reaching a maximum at 6 hours before declining (*P* = 0.0222 to 0.0375). In contrast, SPD2-treated cells showed negligible Gd conjugation throughout the time course (*P* > 0.05) (Fig. 5B). These results confirmed both the spatial specificity and the temporal characteristic of the membrane-localized bioorthogonal reaction in living HCC cells.

The cellular MRI performance of the assembled probe was then evaluated. HepG2 cells treated with SPD1 (50 μM, 6 hours) followed by Gd-DOTA-N_3_ (50 μM, 6 hours) displayed markedly increased *T*_1_-weighted MR signal intensity and enhanced *r*_1_ relaxivity (12.72 mM^−1^ s^−1^), compared with Gd-DOTA (*r*_1_ = 3.96 mM^−1^ s^−1^), Gd-DOTA-N_3_ (*r*_1_ = 4.18 mM^−1^ s^−1^), or SPD2+Gd-DOTA-N_3_ (*r*_1_ = 4.16 mM^−1^ s^−1^; Fig. 5, C and D). The enhancement was GPC3-dependent, as preblocking with an anti-GPC3 antibody reduced *r*_1_ relaxivity to 4.91 mM^−1^ s^−1^, comparable to nontargeted controls (Fig. 5E).

The cytotoxicity of the probe system was evaluated in normal and HCC cell lines using CCK-8 assays after 24 hours of incubation. SPD1+Gd-DOTA-N_3_ caused no detectable cytotoxicity in normal WRL-68 cells over a concentration range of 1 to 200 μM (*P* > 0.05; Fig. 5F). By contrast, significant dose-dependent cytotoxicity occurred in HepG2 (*P* = 0.0002 to *P* < 0.0001) and Hepa1-6 cells (*P* = 0.0025 to *P* < 0.0001) at 50 to 200 μM (Fig. 5, G and H). SPD2+Gd-DOTA-N_3_ elicited no cytotoxic effects in any tested cell at any concentrations (*P* > 0.05) (Fig. 5, F to H). Two-way analysis of variance (ANOVA) further confirmed no significant effects of Gd-DOTA-N_3_ or Gd-DOTA on the viabilities of WRL-68, HepG2, or Hepa1-6 cells (all *P* > 0.05; fig. S30), indicating that both Gd-DOTA-N_3_ and Gd-DOTA are well tolerated by normal hepatocytes and liver cancer cells up to 100 μM.

### Pharmacokinetics, biodistribution, and in vivo MRI evaluation of the SPD1+Gd-DOTA-N_3_ probe

**Fig. 6.**
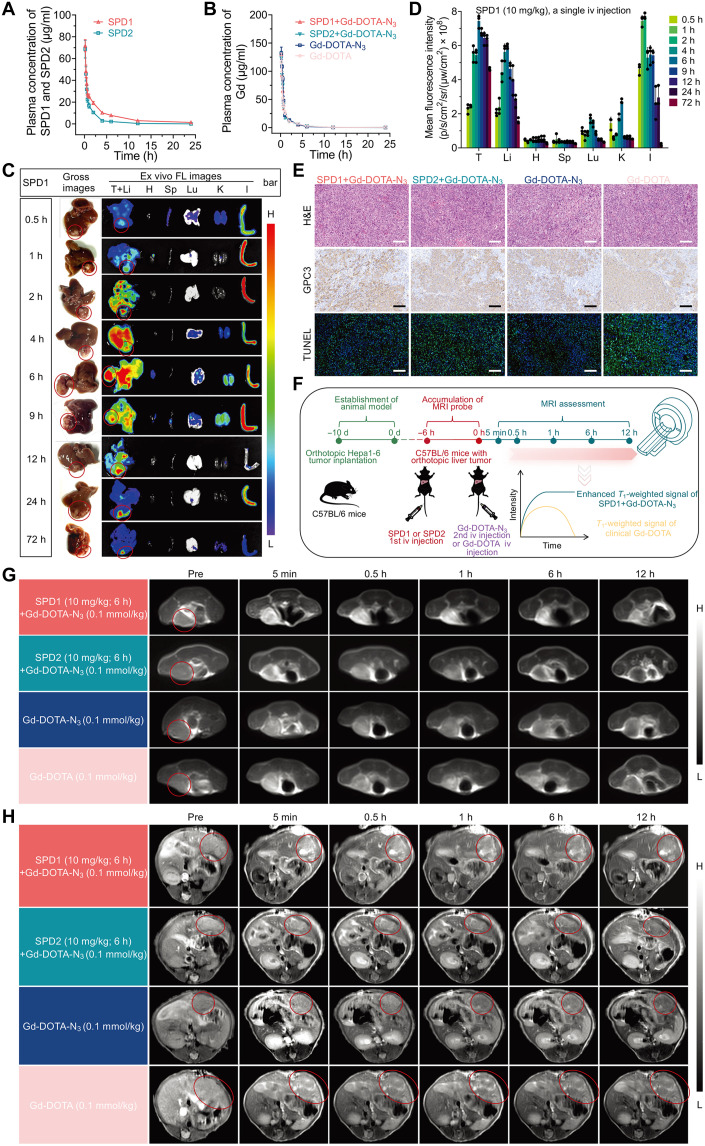
Pharmacokinetics, biodistribution, and MRI performance of the bioorthogonally assembled SPD1-Gd-DOTA-N_3_ molecular probe. (**A**) Pharmacokinetic profiles of SPD1 [10 mg/kg, intravenously (iv)] and SPD2 (10 mg/kg, iv) in healthy mice. Plasma concentrations were determined via PpIX fluorescence, and pharmacokinetic parameters were calculated using PKSolver 2.0 software. Data are expressed as means ± SD (*n* = 3). (**B**) Blood Gd^3+^ levels measured by ICP-MS in orthotopic Hepa1-6 tumor-bearing mice after Gd-DOTA-N_3_ administration (0.1 mmol/kg, iv), with or without 6 hours pretreatment with SPD1 or SPD2 (10 mg/kg, iv). (**C**) Ex vivo fluorescence (FL) images of the tumors (T), heart (H), liver (Li), spleen (Sp), lung (Lu), kidney (K), and intestine (I), at indicated time points following SPD1 injection (10 mg/kg, iv) in orthotopic Hepa1-6 tumor-bearing mice. Red dashed circles indicate tumors. Representative images from three mice per time point are shown. (**D**) Quantification of the fluorescence intensity in tumor and major organs of (C). (**E**) Representative micrographs demonstrate H&E staining, GPC3 IHC, and TUNEL staining in orthotopic Hepa1-6 tumor specimens from C57BL/6 mice treated with the following regimens: SPD1 pretreatment followed by Gd-DOTA-N_3_ 6 hours later, SPD2 pretreatment followed by Gd-DOTA-N_3_ 6 hours later, Gd-DOTA-N_3_ alone, and Gd-DOTA alone. Representative images are shown from three biologically independent experiments. Scale bars, 100 μm. (**F**) Schematic illustration of the molecular imaging mechanism of the SPD1+Gd-DOTA-N_3_ probe, highlighting self-assembly into nanofibers and signal amplification via *r*_1_ enhancement. d, days. (**G**) In vivo *T*_1_-weighted images of subcutaneous Hepa1-6 tumor-bearing mice at multiple time points under four different experimental protocols. Red circles mark tumor regions. Representative images from three mice per group are shown. (**H**) In vivo *T*_1_-weighted images of orthotopic Hepa1-6 tumor-bearing mice under four different treatment protocols. Red circles mark tumor regions. Representative images from three mice per group are shown.

The pharmacokinetic profiles of SPD1 and SPD2 were evaluated in healthy C57BL/6 mice following a single intravenous administration (10 mg/kg). Plasma concentrations were quantified on the basis of the fluorescence of the PpIX moiety. As shown in Fig. 6A and table S1, SPD1 exhibited superior pharmacokinetic performance compared with SPD2, including a higher maximum plasma concentration (*C*_max_ = 71.76 ± 5.54 μg/ml versus 68.65 ± 8.15 μg/ml; *P* = 0.614), a significantly increased area under the concentration-time curve from 0 to 24 hours (AUC_0–24_ = 164.43 ± 3.96 μg·hour/ml versus 79.96 ± 4.06 μg·hour/ml; *P* < 0.0001), a prolonged elimination half-life (*t*_1/2Z_ = 6.29 ± 0.96 hours versus 3.58 ± 1.00 hour; *P* = 0.028), a significantly reduced systemic clearance (CL = 1.13 ± 0.02 ml/hour per kilogram versus 11.09 ± 1.19 ml/hour pe kilogram; *P* < 0.0001), and a significantly extended mean residence time (MRT = 7.13 ± 0.27 hours versus 2.46 ± 0.13 hours; *P* < 0.0001).

To investigate the in vivo behavior of Gd, orthotopic Hepa1-6 tumor-bearing mice were randomly assigned to four treatment groups and received: (i) SPD1 (10 mg/kg) followed 6 hours later by Gd-DOTA-N_3_ (0.1 mmol/kg), (ii) SPD2 (10 mg/kg) followed 6 hours later by Gd-DOTA-N_3_ (0.1 mmol/kg), (iii) Gd-DOTA-N_3_ (0.1 mmol/kg) alone, or (iv) Gd-DOTA (0.1 mmol kg^−1^) alone. Plasma Gd concentrations were quantified by ICP-MS to derive the pharmacokinetic parameters. No significant differences were observed across groups in *C*_max_ (*P* = 0.4228), AUC_0–24_ (*P* = 0.7404), *t*_1/2Z_ (*P* = 0.3065), CL (*P* = 0.4475), or MRT (*P* = 0.3104) (Fig. 6B and table S2), indicating that neither SPD1 nor SPD2 alters the systemic pharmacokinetics of Gd-DOTA-N_3_ under the tested dosing conditions.

Ex vivo fluorescence imaging demonstrated distinct biodistribution profiles for SPD1 and SPD2 in orthotopic Hepa1-6 tumor-bearing mice. Following intravenous injection, SPD1 (10 mg/kg) exhibited rapid hepatic uptake with peak fluorescence at 6 hours postinjection and gradual clearance thereafter (Fig. 6C). Notably, SPD1 accumulated in tumors as early as 0.5 hours, reached maximal fluorescence at 6 hours, and remained elevated for up to 72 hours. Quantitative analysis showed significantly higher tumor fluorescence for SPD1 than for major organs, including the heart, spleen, lungs, and kidneys, between 2 and 72 hours postinjection (Fig. 6D). Although hepatic and intestinal signals remained dominant, tumor accumulation of SPD1 exceeded that of SPD2, consistent with enhanced GPC3-mediated fibrillar assembly at the tumor surface. Notably, hematoxylin and eosin (H&E)–stained sections of small intestine collected at multiple time points after sequential administration of SPD1 and Gd-DOTA-N_3_ showed preserved intestinal architecture, with intact villi and normal mucosal organization comparable to PBS-treated controls (fig. S31). These results indicate that the observed intestinal fluorescence accumulation does not elicit detectable adverse effects on normal tissue under the tested conditions. In addition, tumor tissue TEM images confirmed a time- and dose-dependent in situ fibrillar transformation of SPD1 nanoparticles at the tumor site. Following intravenous administration, SPD1 progressively assembled into membrane-associated nanofibrillar structures, which became denser with increasing circulation time. At 6 hours postinjection, higher doses (10 mg/kg) of SPD1 induced more extensive fibrillar networks along tumor cell membranes, whereas untreated tumors showed no fibrillar features (fig. S32, A and B). In contrast, SPD2 showed delayed tumor targeting, peaking at 9 hours followed by rapid clearance (figs. S33 and S34). These results demonstrate that the extent of SPD1 fibrillar assembly in tumors is jointly regulated by exposure time and administered dose.

Histopathological analyses confirmed no appreciable baseline differences among treatment groups (SPD1+Gd-DOTA-N_3_, SPD2+Gd-DOTA-N_3_, Gd-DOTA-N_3_ alone, and Gd-DOTA alone) (Fig. 6E). H&E staining demonstrated preserved tumor architecture, whereas IHC analysis of GPC3 confirmed homogeneous overexpression across groups. Terminal deoxynucleotidyl transferase–mediated deoxyuridine triphosphate nick end labeling (TUNEL) staining showed uniformly comparable baseline apoptosis. These results validate the orthotopic Hepa1-6 tumor model and establish a consistent histological context for evaluating probe-mediated MRI enhancement.

To assess biosafety, healthy mice and orthotopic Hepa1-6 tumor-bearing mice were administered SPD1 (10 mg/kg), followed by Gd-DOTA-N_3_ (0.1 mmol/kg) 6 hours later. Major organs, including the heart, liver, spleen, lungs, and kidneys, were harvested from healthy mice at 1, 3, 7, and 28 days postinjection and subjected to H&E staining. No discernible histopathological abnormalities were observed at any time point (fig. S35). To further assess systemic biosafety, complete blood counts and serum biochemical parameters were analyzed in both healthy mice and orthotopic Hepa1-6 tumor-bearing mice following treatment. Complete blood counts showed no abnormalities, and serum biochemical markers of liver function, such as alanine aminotransferase (ALT) and aspartate transaminase (AST), and renal function, such as blood urea nitrogen (BUN), creatinine (CR), and uric acid (UA), remained within physiological limits (figs. S36 and S37). Hemolysis assays showed negligible erythrocyte lysis (fig. S38), supporting excellent systemic tolerability. To assess the effect of SPD1 on immunocompatibility, core pro-inflammatory cytokines such as tumor necrosis factor–α (TNF-α), interleukin-6 (IL-6), and interleukin-1β (IL-1β) were measured in serum by enzyme-linked immunosorbent assay (ELISA). As shown in fig. S39 (A to C), no significant difference was found between mice treated with SPD1, SPD2, Gd-DOTA, Gd-DOTA-N_3_, and PBS 6 hours postinjection. Collectively, these results indicated a favorable in vivo safety profile of SPD1+Gd-DOTA-N_3_.

In vivo MRI was performed in both subcutaneous (Fig. 6G) and orthotopic (Fig. 6H) Hepa1-6 tumor-bearing mice following the protocol outlined in Fig. 6F. Preinjection of SPD1 (10 mg/kg, 6 hours) followed by Gd-DOTA-N_3_ (0.1 mmol/kg) produced substantially stronger and more persistent *T*_1_-weighted tumor enhancement compared with SPD2+Gd-DOTA-N_3_, Gd-DOTA-N_3_ alone, or Gd-DOTA alone. The SPD1+Gd-DOTA-N_3_ group exhibited the highest tumor-to-liver (T/L) signal intensity ratios (fig. S40). Macroscopic images revealed variably sized tumor nodules distributed across hepatic lobes, and H&E staining showed poorly differentiated HCC morphology characterized by dense cell packing, enlarged nuclei, and increased nuclear-to-cytoplasmic ratios (fig. S41). To validate the GPC3-dependent mechanism driving tumor-specific accumulation, a GPC3-blocking experiment was conducted. Pretreatment with anti-GPC3 antibody notably reduced *T*_1_ contrast enhancement following SPD1+Gd-DOTA-N_3_ administration (fig. S42), confirming the GPC3-targeting and in situ self-assembly.

ICP-MS quantitation at 1 hour postinjection confirmed markedly increased Gd accumulation in tumors treated with SPD1+Gd-DOTA-N_3_ (1.58 ± 0.11 μg/g), compared to SPD2+Gd-DOTA-N_3_ (0.87 ± 0.09 μg/g), Gd-DOTA-N_3_ alone (0.95 ± 0.08 μg/g), and Gd-DOTA alone (0.78 ± 0.07 μg/g) (figs. S43 and S44).

Given that SPD1+Gd-DOTA-N_3_ prolongs gadolinium retention at tumor sites, there is a potential concern regarding off-target gadolinium accumulation in the brain. Following intravenous injection, SPD1 exhibited negligible accumulation in the brain, with no discernible fluorescence signal detected in the head region across all examined time points (fig. S45). *T*_1_-weighted MRI of the head was performed in orthotopic Hepa1-6 tumor-bearing mice on day 7 after intravenous administration of SPD1+Gd-DOTA-N_3_, with SPD2+Gd-DOTA-N_3_, free Gd-DOTA-N_3_, and Gd-DOTA serving as controls. As shown in fig. S46, representative coronal images revealed comparable signal intensities across all treatment groups, with no abnormal hyperintense foci detected in the bilateral basal ganglia. These results indicate the absence of detectable long-term gadolinium deposition in the brain under any of the tested conditions.

## DISCUSSION

Accurate detection of micro-HCC remains a major clinical challenge as microlesions are strongly associated with curative treatment opportunities and near-normal long-term survival. However, currently available imaging modalities (ultrasonography, CT, and PET) are fundamentally constrained by spatial resolution or radiation burden, resulting in suboptimal sensitivity for early-stage hepatic nodules. Although MRI offers superior soft-tissue contrast, clinically approved GBCAs function predominantly as nonspecific extracellular tracers and exhibit intrinsically low *r*_1_ relaxivity. Even hepatobiliary-specific agents such as gadoxetate disodium fail to reliably detect micro-HCC. Collectively, these constrains highlight a persistent need for MRI probes that simultaneously achieve molecular specificity, signal amplification, and clinical feasibility.

In this work, we present a dual-injection peptidic MRI strategy based on in vivo membrane engineering, in which receptor-guided supramolecular transformation establishes a tumor-confined chemical interface for subsequent contrast-agent capture. Central to this approach is SPD1, a coassembled system composed of two transformable peptide monomers (SPDa and SPDb, mixed 1:1), incorporating a hydrophilic GPC3-targeting ligand (DHLASLWWGTEL) (*29*), a hydrophobic β sheet–forming motif (FF) (*42*), a PpIX-based assembly guide (*43*), and a bioorthogonal DBCO handle for copper-free click ligation (*44*). SPD2, which lacks the GPC3-targeting segment, serves as a stringent nontargeting control while preserving click reactivity. In aqueous environments, SPD1 self-assembles into stable spherical nanoparticles with a low CAC, supporting systemic delivery and tumor exposure (*22*). Upon engagement with membrane-expressed GPC3, these nanoparticles undergo a pronounced nanoparticle-to-nanofibril transition, accompanied by β sheet enrichment and persistent membrane anchoring. By contrast, the nontargeted control SPD2 remains morphologically unchanged, demonstrating that fibrillar transformation is strictly dependent on ligand-receptor recognition rather than nonspecific aggregation.

This receptor-triggered supramolecular reconfiguration represents a key mechanistic advance. BLI assays confirmed high-affinity binding between SPD1 and GPC3, providing a molecular basis for the selective membrane anchoring observed in cellular and in vivo contexts. MD simulations further revealed that SPD1 molecules rapidly assemble into stable nanoparticles in aqueous solution and reorganize into ordered nanofibers upon GPC3 engagement, consistent with prior reports on receptor-mediated peptide assembly (*20*–*22*). Mechanistically, the core advance of this work lies in coupling receptor-triggered fibrillar assembly to spatially confined bioorthogonal chemistry to amplify MRI performance. The membrane-anchored SPD1 nanofibrils present a high local density of exposed DBCO motifs, enabling efficient SPAAC with systemically administered Gd-DOTA-N_3_. This reaction immobilizes otherwise freely diffusing Gd chelates onto a rigid supramolecular scaffold, substantially prolonging the τ_R_ (*23*). As a result, the assembled probe exhibits a markedly enhanced *r*_1_ value (~15.51 mM^−1^ s^−1^), exceeding that of clinical Gd-DOTA by more than threefold, consistent with a previous study (*45*). SEM-EDX mapping confirmed homogeneous Gd distribution along the fibrillar scaffold, whereas VSM demonstrated that relaxivity enhancement arises from nanoscale confinement rather than altered paramagnetic properties.

Cellular studies further elucidate the spatial logic of signal amplification. In GPC3-overexpressing HCC cells, SPD1 preferentially localized to the plasma membrane and formed persistent fibrillar networks, whereas in GPC3-low or negative hepatocytes, it was predominantly internalized without productive assembly. Accordingly, FITC-N_3_ (as a surrogate for Gd-DOTA-N_3_) exhibited membrane-restricted colocalization only under the SPD1 condition. ICP-MS demonstrated time-dependent, markedly higher Gd accumulation in SPD1-treated HepG2 cells with an optimal conjugation window around 6 hours. Notably, this membrane engineering process was well tolerated by normal hepatocytes while exhibiting selective, dose-dependent cytotoxicity in GPC3-positive tumor cells, indicating that functional activity is restricted to pathological contexts.

A particularly important in vivo finding is the decoupling of systemic gadolinium exposure from tumor Gd enrichment. Despite nearly identical plasma pharmacokinetic profiles of Gd across all treatment groups, sequential administration of SPD1 followed by Gd-DOTA-N_3_ produced substantially higher intratumoral Gd accumulation and stronger *T*_1_-weighted enhancement than nontargeted or free GBCA controls. This observation supports a distinct targeting paradigm: SPD1 does not act by prolonging whole-body circulation of Gd chelates but instead establishes a tumor-localized capture interface after systemic distribution has occurred. In this framework, molecular specificity is transferred from a targeted peptide scaffold to an otherwise nontargeted small-molecule contrast agent via in situ chemical trapping. Such a mechanism is particularly attractive from a translational perspective as it avoids the prolonged systemic retention that often underlies off-target toxicity in macromolecular or long-circulating contrast platforms. In addition, in vivo fluorescence imaging in orthotopic HCC models also revealed rapid and sustained tumor accumulation of SPD1, with strong fluorescence signals detectable as early as 0.5 hours postinjection and persisting beyond 72 hours. Consistent with this concept, off-target deposition patterns remained comparable to clinically used Gd-DOTA under the tested conditions, and no evidence of histopathological injury, hematological abnormalities, or hepatic/renal dysfunction was detected up to 28 days after administration.

Notably, the 6-hour interval between SPD1 and Gd-DOTA-N_3_ administration represents a critical design parameter of this pretargeted system. This timing was selected on the basis of in vitro assembly kinetics and in vivo tumor-accumulation profiles, which indicate that SPD1 requires ~4 to 6 hours to complete receptor-mediated membrane anchoring and nanofibrillar formation. Shorter intervals result in incomplete exposure of DBCO groups and reduced conjugation efficiency, whereas extending the interval beyond 6 hours provided no additional benefit in pilot studies. From a translational perspective, this two-step strategy is conceptually aligned with clinically established pretargeting approaches in nuclear imaging and antibody-based diagnostics, where delayed payload administration is routinely used to maximize target specificity while minimizing systemic exposure. The specific interval used here reflects murine pharmacokinetics and can be readily adjusted during clinical translation to accommodate species-dependent differences in circulation half-life and tumor accessibility.

This study has several limitations. First, partial reliance on EPR-mediated tumor accumulation may overestimate performance in murine models relative to patients, necessitating validation in more heterogeneous and clinically relevant settings. Second, although SPAAC is bioorthogonally compatible, reaction efficiency in vivo may be influenced by diffusion constraints, steric accessibility of DBCO on the fibrils, and potential background interactions, underscoring the importance of optimizing dosing, timing, and linker presentation to maximize tumor-to-background ligation. Third, the “micro-HCC” model in mice does not fully recapitulate the extreme spatial constraints of human microlesions. Last, the incorporation of a PpIX motif introduces a theoretical risk of photosensitivity, which could be mitigated in future designs by substituting alternative hydrophobic assembly guides or therapeutic payloads.

In conclusion, this work demonstrates that in vivo membrane engineering can be leveraged to create a tumor-specific supramolecular scaffold that captures extracellular Gd chelates in situ, thereby simultaneously addressing the two principal limitations of conventional GBCAs: poor molecular specificity and low *r*_1_ relaxivity. By integrating receptor-guided fibrillar assembly with spatially confined bioorthogonal conjugation, the SPD1/Gd-DOTA-N_3_ system enables robust *T*_1_ signal amplification and high signal contrast for the detection of micro-HCC while maintaining a favorable safety profile. Given the modularity of the peptide design and the generality of click chemistry, this platform is readily adaptable to other tumor biomarkers and imaging payloads and may ultimately support multifunctional, image-guided strategies spanning early diagnosis and precision therapy.

## MATERIALS AND METHODS

### Ethics statement

All animal experiments were approved by the Laboratory Animal Welfare and Ethics Committee of Southern University of Science and Technology (SUSTech-JY202507004) and conducted in accordance with institutional ethical guidelines. Mice were housed under specific pathogen–free (SPF) conditions (20° to 22°C, 30 to 70% humidity, and 12-hour light/12-hour dark cycle) with ad libitum access to food and sterile water. Tumors were monitored daily, and mice were euthanized via CO_2_ asphyxiation followed by cervical dislocation upon reaching a maximum tumor diameter of 10 mm or immediately after MRI examinations.

### Cell lines

Human HepG2 (ATCC HB-8065), human WRL-68 (ATCC CL-48), and mouse Hepa1-6 (ATCC CRL-1830) were purchased from the American Type Culture Collection (ATCC) and cultured in Dulbecco’s modified Eagle’s medium (DMEM) (Gibco, C11995500BT) supplemented with 10% FBS (Gibco, A3161001C) and 1% penicillin/streptomycin (Beyotime, C0222) at 37°C in a humidified 5% CO_2_ atmosphere. All cell lines were routinely screened for mycoplasma contamination using the MycoAlert plus-color one-step mycoplasma detection kit (Yeasen, 40612ES25) and confirmed negative.

### Animals

Male C57BL/6 mice (6 to 8 weeks old; 18 to 22 g; Beijing Vital River Laboratory Animal Technology Co. Ltd.) were used for tumor implantation. A total of 1 × 10^6^ Hepa1-6 cells in 25 μl of PBS (biosharp, BL302A) were injected into the left liver lobe (for orthotopic tumors), and 1 × 10^6^ Hepa1-6 cells in 100 μl of PBS were injected into the right lower flank (for subcutaneous tumors) under isoflurane anesthesia (RWD, R510-22-10). Subcutaneous tumors became palpable at day 5 postinoculation. Orthotopic liver tumors were monitored by 1.5-T pet MRI (GSMED, China) starting at day 7, with humane endpoints set at tumor diameter >10 mm.

### Chemical synthesis and characterization of SPDs

The synthesis routes of SPDa and SPDb were shown in figs. S2 and S3. SPDa [Ac-K(PpIX)-FF-DHLASLWWGTEL] was synthesized using the standard SPPS on Rink amine resin. Briefly, 9-fluorenyl methoxycarbonyl (Fmoc)–linker was first coupled to the resin in *N*,*N*-dimethylformamide (DMF; Taitan, 68-12-2) using O-benzotriazole-N,N,N′,N′-tetramethyl-uronium-hexaf (HBTU; 2 equiv; MACKLIN, H810969) and N,N-diisopropylethylamine (DIPEA; 3 equiv; MACKLIN, N766991) as the activation system. Subsequently, Fmoc-protected amino acids were sequentially coupled from the C terminus to the N terminus in the following order: Fmoc-Leu-OH, Fmoc-Glu(OtBu)-OH, Fmoc-Thr(tBu)-OH, Fmoc-Gly-OH, Fmoc-Trp(Boc)-OH, Fmoc-Trp(Boc)-OH, Fmoc-Leu-OH, Fmoc-Ser(tBu)-OH, Fmoc-Ala-OH, Fmoc-Leu-OH, Fmoc-His(Trt)-OH, Fmoc-Asp(OtBu)-OH, Fmoc-Phe-OH, Fmoc-Phe-OH, and Fmoc-Lys(Dde)-OH. All amino acid coupling reactions were carried out in DMF using *N*,*N*-diisopropylcarbodiimide (DIC; 3 equiv; Macklin, 693-13-0) and 1-hydroxybenzotriazole (HOBt; 3 equiv) as the coupling reagents. Removal of the Fmoc protecting group was achieved using 20% (v/v) piperidine in DMF after each coupling cycle. To prevent undesired side reactions involving the α-amino group of lysine, acetic anhydride (Ac_2_O) was used to cap the lysine α-amino group following its incorporation. The Dde protecting group on the lysine side chain was selectively removed using 5% (v/v) hydrazine hydrate (N_2_H_4_·H_2_O; Shanghai Lingfeng Chemical Reagents Co. Ltd., 10217-52-4) in DMF, after which PpIX (Macklin, P815570-1g) was conjugated to the exposed amino group of lysine of Compound 1 to afford the side chain–modified peptide. Upon completion of the synthesis, the peptide was cleaved from the resin and concomitantly deprotected by treatment with a cleavage cocktail of trifluoroacetic acid (TFA; Macklin, 76-05-1)/triethylsilane (TIS; Macklin, 6485-79-6)/H_2_O (95%/2.5%/2.5%, v/v/v) at room temperature for 1 hour, yielding the crude peptide. The crude product was purified by preparative reversed-phase high-performance liquid chromatography (RP-HPLC) on a C18 column, affording SPDa in its purified form.

For synthesis of SPDb [Ac-K(PpIX)-FF-AEEA-C(MAL-PEG_4_-DBCO)], Fmoc-linker was initially coupled to the resin in DMF using HBTU (2 equiv) and DIPEA (3 equiv) as the activation system. Subsequently, the following Fmoc-protected building blocks were sequentially coupled in the order listed: Fmoc-Cys(Trt)-OH, Fmoc-AEEA-OH, Fmoc-Phe-OH, Fmoc-Phe-OH, and Fmoc-Lys(Boc)-OH. All coupling reactions were performed in DMF using DIC (3 equiv) and HOBt (3 equiv; MACKLIN, H742556) as coupling reagents. Upon completion of chain assembly, the peptide was cleaved from the resin, yielding the crude peptide. The crude product was subsequently purified, affording the target peptide (Compound 2) in purified form. Compound 2 (1 equiv) and DBCO-PEG_4_-Maleimide (1 equiv; Aladdin, D340482-50 mg) were dissolved in a mixed solvent of acetonitrile (ACN; Aladdin, E106172) and water, after which a buffer solution (pH = 7.2) was added. The reaction mixture was stirred at room temperature for 2 hours. Reaction progress was monitored by liquid chromatography–mass spectrometry (LC-MS), which confirmed complete consumption of the starting materials. The reaction mixture was subsequently subjected directly to preparative reversed-phase chromatography, affording Compound 4 after purification. In addition, PpIX (1.2 equiv) was dissolved in 10 ml of DMF and cooled to 0°C. 1-ethyl-3-(3-dimethylaminopropyl)carbodiimide hydrochloride (EDCI; Aladdin, E106172) and HOBt were then added, and the reaction mixture was stirred for 20 min. Subsequently, Compound 4 (1 equiv) and *N*-methylmorpholine (NMM; Energy Chemical, M0113453) were added, and the reaction was allowed to proceed overnight at room temperature. Reaction progress was monitored by thin-layer chromatography (TLC) and LC-MS, which confirmed complete conversion. The solvent was removed under reduced pressure to afford the crude product, which was purified directly by preparative RP-HPLC, yielding SPDb.

SPDa, SPDb, and the major intermediates (Compounds) in the synthetic route were characterized by MALDI-TOF MS (Bruker, Ultraflextreme, Germany) and ESI-MS (Agilent, 7890B-5977A, USA). After the 5-mg peptide was dissolved in 500 μl of DMSO, its chemical structure was analyzed by ^1^H NMR spectroscopy (Bruker, AVANCE III 500M with Prodigy Platform, USA).

### Characterization of SPD1 nanoparticles and SPD2 nanoparticles

SPD1 and SPD2 monomers were dissolved in DMSO as precursor stock solution (10 mM) and assembled into spherical nanoparticles via rapid precipitation by mixing with deionized water. For SPD1, a 1:1 mixture of SPDa and SPDb was diluted in DMSO and mixed with water to achieve final water fractions ranging from 0 to 99% (v/v). SPD1 nanoparticle assembly was validated using UV-vis spectroscopy (Cary 60 UV-Vis, Agilent, USA) and fluorescence spectroscopy (Infinite E Plex, TECAN, Switzerland; λ_ex_ = 405 nm). Spectra were recorded across the water fraction gradient (0 to 99%, v/v).

Hydrodynamic diameter and zeta potentials of SPD1 nanoparticles and SPD2 nanoparticles (both 50 μM) were determined by DLS (Zetasizer Nano ZS90, Malvern, UK). CAC values were determined fluorometrically (F-4600, HITACHI, Japan) using pyrene (Macklin, 129-00-0) as a hydrophobic probe. SPD1 or SPD2 solutions (0.01, 0.05, 0.1, 0.5, 1, 5, 10, 20, 30, 40, and 50 μM) were incubated with 0.1 mM pyrene in acetone at 37°C for 2 hours. The fluorescence intensity ratio (*I*_1_/*I*_3_) of pyrene (λ_ex_ = 335 nm; *I*_1_ = 373 nm; *I*_3_ = 384 nm) was plotted against log (concentration), with the CAC defined as the inflection point of the sigmoidal curve.

Nanoparticle morphology was assessed by 120-kV TEM (Talos L120C, Thermo Fisher Scientific, USA). SPD1 or SPD2 solutions (50 μM) with or without GPC3 protein incubation were deposited onto 200-mesh copper grids (BZ21022a, Zhong Jing Ke Yi, Beijing, China) with holey carbon support films. Samples were air-dried, negatively stained with 2% (w/v) uranyl acetate (ACMEC, U25690) for 1 to 3 min, and air-dried before imaging.

### BLI assay

BLI measurements were performed on an Octet Red96e instrument (Sartorius) at 25°C. Biotinylated SPD1 was immobilized onto streptavidin (SA) biosensors in kinetics buffer (PBS). Sensors were loaded to a response of ~1.0 to 1.2 nm, followed by a baseline equilibration step in kinetics buffer.

For association measurements, SPD1-loaded sensors were immersed in wells containing serial dilutions of recombinant human GPC3 prepared in kinetics buffer. Dissociation was monitored by transferring sensors into buffer-only wells. Parallel reference sensors without peptide loading were included to correct for nonspecific binding and baseline drift. Raw binding curves were aligned, reference subtracted, and fitted to a 1:1 binding model using the Octet Data Analysis software (v12.0) to extract kinetic parameters.

### Verification of fibrillar transformation by CD and FTIR spectroscopy

To verify the fibrillar transformation of SPD1 and SPD2 nanoparticles upon interaction with human GPC3, the secondary structure changes, specifically the formation of β sheet, were analyzed using CD spectroscopy and FTIR spectroscopy (Bruker Vertex 70, Bruker, Germany).

SPD1 and SPD2 nanoparticles (50 μM) incubated in the presence or absence of human GPC3 recombinant protein (NovoProtein, C414) at molar concentration ratio of 1000:1 for 24 hours at room temperature, and then CD spectra of peptide nanoparticle solutions were acquired using a CD spectrometer (Chirascan, Applied Photophysics, UK). Briefly, spectra were recorded in the far-UV region (185 to 260 nm) using a 0.1-cm quartz cuvette at 25°C. Each spectrum represents the average of two scans, with a 1-nm bandwidth and 0.5 s per point. Spectra of the buffer alone were recorded and subtracted from sample spectra. Secondary-structure estimation from CD spectra was performed using the BESTSEL algorithm (https://bestsel.elte.hu/idp_classification.php), which provides high-accuracy deconvolution for peptides and proteins. Baseline-corrected far-UV spectra were uploaded to the server, and the fractional contents of α helix, β strand, turn, and disordered structures were calculated automatically.

For FTIR analysis, the peptide nanoparticle solutions were lyophilized, and then 1 to 2 mg of the mixture was finely ground with 200 mg of spectroscopic-grade KBr and pressed into a transparent pellet using a hydraulic press. The pellet was placed into a FTIR spectrometer (Bruker Vertex 70, Bruker, Germany) for measurement. Spectra were collected over the range of 4000 to 400 cm^−1^ with a resolution of 4 cm^−1^, accumulating 32 scans per spectrum. Background scans of a pure KBr pellet were collected and subtracted. The amide I band region (1690 to 1660 cm^−1^) was analyzed for β sheet.

### MD simulation and molecular docking

For MD simulations of SPD1 self-assembly, three nonstandard residues were defined for SPD1: PpIX-K (denoted as KPP) in both SPDa and SPDb, AEEA (denoted as AEE) in SPDb, and Cys(NH_2_)-MAL-PEG_4_-DBCO (denoted as DBC) in SPDb. Geometry optimization and frequency calculations of these nonstandard residues were carried out at the B3LYP/6-31G(d) level using Gaussian (version 16; https://gaussian.com/gaussian16/), with solvent effects modeled by the solvation model based on density (SMD) in water. Restrained electrostatic potential (RESP) charges were derived using Multiwfn, and the corresponding topology and parameter files were generated with Sobtop (version 1.0; http://sobereva.com/soft/Sobtop). MD simulations were performed using GROMACS (version 2022.4; https://www.gromacs.org/) with the AMBER14SB force field, the TIP3P water model, and Na^+^ ions for charge neutralization. A cubic simulation box (12 nm by 12 nm by 12 nm) was constructed containing eight SPD1 molecules. The system was energy minimized in two steps (10,000 steps of steepest descent followed by 5000 steps of conjugate gradient), equilibrated with 500 ps of NVT (constant number of particles, volume, and temperature ensemble) and 500 ps of NPT (constant number of particles, pressure, and temperature ensemble) simulations, and subjected to 100 ns of production MD. The temperature was maintained at 298.15 K using the V-rescale thermostat, and the pressure was maintained at 1 bar using the Parrinello-Rahman barostat. Electrostatic interactions were treated with the particle mesh Ewald (PME) method, with real-space and van der Waals cutoffs of 1.2 nm. All hydrogen bonds were constrained with the LINCS algorithm.

For molecular docking of SPD1 with GPC3, the modeling of the SPD1 followed the same protocol as above. SPD1 was solvated in a cubic box (9.2 nm by 9.2 nm by 9.2 nm) and simulated with GROMACS using the AMBER14SB force field and TIP3P water. The system underwent two-step minimization (10,000 steps of steepest descent followed by 5000 steps of conjugate gradient), 500-ps NVT and 500-ps NPT equilibration, followed by a 50-ns production MD run. The SPD1 structure at 50 ns was selected for subsequent docking. The GPC3 protein structure was obtained from the PDB database (PDB ID: 7ZA1). Ligands and ions were removed using PyMOL (http://pymol.org/pymol), and AMBER14SB parameters were assigned using Chimera 1.16. Docking was performed with AutoDock Tools (version 1.5.6; http://autodock.scripps.edu/) for preprocessing. The docking grid box was centered at (17.960, 33.923, 18.156), with grid dimensions of 126 Å by 126 Å by 126 Å. Twenty docking runs were conducted using AutoDock Vina (version 1.2.5; https://vina.scripps.edu/).

For MD simulation of SPD1 nanoparticle with GPC3 protein, the SPD1 assembly structure obtained at 100 ns was combined with GPC3 protein. The GPC3 protein and SPD1 nanoparticle were randomly placed into a rectangular solvent box (17 nm by 25 nm by 17 nm). MD simulations were carried out in GROMACS using the AMBER14SB force field, TIP3P water, and Na^+^ ions. Following the same minimization and equilibration protocols as above, a 250-ns production simulation was performed. The temperature was controlled at 298.15 K with the V-rescale thermostat, the pressure at 1 bar was controlled with the Parrinello-Rahman barostat, and the integration step was 2 fs. PME was used for long-range electrostatics with a real-space cutoff of 1.2 nm; van der Waals interactions used the same cutoff. All hydrogen bonds were constrained with the LINCS algorithm.

### Validation of fibrillar transformation-induced redistribution of Gd elements using GPC3-modified gold nanoparticles

To investigate whether the fibrillar transformation of peptide nanoparticles alters the spatial distribution of metal elements, gold (Au) nanoparticles (5 nm in diameter) were functionalized with GPC3 protein using a commercial gold-conjugated protein labeling kit (R-SJH-0011, Ruixibio, China) following the manufacturer’s instructions. The GPC3-modified Au nanoparticles were subsequently mixed with either SPD1 or SPD2 nanoparticles (50 μM) with a weight ratio of 1:100. The mixture was gently agitated and incubated at 4°C for 24 hours to allow sufficient interaction and fibril formation. Following incubation, samples were prepared for TEM analysis by depositing 5 μl of the nanoparticle solution onto 200-mesh carbon-coated copper grids and allowing the grids to air-dry at room temperature. No negative staining was applied to directly visualize the distribution and localization of the GPC3-modified Au nanoparticles via a 120-kV TEM microscope.

### MRI scanning parameters for in vitro experiments

In vitro MRI measurements were performed using a 3.0-T clinical MRI scanner (uMR880, United Imaging, Shanghai, China) with a flexible joint coil compatible with small sample imaging. *T*_1_ mapping images were acquired using a spoiled gradient echo (SPGR) sequence with the following parameters: repetition time (TR) = 6.59 ms, echo time (TE) = 2.57 ms, number of flip angles = 4, matrix = 240 by 240, field of view (FOV) = 160 mm by 160 mm, spatial resolution = 0.67 mm by 0.67 mm by 3.5 mm, slice thickness = 3.5 mm, number of slices = 8, and number of signal averages (NSA) = 4. All MRI datasets were reconstructed and analyzed using medical image processing software (version R006, United Imaging, Shanghai, China).

### Measurement of field-dependent magnetization hysteresis loops

To evaluate field-dependent magnetization behavior, recombinant GPC3 protein was first incubated with SPD1 at a molar ratio of 1:1000 in PBS at room temperature for 24 hours. Subsequently, Gd-DOTA-N_3_ was added to the mixture and further incubated for 6 hours under the same conditions. The final liquid samples were carefully applied onto a 4-mm^2^ silicon substrate and dried at room temperature. The dried samples were mounted onto a quartz sample holder and subjected to magnetization hysteresis loop measurements using a vibrating sample magnetometer (model 8604, LakeShore, USA). The instrument features a pole diameter of 5 cm and a room temperature measurement sensitivity of 5 × 10^−7^ emu. Hysteresis loops were acquired under automated program control to ensure consistency and eliminate manual interference. The raw magnetization data (in emu) were normalized to sample mass and further converted to specific magnetization values (emu/g).

### SEM mapping

To assess the binding of Gd-DOTA-N_3_ to the SPD1/GPC3 fiber structure, SPD1 was first incubated with recombinant GPC3 protein at a molar ratio of 1:1000 in PBS for 24 hours to allow fiber formation. Gd-DOTA-N_3_ was then added and further incubated at room temperature for 6 hours. The mixture was lyophilized and mounted on conductive carbon tape for analysis. Environmental SEM (QUANTA 200, FEI, USA) equipped with EDX spectroscopy was used to analyze surface morphology and elemental distribution. Samples were imaged under low-vacuum mode (~1.0 torr) with a 15- to 20-kV accelerating voltage. Elemental mapping was performed for Gd, C, N, and O, and colocalization analysis confirmed the integration of Gd into the fiber structure.

### Western blot

The expression levels of GPC3 protein in HepG2, WRL-68, and Hepa1-6 cells were evaluated by Western blot. Briefly, cells were harvested and washed with PBS, followed by lysis on ice using radioimmunoprecipitation assay (RIPA) lysis buffer (EpiZyme, PC101) supplemented with 1% protease inhibitor cocktail (YEASEN, 20124ES03). The lysates were centrifuged at 12,000*g* for 3 min at 4°C, and the supernatants were collected. Total protein concentrations were determined using a bicinchoninic acid (BCA) protein assay kit (EpiZyme, ZJ101L). For electrophoresis, the remaining cell lysates were mixed with SDS–polyacrylamide gel electrophoresis (PAGE) loading buffer (Beyotime, P0287-10 ml) and heated at 100°C for 10 min. Proteins were separated by 10% SDS-PAGE and transferred onto polyvinylidene difluoride (PVDF) membranes. The membranes were blocked with 5% (w/v) nonfat dry milk (EpiZyme, PS112L) in Tris-buffered saline with 0.1% Tween 20 (TBST; EpiZyme, PS103S) for 1 hour at room temperature and incubated overnight at 4°C with a primary anti-GPC3 antibody (1:1000; Santa Cruz Biotechnology, sc-390587). After washing with TBST, the membranes were incubated with a horseradish peroxidase (HRP)–conjugated goat anti-mouse secondary antibody (1:10,000; Abbkine, A21010) for 1 hour at room temperature. Protein bands were visualized using chemiluminescence detection on an imaging system (AniView SE, BLT, China). Band intensities were quantified using ImageJ software (NIH; https://imagej.net/ij/).

### Flow cytometry assay

To assess GPC3 expression levels, cells (5 × 10^5^ cells per well, 12-well plates) were harvested using Accutase cell detach medium (Thermo Fisher Scientific, 00-4555-56), washed with staining buffer (PBS and 3% FBS), and fixed/permeabilized with 0.5 ml of 1× Fixation/Permeabilization solution (BD, 554714) for 45 min at room temperature in the dark. After washing with Perm/Wash buffer (BD, 554714), cells were incubated with anti-GPC3 antibody (Novus, NBP2-47760V; 1 to 2 μl per 10^6^ cells) in 100 μl of Perm/Wash buffer at 4°C for 30 min. Following additional washes, cells were analyzed using a fluorescence-activated cell sorting (FACS) cytometer (BD Biosciences, USA).

### IF detection of GPC3 expression

GPC3 expression in HepG2, WRL-68, and Hepa1-6 cell lines was evaluated by an IF staining assay. Cells (1.5 × 10^5^ per well) were seeded in a 4-well confocal dish and cultured overnight. After fixation with 4% paraformaldehyde (PFA; Biosharp, BL539A) for 20 min at room temperature, cells were permeabilized with 0.1% Triton X-100 (Biosharp, BL934B) for 5 min and washed twice with PBS. Blocking was performed with 10% bovine serum albumin (BSA; A500023-0025, Sangon Biotech, China) in PBS for 1 hour at room temperature with gentle shaking. Cells were then incubated overnight at 4°C with FITC-conjugated anti-GPC3 antibody (Novus, NBP2-47760V; 1:200 in 10% BSA/PBS). The following day, cells were washed three times with PBS, stained with 4′,6-diamidino-2-phenylindole (DAPI; 1:1000; Sigma-Aldrich, D9542-1MG) for 2 to 3 min, and washed again. Images were acquired using a confocal laser scanning microscope (LSM 980, Carl Zeiss, Germany) with a 10× objective lens.

### Cellular distribution and endocytic mechanism of SPD1 and SPD2 nanoparticles

SPD1 and SPD2 nanoparticles were prepared via a rapid nanoprecipitation method by mixing the precursor stock solution with complete DMEM to yield a final concentration of 50 μM. Cells (2 × 10^4^ cells per well) were seeded in 4-well confocal microscopy dishes and cultured overnight at 37°C in a humidified atmosphere containing 5% CO_2_. The culture medium was then replaced with SPD1 or SPD2 nanoparticle suspensions, and cells were incubated for 6 hours under standard conditions. After incubation, cells were fixed with 4% PFA for 20 min at room temperature and subsequently washed three washes with PBS for 5 min each. Nuclei staining was performed using DAPI for 3 min at room temperature, followed by three additional PBS washes. Cellular localization of nanoparticles was visualized using a confocal laser scanning microscope equipped with a 63× oil-immersion objective lens.

To investigate the endocytic pathways involved in nanoparticle uptake, cells were pretreated with specific inhibitors before nanoparticle exposure: 5 mM β-CD (Macklin, C6289-25g) incubation for 1 hour to inhibit caveolae-mediated endocytosis, 2 mM amiloride (Yuanye, S82129-5mg) incubation for 6 hours to block macropinocytosis, and 450 mM hypertonic sucrose (Macklin, S818046-500g) incubation for 30 min to suppress clathrin-mediated endocytosis. After pretreatment, cells were incubated with SPD1 or SPD2 nanoparticles as described above and analyzed via CLSM. A 405-nm laser was used to excite both PpIX and DAPI, and corresponding red and blue fluorescence signals were acquired.

### Colocalization of SPD1 nanoparticles and GPC3 on the cell membrane

To assess the spatial colocalization of SPD1 nanoparticles and GPC3 on the cell membrane, IF staining was performed, followed by CLSM analysis. HepG2 cells were seeded onto glass-bottom confocal dishes and incubated with SPD1 nanoparticles (50 μM) for 6 hours at 37°C. After incubation, cells were washed with PBS and fixed in 4% PFA overnight at 4°C. Fixed cells were then blocked with 5% (w/v) nonfat dry milk in PBS for 1 hour at room temperature to prevent nonspecific binding. Cells were subsequently incubated with a primary anti-GPC3 antibody (1:200; Santa Cruz Biotechnology, sc-390587) overnight at 4°C. After three PBS washes (5 min each), cells were incubated with an FITC-conjugated goat anti-mouse IgG secondary antibody (1:1000; Abbkine, A23210) for 1 hour at room temperature in the dark. Nuclei were counterstained with DAPI for 3 min, followed by PBS washing. Fluorescence images were acquired using CLSM. Colocalization analysis between SPD1 nanoparticles and GPC3 signals was performed using MATLAB software (version 9.6.0.1072779, MATLAB, MathWorks, USA; https://mathworks.com).

### GPC3-induced fibrillar transformation of SPD1 nanoparticles on the cell membrane

To investigate the GPC3-induced fibrillar transformation of SPD1 nanoparticles on the cell membrane, both SEM and TEM were used. For SEM analysis, cells were seeded onto glass-bottom dishes and incubated with SPD1 nanoparticles (50 μM) for 6 hours at 37°C. Following incubation, cells were washed with PBS and fixed with 2.5% glutaraldehyde (Macklin, BL539A) overnight at 4°C. Fixed samples were subsequently dehydrated through a graded ethanol series (30, 50, 70, 80, 90, and 99.5%; Macklin, E821483) for 5 min at each concentration. After dehydration, samples were immersed three times in hexamethyldisilazane (Macklin, H810965) for 1 min each and then air-dried in a desiccator. Dried specimens were mounted onto SEM stubs using double-sided conductive tape, sputter coated with gold (15 mA, 60 s), and imaged by field-emission SEM (SU8100, HITACHI, Japan). For ultrastructural observation via TEM, cells were fixed with 2.5% glutaraldehyde (Macklin, BL539A) and postfixed with 1% osmium tetroxide (Polysciences, 23310-10). Following PBS washes, samples were dehydrated through an ethanol gradient and embedded in epoxy resin. Ultrathin sections (~70 nm) were cut using an ultramicrotome, stained with uranyl acetate and lead citrate, and imaged by TEM.

To evaluate the persistence of SPD1-derived nanofiber networks on the cell membrane, cells were incubated with SPD1 nanoparticles (50 μM) for 6 hours. The supernatants containing unbound nanoparticles were removed, and cells were replenished with fresh complete DMEM. After 24 or 72 hours of continued culture, cells were fixed and imaged by SEM as described above. Parallel samples were also analyzed by CLSM to assess membrane-associated nanofiber distribution.

### Cell-surface validation and kinetic characterization of bioorthogonal conjugation in HepG2 cells

To verify the occurrence and characterize the kinetics of the bioorthogonal reaction on the surface of hepatocellular carcinoma cells, both CLSM and ICP-MS were used. HepG2 cells were seeded in 4-well confocal dishes at a density of 2 × 10^4^ cells per well and incubated overnight under standard conditions. For membrane-anchored peptide pretargeting, cells were treated with 50 μM SPD1 for 6 hours. After thorough washing with PBS to remove unbound peptides, the cells were incubated with varying concentrations (10, 20, 40, and 50 μM) of FITC-N_3_ at for an additional 6 hours to initiate the bioorthogonal click reaction. Cells were subsequently fixed with 4% PFA for 20 min at room temperature and stained with DAPI for nuclear visualization. CLSM was used to capture the red fluorescence from SPD1 and the green fluorescence from FITC-N_3_. Colocalization signals (yellow fluorescence) were interpreted as evidence of a bioorthogonal reaction on the cell membrane.

To quantitatively evaluate the extent and kinetics of the bioorthogonal reaction, ICP-MS was used to measure Gd levels after incubation with SPD1 and Gd-DOTA-N_3_. HepG2 cells were pretreated with 50 μM SPD1 or SPD2 nanoparticles for 6 hours, followed by incubation with 50 μM Gd-DOTA-N_3_ for various durations (0.5, 1, 6, and 12 hours). After each time point, cells were washed thoroughly with PBS to remove unreacted or noninternalized Gd-DOTA-N_3_. Cells were then lysed using RIPA buffer containing 1% protease inhibitor cocktail, and the lysates were collected for quantitative analysis. Gd content analysis was measured by ICP-MS (Agilent 7700, Agilent Technologies Inc., Santa Clara, CA, USA). The Gd concentration was normalized to total protein content (measured by BCA assay). Gd content in cell lysates was determined by ICP-MS (Agilent 7700, Agilent Technologies Inc., Santa Clara, CA, USA) and normalized to the total protein concentration measured by BCA assay as described above. The Gd-DOTA-N_3_ mass per microgram of protein was calculated on the basis of the molecular weight of the conjugate. The absolute amount of bioorthogonally reacted Gd-DOTA-N_3_ was derived by subtracting the nonspecific accumulation (Gd-DOTA-N_3_ group) from the total Gd detected in the SPD1- or SPD2-treated groups. Bioorthogonal reaction efficiency (%) was calculated by the formula: Reaction efficiency (%) = (Mass of reacted Gd-DOTA-N_3_/Total input mass of Gd-DOTA-N_3_) × 100%.

### Cytotoxicity of SPD1 and SPD2 nanoparticles

The in vitro cytotoxicity of SPD1+Gd-DOTA-N_3_, SPD2+Gd-DOTA-N_3_, Gd-DOTA-N_3_, and Gd-DOTA was evaluated using the Cell Counting kit-8 (CCK-8; Abbine, BS350B) colorimetric assay. Cells were seeded in 96-well plates at a density of 6 × 10^3^ cells per well and cultured overnight in DMEM complete medium under standard conditions. The following day, the culture medium was replaced with fresh DMEM containing SPD1 or SPD2 nanoparticles at final concentrations of 1, 10, 50, 100, or 200 μM and incubated for 6 hours. Cells were then exposed either to 50 μM Gd-DOTA-N_3_ for a further 6 hours or directly to 1 to 100 μM Gd-DOTA-N_3_. After 24 hours of incubation, 10 μl of CCK-8 solution was added to each well, and the plates were incubated for an additional 2 to 4 hours. Absorbance was measured at 450 nm using a multiwavelength microplate reader (Infinite E Plex, TECAN, Switzerland). Wells without cells were used as blanks, and wells with untreated cells served as controls. Cell viability was calculated according to the following formula: Cell viability (%) = [(OD 450 treated − OD 450 blank)/(OD 450 control − OD 450 blank)] × 100%.

### Plasma pharmacokinetics of peptide nanoparticles and Gd ions

To evaluate the plasma pharmacokinetics of peptide nanoparticles, SPD1 or SPD2 (10 mg/kg) was administered via intravenous injection into the tail vein of healthy C57BL/6 mice (*n* = 3 per time point; total of 30 mice per group). At predetermined time points (5, 10, 20, and 30 min and 1, 2, 4, 6, 12, and 24 hours), 20 μl of blood was collected from the tail vein and diluted 1:10 in PBS. The samples were centrifuged at 3000*g* for 5 min at 4°C, and the supernatants were collected for analysis. The concentration of PpIX from SPD1 was quantified using a fluorescence spectrophotometer (excitation: 405 nm; emission: 640 nm). A standard fluorescence-concentration calibration curve was constructed, and the plasma concentration-time profiles were fitted using noncompartmental analysis with PKSolver (v2.0, China Pharmaceutical University, Nanjing, China). The pharmacokinetic parameters, including AUC_0–24_, *t*_1/2_, *C*_max_, *V*_d_, and CL, were calculated accordingly.

To investigate the pharmacokinetics of Gd^3+^-based contrast agents after nanoparticle treatment, C57BL/6 mice bearing Hepa1-6 liver tumors were intravenously injected with SPD1 or SPD2 (10 mg/kg). Six hours postinjection, Gd-DOTA or Gd-DOTA-N_3_ was intravenously administered at a dose of 0.1 mmol/kg. Blood samples (20 μl each) were collected at 10, 20, 30, and 40 min and at 1, 2, 4, 6, 12, and 24 hours postinjection. Each blood sample was mixed with 180 μl of heparinized PBS and centrifuged at 3000*g* for 5 min at 4°C to obtain the plasma. Gd concentration in plasma was determined via ICP-MS, and a standard curve was established for quantification. Noncompartmental pharmacokinetic modeling was performed, and parameters including AUC_0–24_, *t*_1/2_, *C*_max_, *V*_d_, and CL were calculated using the same analysis software.

### Biodistribution of peptide nanoparticles

The biodistribution of SPD1 and SPD2 nanoparticles was evaluated using ex vivo fluorescence imaging. An orthotopic Hepa1-6 hepatocellular carcinoma model was established in C57BL/6 mice. When the tumor volume reached ~100 mm^3^, the mice were randomly assigned into two groups (*n* = 3 per time point per group; total *n* = 48) and intravenously administered a single dose of SPD1 or SPD2 nanoparticles (10 mg/kg) via the tail vein. At predetermined time points postinjection (0.5, 1, 2, 4, 6, 9, 12, 24, and 72 hours), mice were anesthetized using isoflurane and euthanized by cervical dislocation. Orthotopic liver tumors and major organs (brain, liver, heart, spleen, lungs, kidneys, and intestines) were harvested and immediately imaged using a small animal fluorescence imaging system (AniView SE, BLT, China). Fluorescence intensity was recorded and analyzed to assess the time-dependent tissue distribution profiles of SPD1 and SPD2 nanoparticles.

### MRI scanning parameters in vivo

MRI of subcutaneous liver tumor-bearing mice was performed under isoflurane anesthesia using a 1.5-T preclinical MRI system (GSMED, China) equipped with a dedicated mouse radio frequency coil. Mice were positioned prone with a tail-first orientation, and axial scans were acquired to encompass the full tumor volume. *T*_1_-weighted spin echo (*T*_1__SE) sequences were obtained with the following parameters: TE = 18 ms, TR = 400 ms, slice thickness = 3 mm, slice gap = 1 mm, number of slices = 15, NAS = 1, FOV = 18 cm by 18 cm, and spatial resolution = 0.469 mm by 0.469 mm by 3 mm.

MRI of orthotopic liver tumor-bearing mice was performed under isoflurane anesthesia using a 3.0-T clinical MRI system (Ingenia; Philips Healthcare) equipped with a dedicated mouse radio frequency coil. Mice were positioned supine to ensure stable respiratory motion and optimal liver coverage. Fat-suppressed *T*_1_-weighted fast spin-echo (*T*_1__FSE) sequences were acquired with the following parameters: TE = 12 ms, TR = 600 ms, slice thickness = 1 mm, slice gap = 0 mm, number of slices = 16, NAS = 4, FOV = 40 mm by 40 mm, and spatial resolution = 0.25 mm by 0.25 mm by 1 mm. Axial imaging was performed to encompass the entire hepatic region and capture the full extent of the orthotopic tumor.

### Hemolysis assay

Fresh whole blood was collected from the inner canthus of mice using anticoagulant tubes. Red blood cells (RBCs) were isolated by centrifugation of whole blood per replicate at 3000*g* for 10 min at 4°C. The supernatants were discarded, and the RBC pellets were washed two to three times with PBS until the supernatants became colorless. Washed RBCs were then resuspended in PBS, ddH_2_O, or the indicated test solutions. The following treatment groups were prepared: (i) ddH_2_O (positive control), (ii) PBS (negative control), (iii) Gd-DOTA (0.5 mg/ml), (iv) Gd-DOTA-N_3_ (0.5 mg/ml), (v) SPD1 (0.5 mg/ml)+Gd-DOTA-N_3_ (0.5 mg/ml), and (vi) SPD2 (0.5 mg/ml)+Gd-DOTA-N_3_ (0.5 mg/ml). All treatments were diluted in PBS. RBC suspensions were incubated with each formulation under standard conditions, and hemolysis was assessed by photographing and measuring the absorbance of supernatants at 570 nm using a spectrophotometer (Infinite E Plex, TECAN, Switzerland).

### Histopathological and biosafety evaluation

Orthotopic Hepa1-6–bearing C57BL/6 mice were randomly divided into four groups (*n* = 3 per group, total *n* = 12) for different treatment regimens: (i) intravenous injection of Gd-DOTA (200 μl per mouse); (ii) intravenous injection of Gd-DOTA-N_3_ (200 μl per mouse); (iii) intravenous injection of SPD1 nanoparticles (10 mg/kg) followed by Gd-DOTA-N_3_ (200 μl); and (iv) intravenous injection of SPD2 nanoparticles (10 mg/kg) followed by Gd-DOTA-N_3_ (200 μl). All treatments were administered as a single dose via the tail vein. After completion of MRI evaluation, mice were euthanized by isoflurane (RWD, R510-22-10) inhalation followed by cervical dislocation. Tumor tissues were harvested for histopathological and immunological analyses. H&E staining was performed to assess general tissue morphology. IHC staining was conducted to evaluate GPC3 expression using a primary anti-GPC3 antibody (1:200; Abcam, ab207080). Apoptotic cells were identified by TUNEL assay using a commercial kit (1:5:50; Servicebio, G1501-100T) with immunofluorescence detection. In addition to tumor tissues, major organs—including the heart, liver, spleen, lung, kidney, and small intestine—were harvested for H&E staining to evaluate potential systemic toxicity. Furthermore, whole blood samples were collected via cardiac puncture immediately postmortem for routine serum biochemical analyses to assess hepatic and renal function as part of the biosafety evaluation.

Serum concentrations of TNF-α, IL-1β, and IL-6 in the mouse tumor models were quantified using commercial ELISA kits specific for TNF-α (BIOESN, BES0211K), IL-1β (BIOESN, BES0032K), and IL-6 (BIOESN, BES0087K). Blood samples were collected by cardiac puncture, allowed to clot at room temperature, and centrifuged to obtain serum. Assays were performed in accordance with the manufacturer’s instructions. Absorbance was recorded at 450 nm using a microplate reader, and cytokine concentrations were calculated from standard curves.

### Quantitative biodistribution analysis by ICP-MS

To evaluate the biodistribution of Gd-based probes, mice were euthanized 1 hour post–Gd-DOTA-N_3_ injection. Tumor tissues and major organs including the heart, liver, spleen, lung, and kidney were collected and weighed. For sample preparation, tissues were homogenized in PBS at a ratio of 1 g of tissue per 1 ml of PBS using a mechanical homogenizer. Each homogenate sample was transferred to a Teflon digestion vessel, and concentrated nitric acid (70%) was added. The samples were digested at an elevated temperature (≥120°C) for at least 6 hours until complete dissolution of the tissues and evaporation of the liquid phase. After cooling to room temperature, the residual solids were reconstituted in 2% nitric acid (v/v, prepared using ddH_2_O) and filtered through 0.22-μm filters to remove particulates. Gd concentration in each sample was determined using ICP-MS.

### TEM of tumor tissues

Tumor tissues were collected, cut into ~1-mm^3^ pieces, and immediately fixed in 2.5% glutaraldehyde at 4°C overnight. Following primary fixation, samples were washed three times with PBS and postfixed in 1% osmium tetroxide for 1 hour at 4°C. The specimens were then dehydrated through a graded ethanol series (30, 50, 70, 80, 90, and 100%; 10 min each), followed by infiltration with propylene oxide and embedding in EPON 812 epoxy resin. After polymerization at 60°C for 48 hours, ultrathin sections (70 to 90 nm) were obtained using an ultramicrotome equipped with a diamond knife. Sections were mounted on copper grids and sequentially stained with 2% uranyl acetate for 10 min and lead citrate for 5 min. The samples were examined on a 120-kV TEM (Talos L120C, Thermo Fisher Scientific).

### Statistical analysis

All experiments were independently performed at least three times. Data were expressed as means ± SD (SD) or as median [interquartile range (IQR)] when appropriate. Statistical differences between groups were analyzed using independent two-sample *t* tests. One-way ANOVA or two-way ANOVA followed by Tukey’s post hoc analysis was used for multiple group comparison. All statistical analyses were conducted using GraphPad Prism (version 8, GraphPad Software, La Jolla, CA, USA; https://graphpad.com/). *P* < 0.05 was considered statistically significant.
