# Supplementary material for: In vivo membrane engineering traps Gd-based MRI contrast agents for detecting microhepatocellular carcinoma
Source: Sci Adv. 2026 Apr 29;12(18):eaec9913. doi: 10.1126/sciadv.aec9913 (PMC13127589; doi:10.1126/sciadv.aec9913)
Supplement: Supplementary file 1 — Figs. S1 to S46 Tables S1 and S2 [file sciadv.aec9913_sm.pdf]

Science Advances
AAAS

Supplementary Materials for

In vivo membrane engineering traps Gd-based MRI contrast agents for detecting microhepatocellular carcinoma

Chunping Mao *et al.*

Corresponding author: Jun Shen, shenjun@mail.sysu.edu.cn; Guobin Hong, honggb@smu.edu.cn; Lu Zhang, zhanglu@sustech.edu.cn

Sci. Adv. **12**, eaec9913 (2026)
DOI: 10.1126/sciadv.aec9913

This PDF file includes:

Figs. S1 to S46
Tables S1 and S2

SPDa, Ac-K(PpIX)-FF-DHLASLWWGTEL, $C_{127}H_{159}N_{25}O_{25}$, 2434.19 g/mol

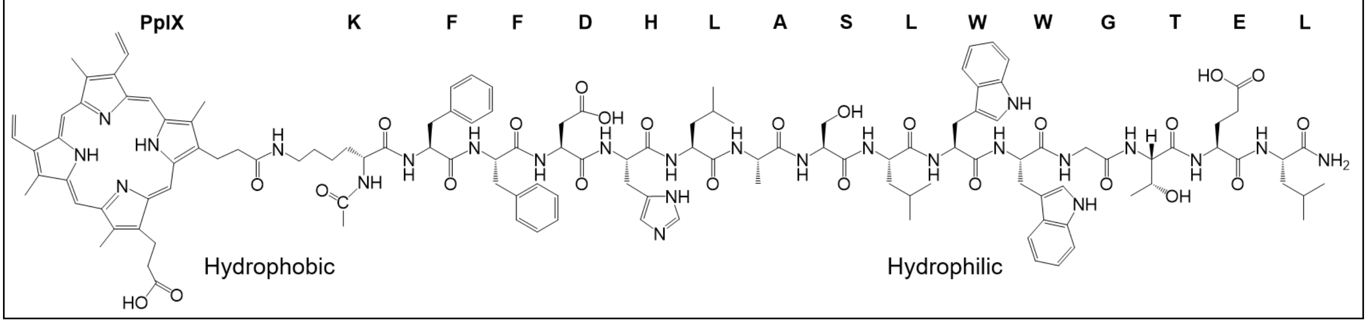

SPDb, Ac-K(PpIX)-FF-AEEA-C(DBCO-PEG$_4$-Maleimide), $C_{105}H_{125}N_{15}O_{20}S$, 1947.89 g/mol

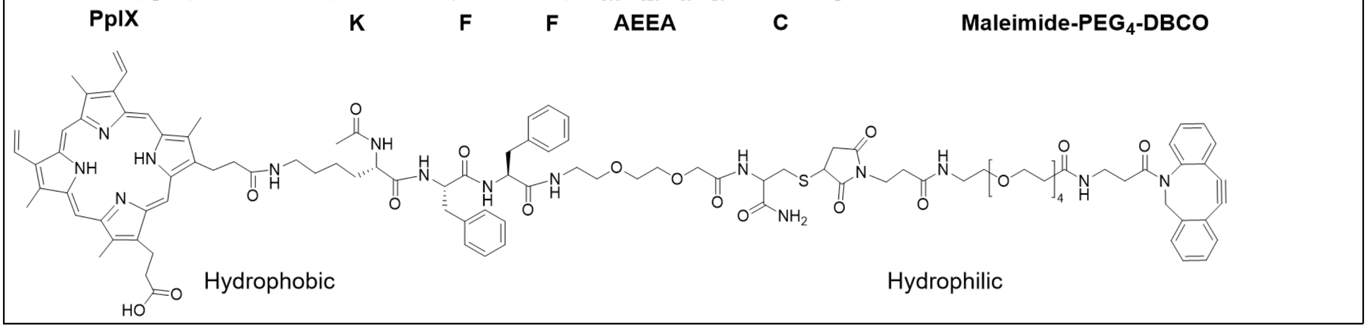

Fig. S1. Chemical structures of SPDa and SPDb. SPD1 is composed of an equimolar mixture of SPDa and SPDb. SPD2, consisting solely of SPDb, serves as a non-targeting control. Chemical structures were generated using Chemdraw® software (version 20.0.0.41; PerkinElmer Informatics, Boston, MA, USA).

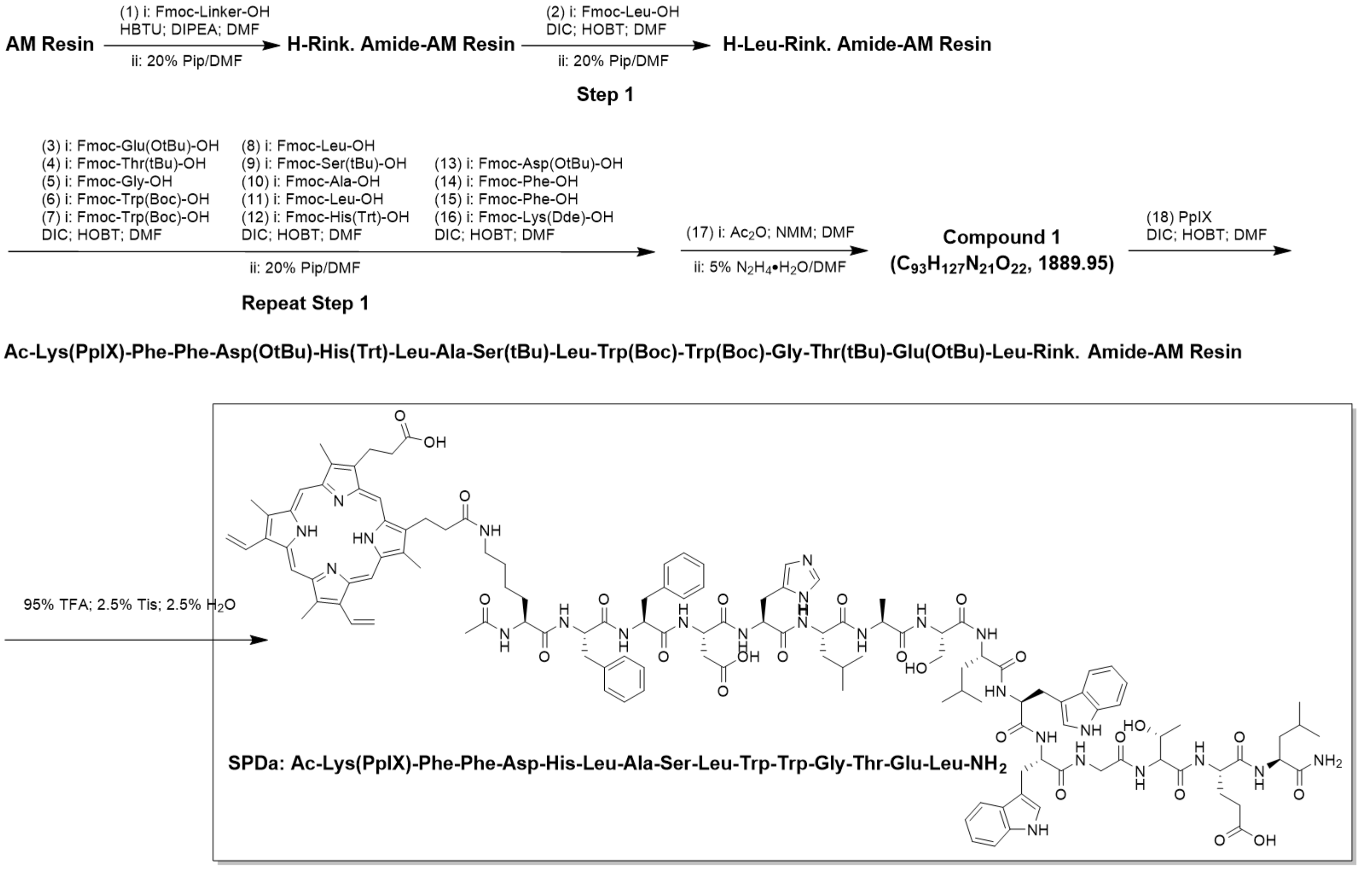

Fig. S2. Synthetic route of SPDa. Schematic illustration of the stepwise synthesis of SPDa via Fmoc solid-phase peptide synthesis on Rink Amide-AM resin, including iterative amino acid coupling, orthogonal side-chain protection and deprotection, on-resin conjugation of PpIX to the lysine residue, and final cleavage with global deprotection to yield SPDa.

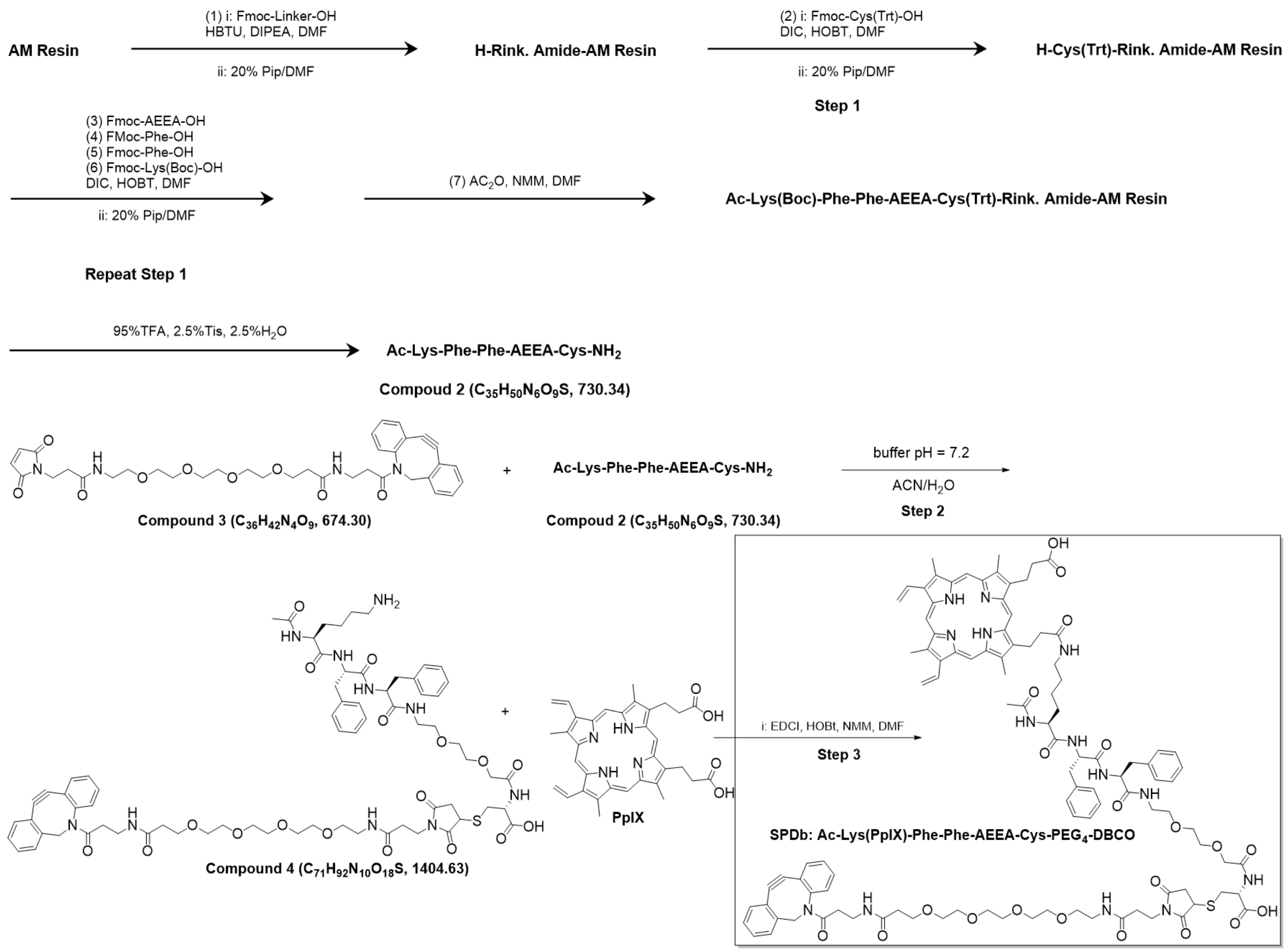

Fig. S3. Synthetic route of SPDb. Schematic illustration of the stepwise synthesis of SPDb, involving Fmoc solid-phase peptide synthesis on Rink Amide-AM resin to obtain the peptide precursor, followed by solution-phase functionalization, including addition reactions for linker and photosensitizer conjugation, and final coupling and purification to yield SPDb.

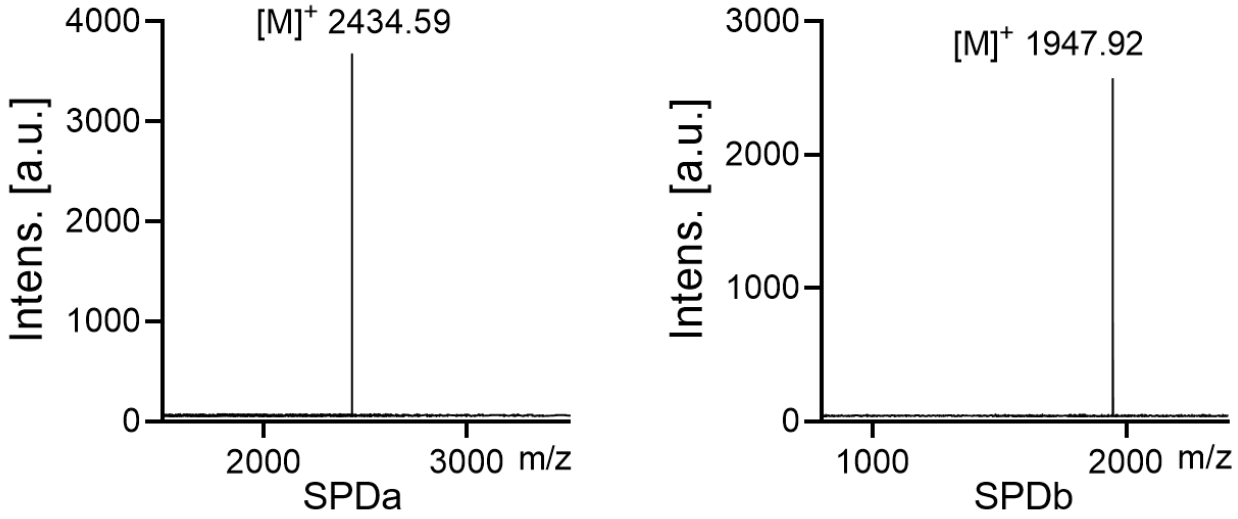

Fig. S4. MALDI-TOF MS characterization of SPDa and SPDb. Matrix-assisted laser desorption ionization time-of-flight mass spectrometry (MALDI-TOF MS) was used to verify the molecular weights of SPDa and SPDb. The observed mass peaks match the calculated values.

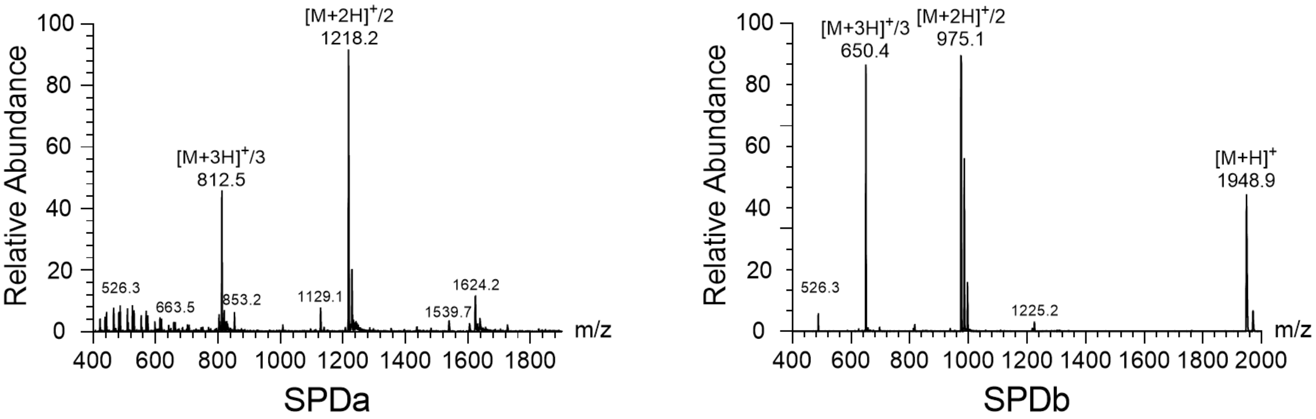

Fig. S5. ESI-MS of SPDa and SPDb. Electrospray ionization mass spectrometry (ESI-MS) was used to confirm the molecular weights of SPDa and SPDb. The observed mass spectra are in good agreement with the calculated values.

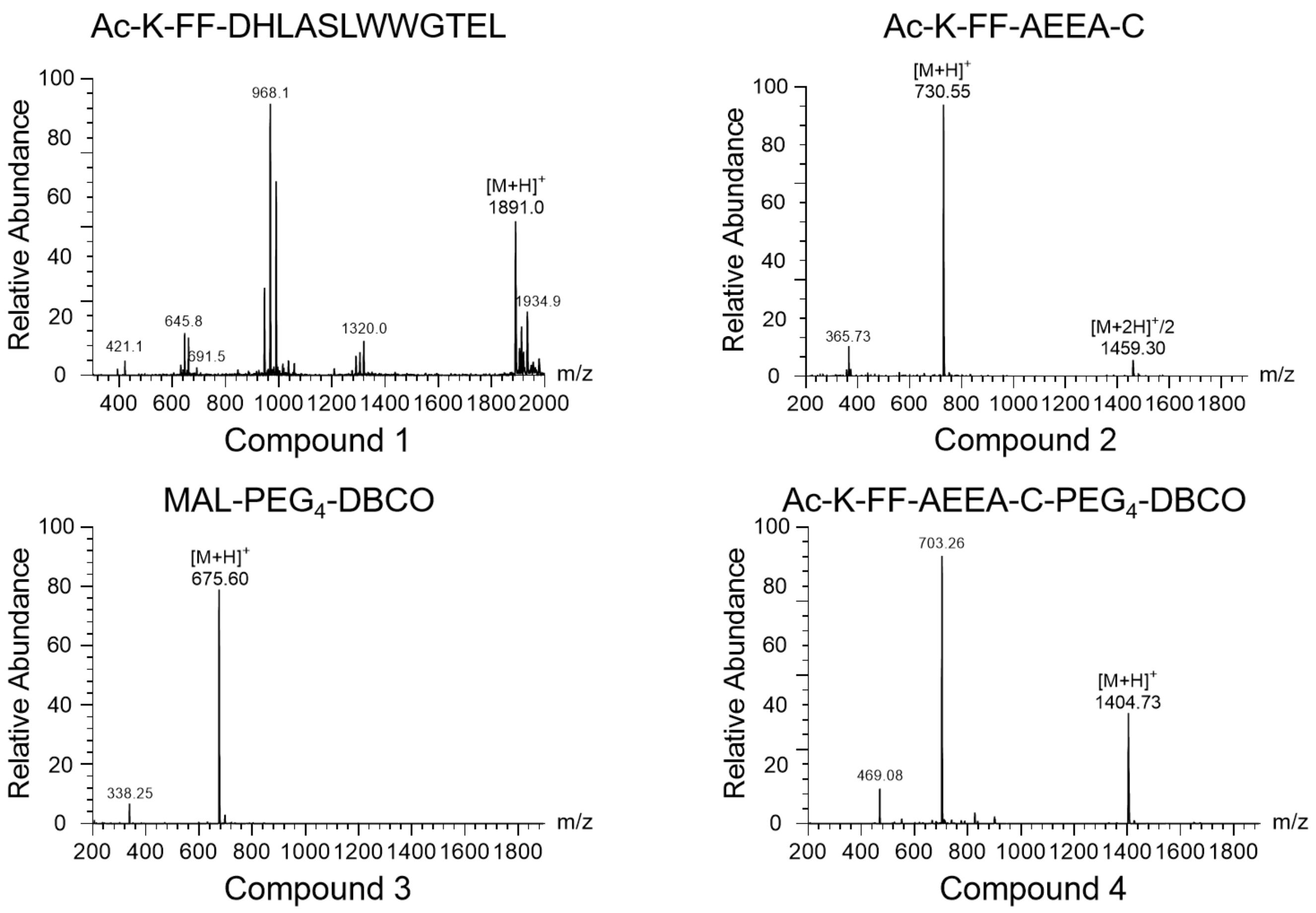

Fig. S6. ESI-MS characterization of key synthetic intermediates for SPDa and SPDb. Electrospray ionisation mass spectrometry (ESI-MS) was used to characterize the principal intermediates generated during the stepwise synthesis of SPDa and SPDb. The observed molecular ion peaks are consistent with the calculated molecular weights, confirming the successful formation of each intermediate.

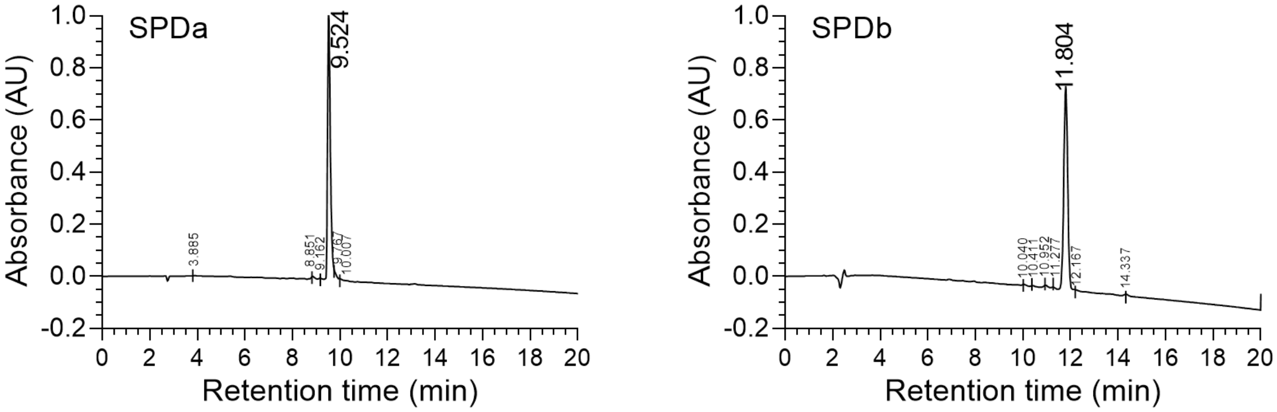

Fig. S7. Analytical HPLC characterization of SPDa and SPDb. Representative chromatograms of SPDa and SPDb, each exhibiting a dominant single peak with purities of 95.47% and 96.43%, respectively, confirming the successful synthesis and purification of both peptides.

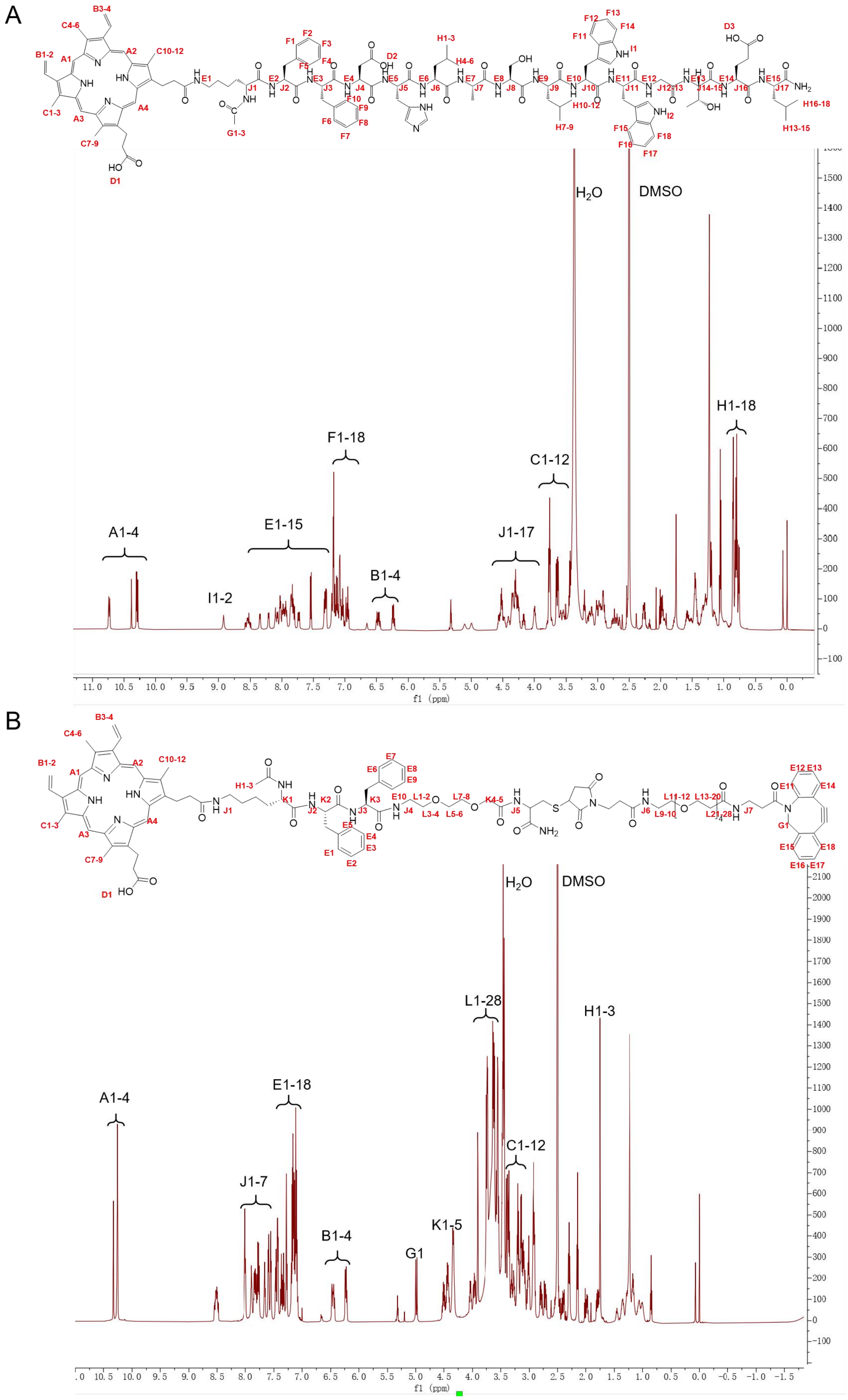

Fig. S8. ^{1}H NMR spectrum of SPDa and SPDb.

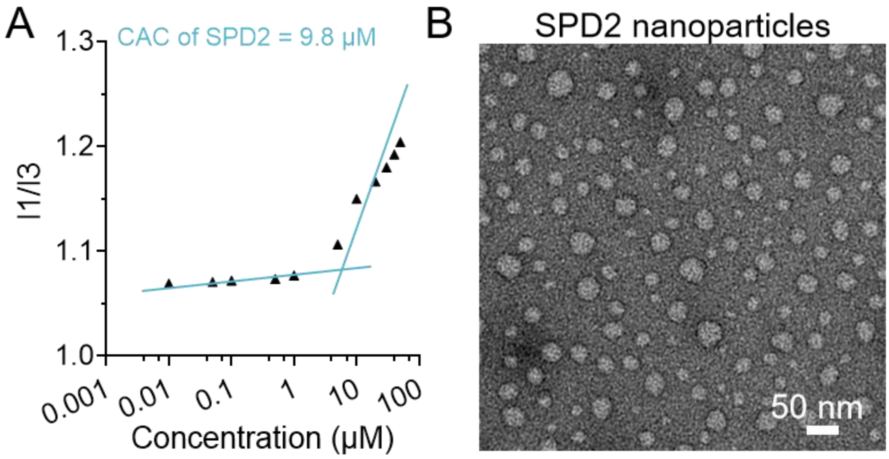

Fig. S9. In vitro self-assembly capability of SPD2. (A) The critical aggregation concentration (CAC) of SPD2 was measured using pyrene as a hydrophobicity-sensitive probe. (B) The morphology of assembled SPD2 structures (50 μM) was assessed by transmission electron microscopy (TEM). Experiments were independently repeated three times, with consistent results observed across replicates.

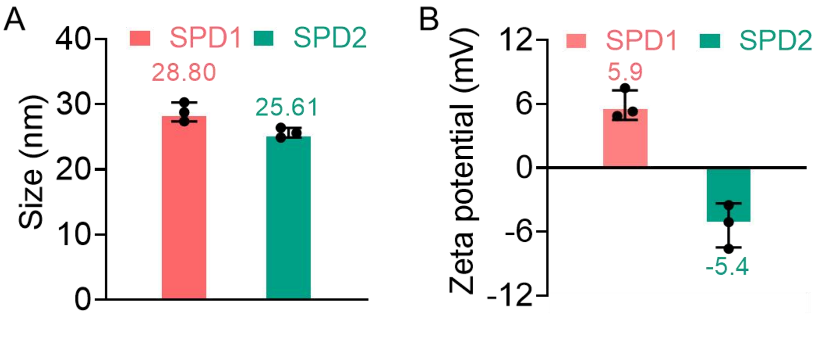

Fig. S10. Size distribution and surface charge characteristics of SPD1 and SPD2 nanoparticles. (A) Hydrodynamic diameters of SPD1 nanoparticles (50 μM) and SPD2 nanoparticles (50 μM) were measured by dynamic light scattering (DLS). (B) Zeta potential measurements of freshly prepared SPD1 and SPD2 nanoparticles (50 μM). All data are presented as mean ± SD from three independent experiments (n = 3).

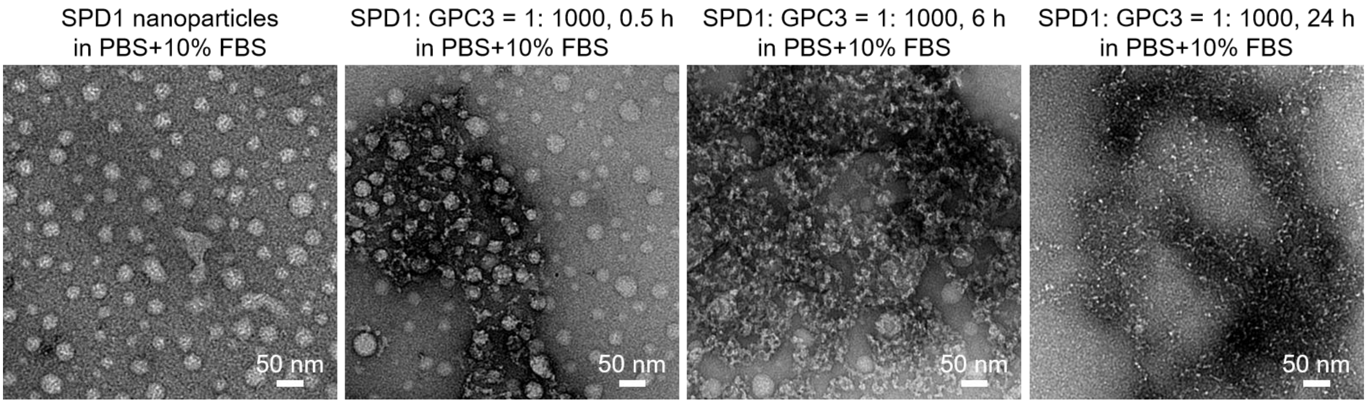

Fig. S11. Time-dependent fibrillar transformation of SPD1 nanoparticles induced by human GPC3 protein in a physiological-mimicking environment. TEM images showing the morphology of the initial SPD1 nanoparticles (50 μM) and the nanofibers formed after incubating SPD1 nanoparticles (50 μM) with human GPC3 protein (MW ≈ 61.6 kDa) in a physiological-mimicking environment (PBS supplemented with 10% FBS) for the indicated time points. The molar ratio of human GPC3 protein to SPD1 was approximately 1:1,000.

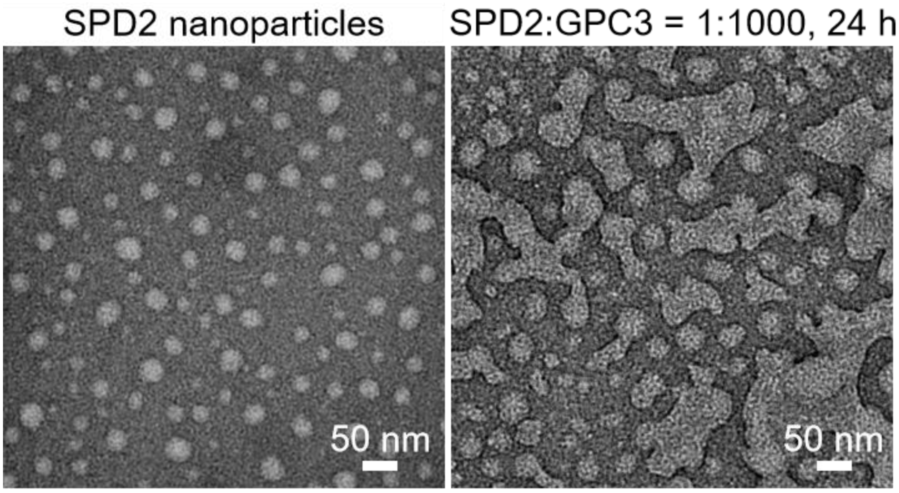

Fig. S12. TEM characterization of SPD2 nanoparticles before and after incubation with GPC3 protein. TEM images show the morphology of freshly prepared SPD2 nanoparticles and SPD2 nanoparticles after incubation with GPC3 protein (MW ≈ 61.6 kDa) for 24 h. The molar ratio of GPC3 protein to SPD2 nanoparticles was approximately 1:1,000. The concentration of SPD2 nanoparticles used in this experiment was 50 μM. Representative images from three independent experiments are shown.

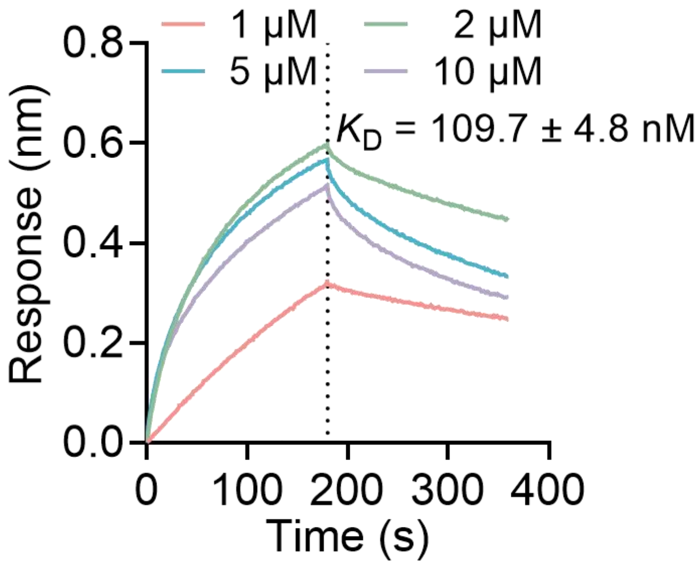

Fig. S13. Kinetic analysis of SPD1 binding to recombinant human GPC3 protein measured by BLI. Biolayer interferometry (BLI) sensorgrams showing the binding responses of SPD1 to immobilized recombinant human GPC3 (MW ≈ 61.6 kDa) at indicated concentrations (1, 2, 5, and 10 μM). The equilibrium dissociation constant (K_D) was determined to be 109.7 ± 4.8 nM.

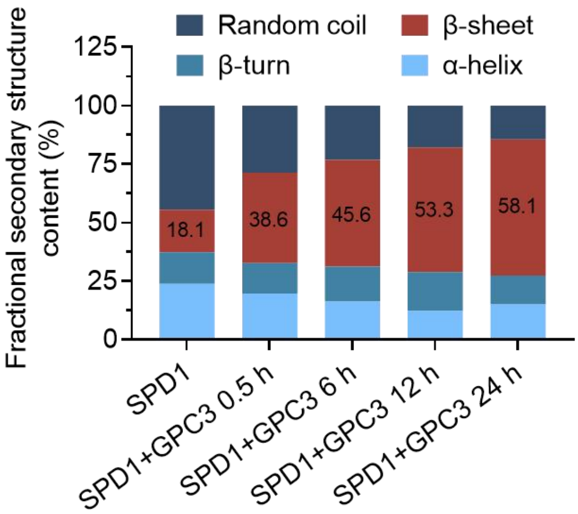

Fig. S14. Time-dependent evolution of the secondary structure of SPD1 upon interaction with GPC3. Stacked bar charts display the relative proportions of random coil, β-turn, β-sheet, and α-helix structures. The molar ratio of GPC3 protein to SPD1 nanoparticles was approximately 1:1,000. The concentration of SPD1 nanoparticles used in this experiment was 50 μM. Error bars are omitted because CD-based quantification reports proportional secondary-structure compositions rather than independent measurements.

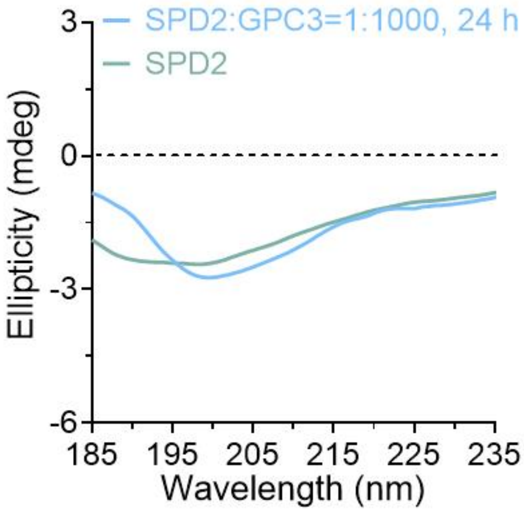

Fig. S15. Circular dichroism (CD) spectra of initial SPD2 nanoparticles and SPD2 nanoparticles after incubation with GPC3 protein. CD spectra were measured for initial SPD2 nanoparticles and SPD2 nanoparticles incubated with GPC3 protein (MW ≈ 61.6 kDa) at a GPC3 protein-to-SPD2 nanoparticles molar ratio of 1:1,000 for 24 h. The concentration of SPD2 nanoparticles was 50 μM. Experiments were independently repeated three times, with consistent results observed across replicates. mdeg, millidegrees.

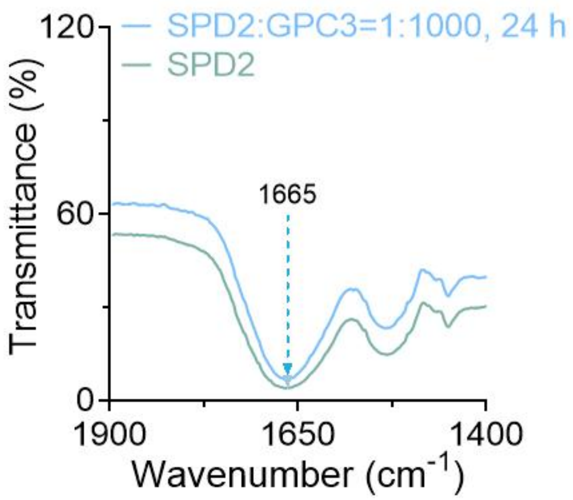

Fig. S16. FTIR spectra of SPD2 nanoparticles before and after interaction with GPC3 protein. SPD2 nanoparticles (50 μM) were incubated with GPC3 protein (MW ≈ 61.6 kDa) at a GPC3 protein-to-SPD2 nanoparticles molar ratio of 1:1,000 for 24 h. Experiments were independently repeated three times, with consistent results observed across replicates.

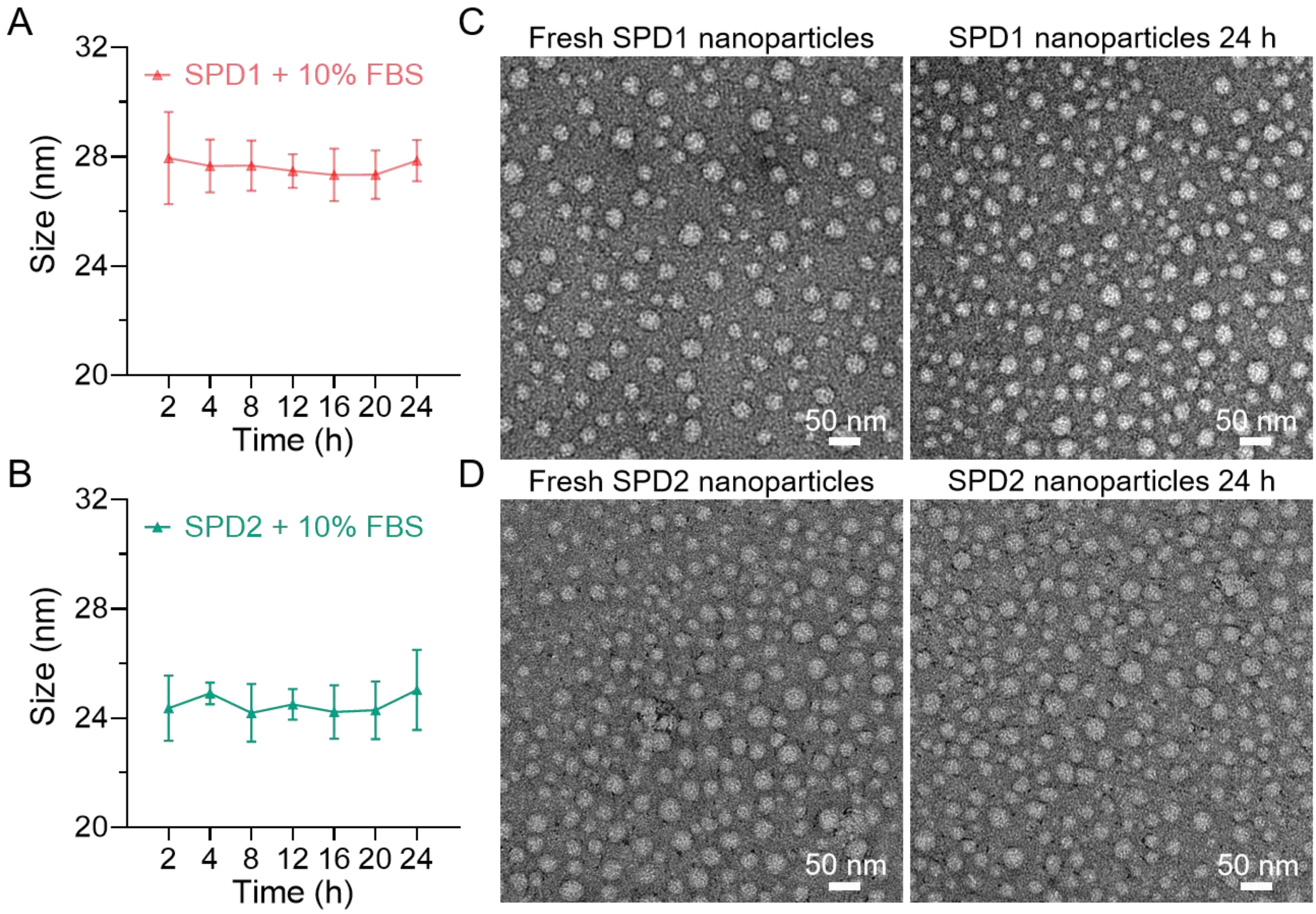

Fig. S17. Stability of SPD1 nanoparticles and SPD2 nanoparticles. (A, B) Particle size of SPD1 nanoparticles (A) and SPD2 nanoparticles (B) measured by DLS after incubation in PBS (pH 7.4) containing 10% fetal bovine serum (FBS) at 37°C. The concentration of SPD1 and SPD2 nanoparticles used in this experiment was 50 µM. Data are presented as mean ± SD from three independent experiments (n = 3). (C) Representative TEM images showing the morphology of freshly prepared SPD1 nanoparticles (50 µM) and SPD1 nanoparticles (50 µM) after storage at room temperature for 24 h. (D) Representative TEM images showing the morphology of freshly prepared SPD2 nanoparticles (50 µM) and SPD2 nanoparticles (50 µM) after storage at room temperature for 24 h.

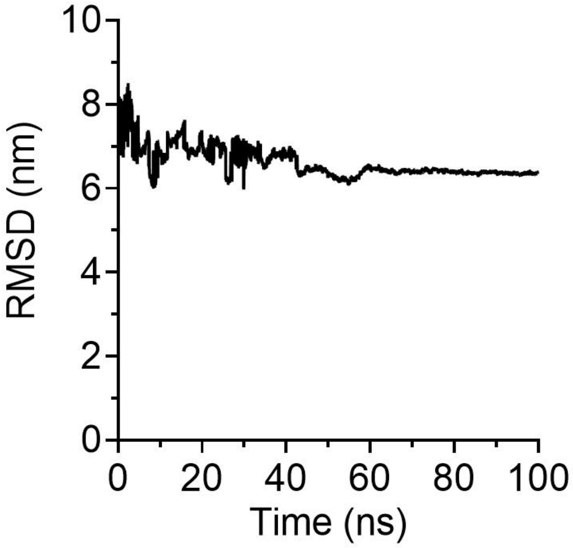

Fig. S18. Root mean square deviation (RMSD) of SPD1 molecules during simulation over time.

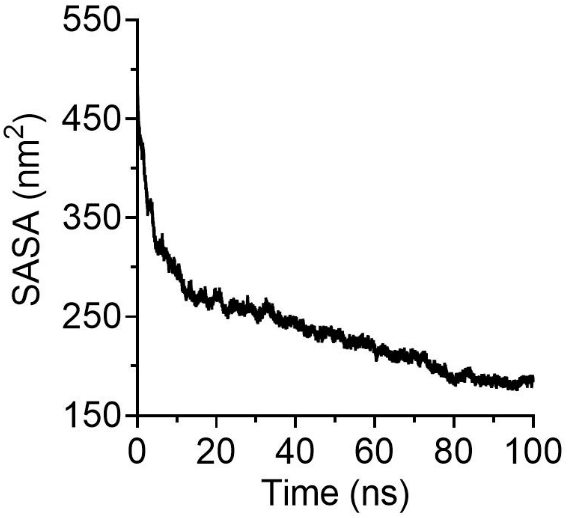

Fig. S19. Solvent accessible surface area (SASA) of SPD1 molecules during simulation over time.

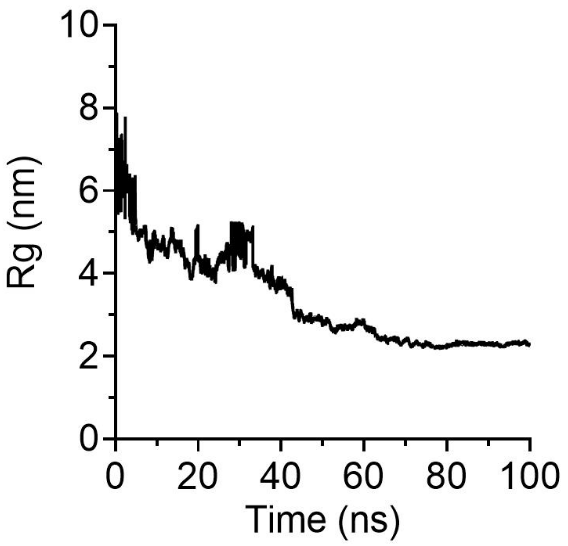

Fig. S20. Radius of gyration (Rg) of SPD1 molecules during simulation over time.

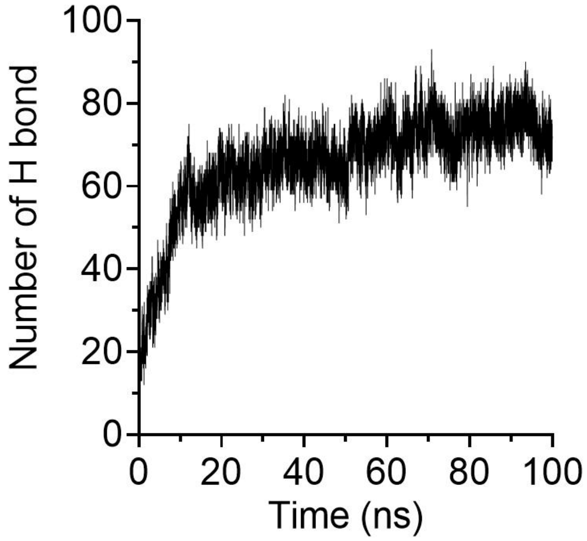

Fig. S21. Number of hydrogen bonds of SPD1 molecules during simulation over time.

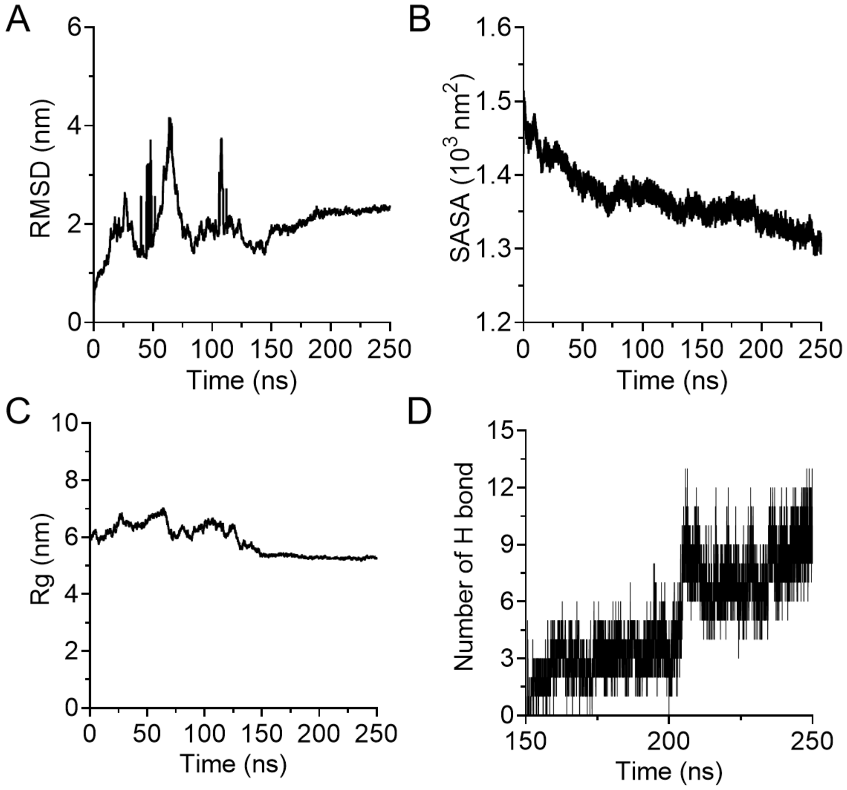

Fig. S22. Molecular dynamics simulation of the GPC3-SPD1 complex. (A) Root mean square deviation (RMSD) of the GPC3-SPD1 complex over a 250-ns simulation, showing strong fluctuations during 0-150 ns, a gradual increase and plateau between 150-190 ns, and stabilization with an average RMSD of 2.28 nm during 200-250 ns. (B) Radius of gyration (Rg) of the GPC3-SPD1 complex, displaying pronounced fluctuations in the early stage (0-150 ns) and stabilization after 190 ns, with an average Rg of 5.26 nm during 200-250 ns. (C) Solvent-accessible surface area (SASA) of the GPC3-SPD1 complex, which decreased progressively during the simulation and reached a stable state with an average value of 1324.69 nm^2 between 200 and 250 ns. (D) Hydrogen bond number between GPC3 and SPD1, remaining relatively stable from 150 to 204 ns and showing a marked increase thereafter.

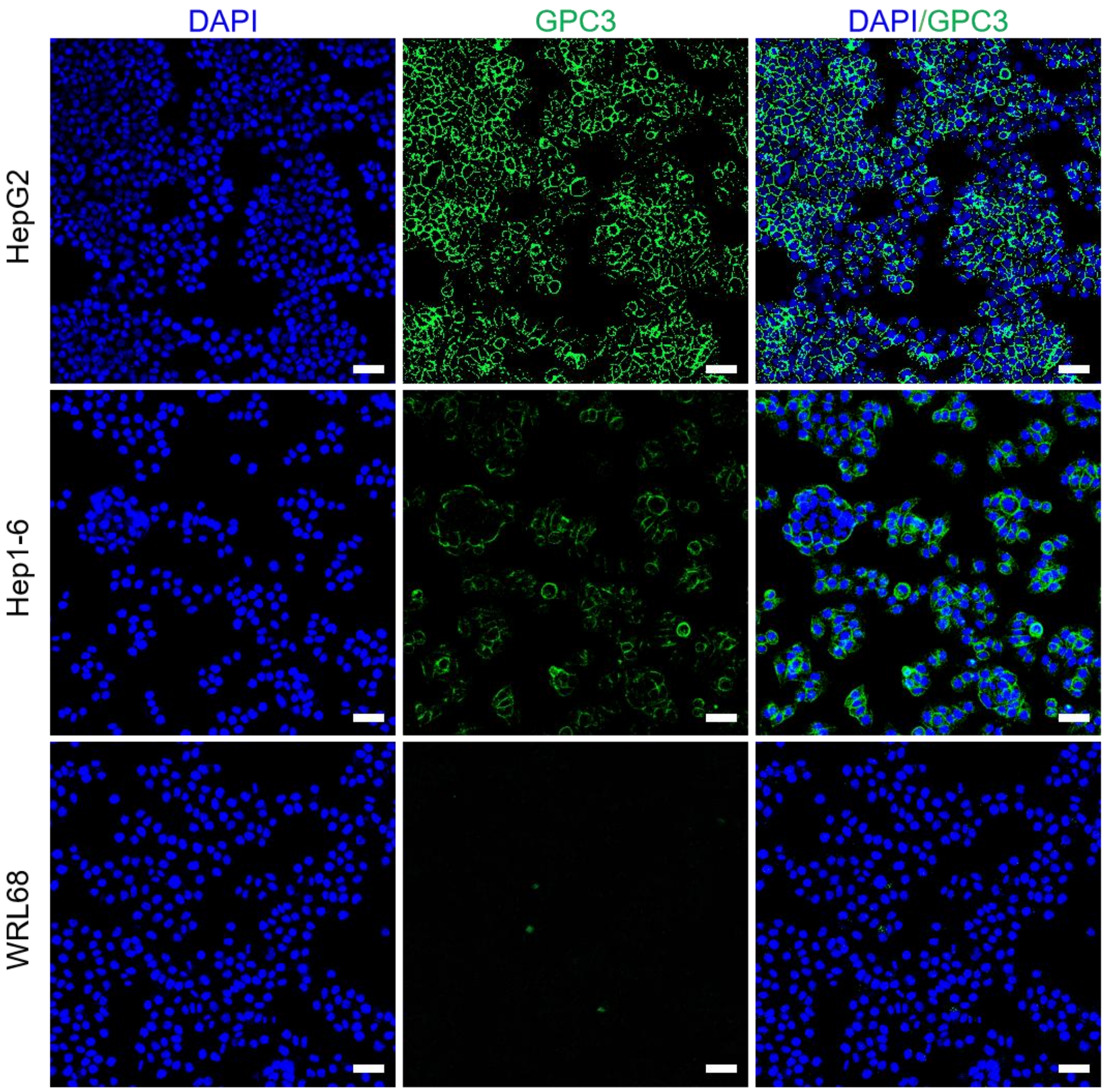

Fig. S23. Immunofluorescence analysis of GPC3 expression in hepatic cell lines. Representative immunofluorescence images showing GPC3 expression (green) in HepG2, Hep1-6, and WRL68 cells. Nuclei were counterstained with DAPI (blue). Images are representative of three independent experiments. Scale bar, 50 μm.

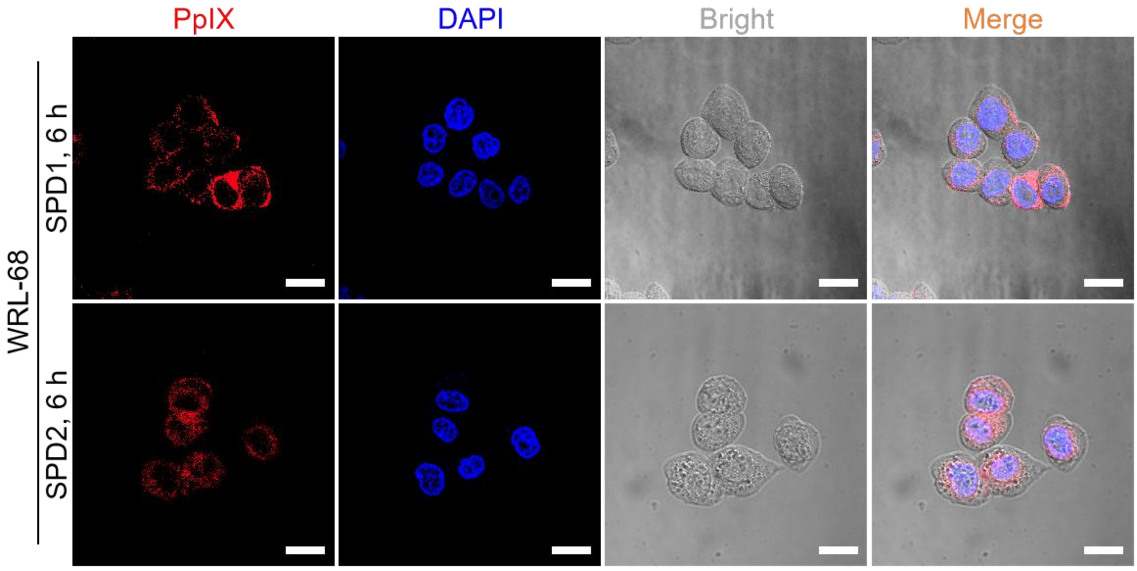

Fig. S24. Intracellular distribution of SPD1 and SPD2 nanoparticles in WRL-68 cells. Confocal laser scanning microscopy (CLSM) images of WRL-68 cells incubated with SPD1 or SPD2 nanoparticles (50 μM; red) at 37°C for 6 h. Representative images from three independent experiments are shown. Scale bar, 20 μm.

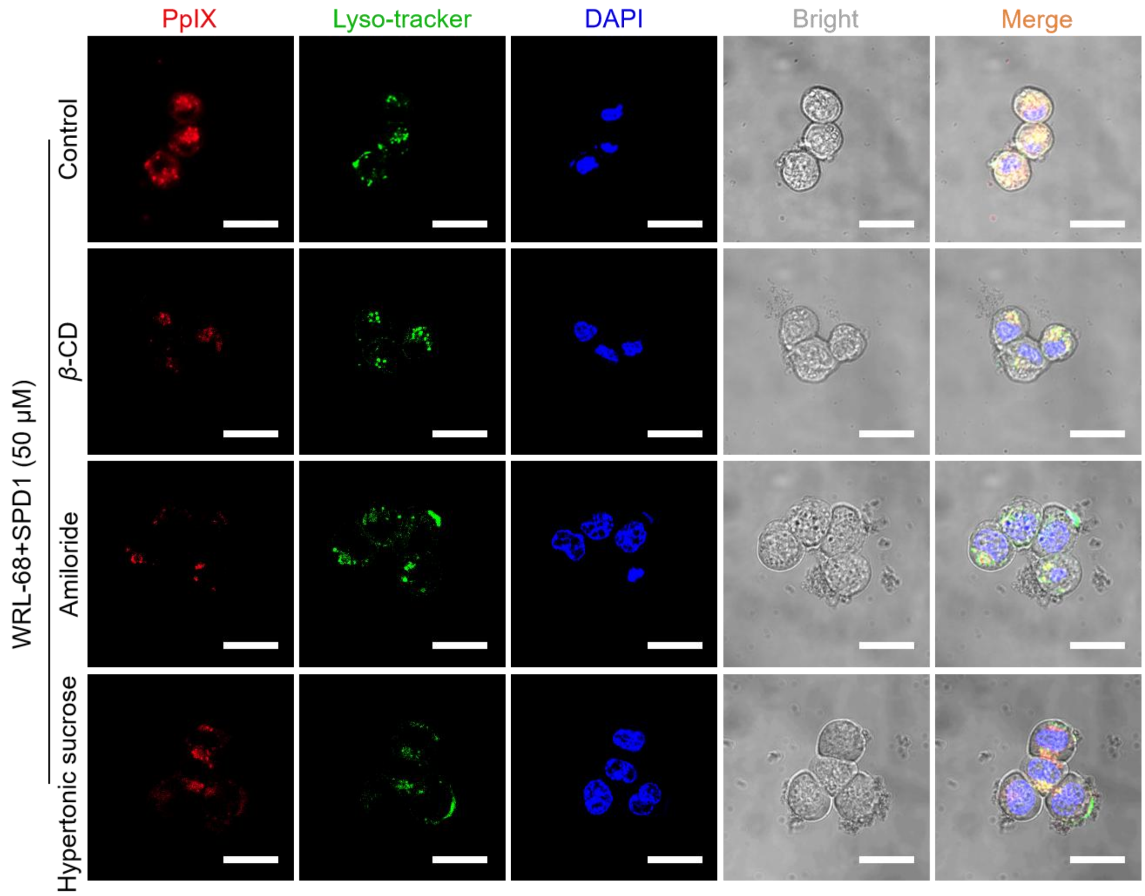

Fig. S25. Inhibition SPD1 nanoparticles uptake by endocytic pathway blockers. CLSM images of WRL-68 cells incubated with SPD1 nanoparticles (50 μM) for 6 h at 37°C in the absence (Control) or presence of β-cyclodextrin (β-CD; 5 mM), amiloride (2 mM), or hypertonic sucrose (450 mM). Representative images from three independent experiments are shown. Scale bar, 20 μm.

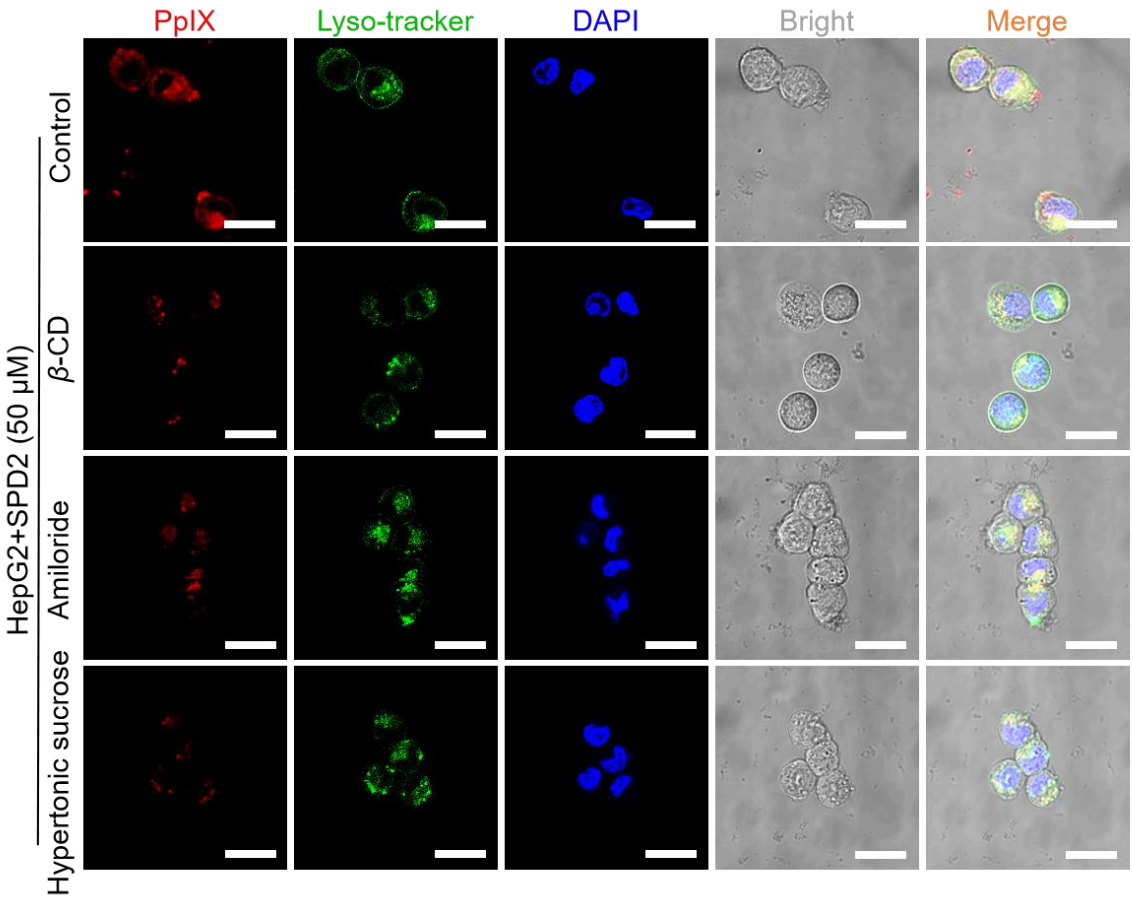

Fig. S26. Inhibition SPD2 nanoparticles uptake by endocytic pathway blockers. CLSM images of WRL-68 cells incubated with SPD2 nanoparticles (50 μM) for 6 h at 37°C in the absence (Control) or presence of β-cyclodextrin (β-CD; 5 mM), amiloride (2 mM), or hypertonic sucrose (450 mM). Representative images from three independent experiments are shown. Scale bar, 20 μm.

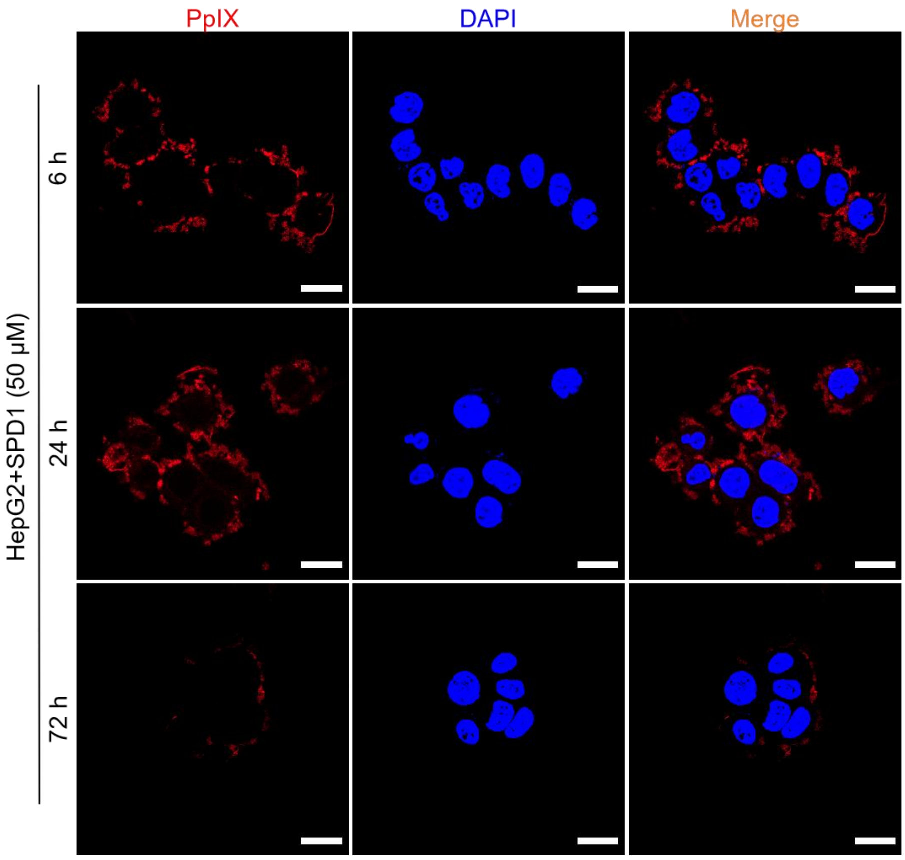

Fig. S27. Sustained membrane retention of SPD1 nanoparticles on HepG2 cells. CLSM images of HepG2 cells incubated with SPD1 nanoparticles (50 μM; red) at 37°C for 6, 24, and 72 h. Representative images from three independent experiments are shown. Scale bar, 20 μm.

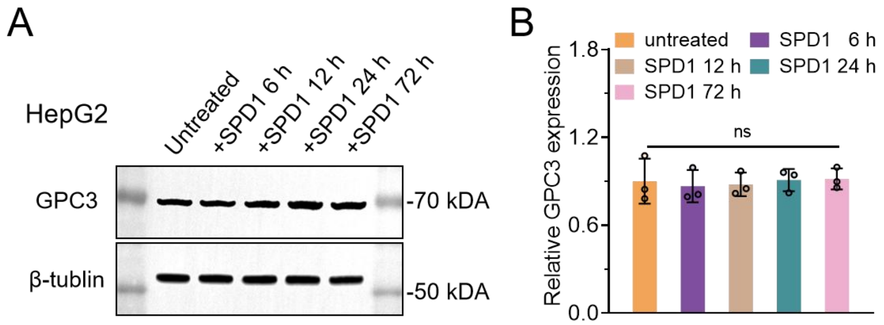

Fig. S28. Effect of SPD1 on membrane-associated GPC3 in HepG2 cells. (A) Representative western blotting analysis of GPC3 expression in untreated HepG2 cells and in cells treated with SPD1 (50 μM) for 6, 12, 24, or 72 h. (B) Quantification of relative GPC3 in untreated and SPD1-treated cells. Data are presented as mean ± SD ($n = 3$ independent experiments). Statistical analysis indicates no significant difference between untreated and SPD1-treated groups (ns, one-way ANOVA).

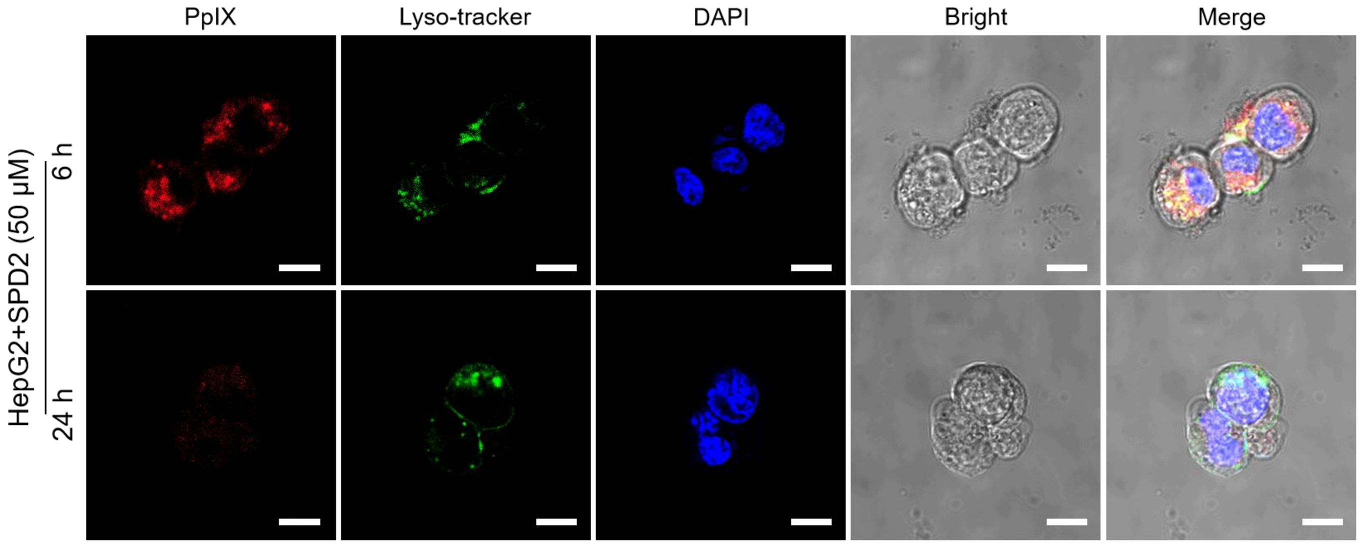

Fig. S29. Cellular distribution of SPD2 nanoparticles in HepG2 cells. CLSM images of HepG2 cells incubated with SPD2 nanoparticles (50 μM) for 6 and 24 h. Most internalized SPD2 nanoparticles co-localized with lysosomes and underwent lysosomal degradation after 24 h. Images are representative of three independent experiments. Scale bars, 10 μm.

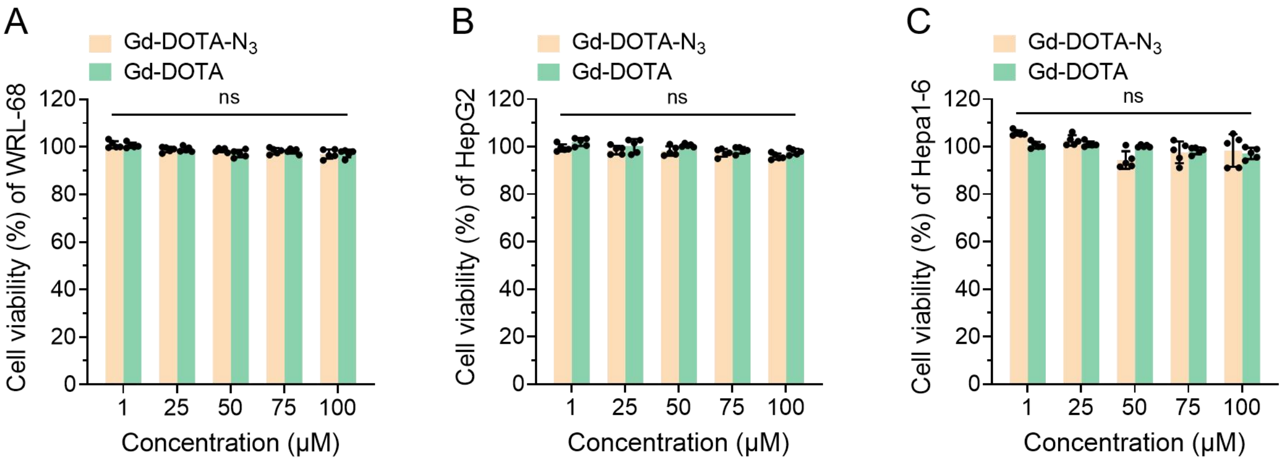

Fig. S30. In vitro analysis of cell viability. Cell viability of WRL-68 (A), HepG2 (B), and Hepa1-6 (C) cells after 24 h exposure to various concentrations (1-100 μM) of Gd-DOTA-N_3 or Gd-DOTA. Cell viability was quantified using the CCK-8 assay. Data are presented as mean ± SD (n = 5). Statistical significance was performed using two-way ANOVA followed by Tukey's post hoc test. $P < 0.05$ was considered statistically significant; ns, not significant.

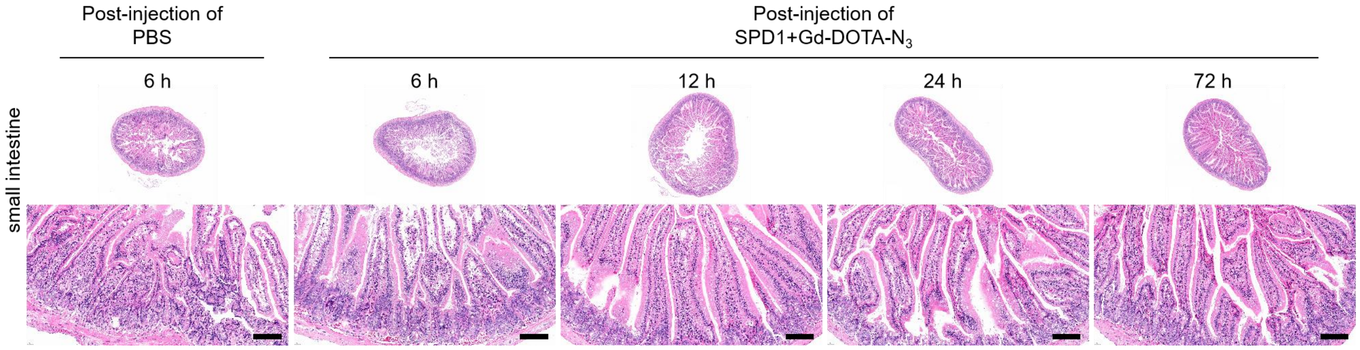

Fig. S31. Assessment of the intestinal effects of SPD1+Gd-DOTA-N_3. Histological sections of the small intestine from C57BL/6 mice, including 6 h following a single i.v. injection of PBS (200 μL; Control), and 6, 12, 24 and 72 h following sequential i.v. injection of SPD1 (10 mg/kg) and Gd-DOTA-N_3 (0.1 mmol/kg). Representative images from 3 biologically independent mice per group and time point. Scale bar, 100 μm.

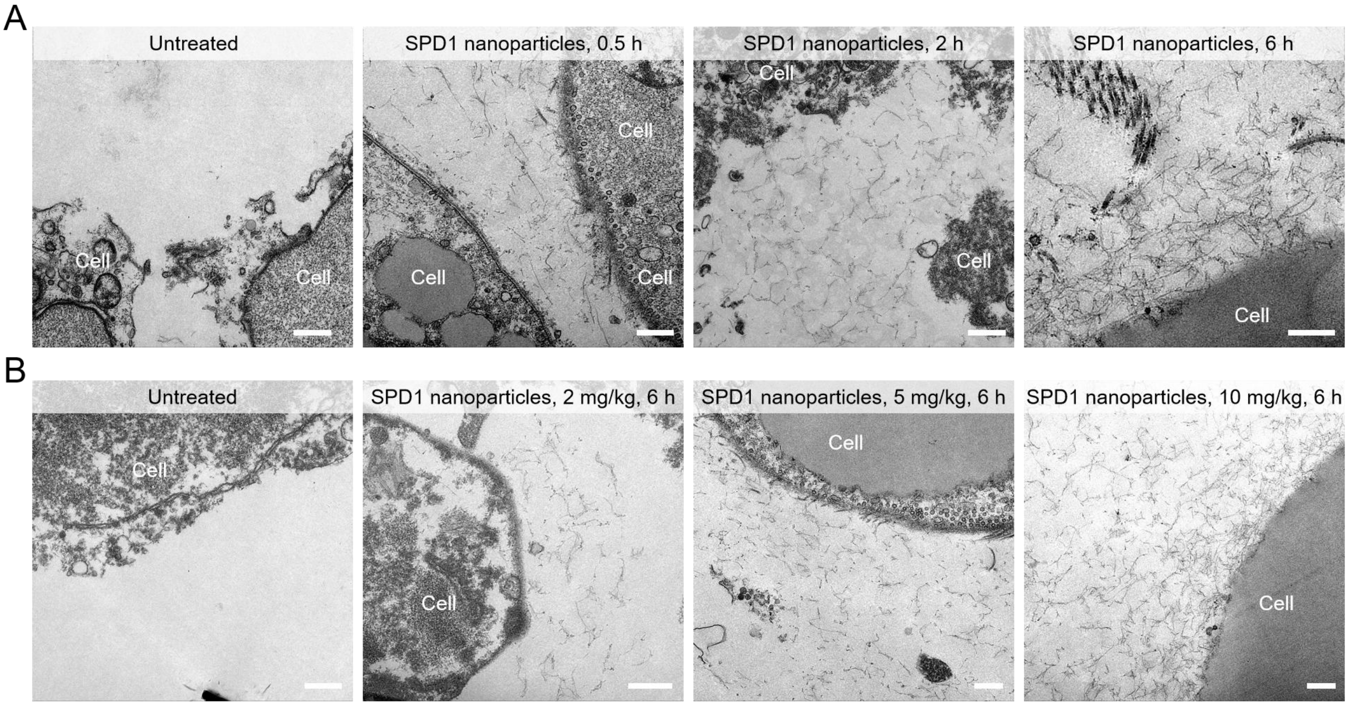

Fig. S32. Time- and dose-dependent in situ fibrillar transformation of SPD1 nanoparticles in tumor tissues of Hepa1-6 tumor bearing mice. TEM images showing the spatial distribution and fibrillar transformation of SPD1 nanoparticles on tumor cell membranes. (A) Time-course analysis following i.v. administration of SPD1 nanoparticles (10 mg/kg), with images collected at 0.5, 2 and 6 h post-injection; untreated tumor tissues served as controls. SPD1 nanoparticles transitioned into dense nanofibrillar networks along the plasma membrane over time. (B) Dose-dependent fibrillar assembly in tumor tissues 6 h after injection of SPD1 nanoparticles at 2, 5 and 10 mg/kg, alongside untreated controls. Increasing doses led to more extensive membrane-associated fibrillar scaffolds. Scale bars, 500 nm. All experiments were independently repeated three times with consistent results.

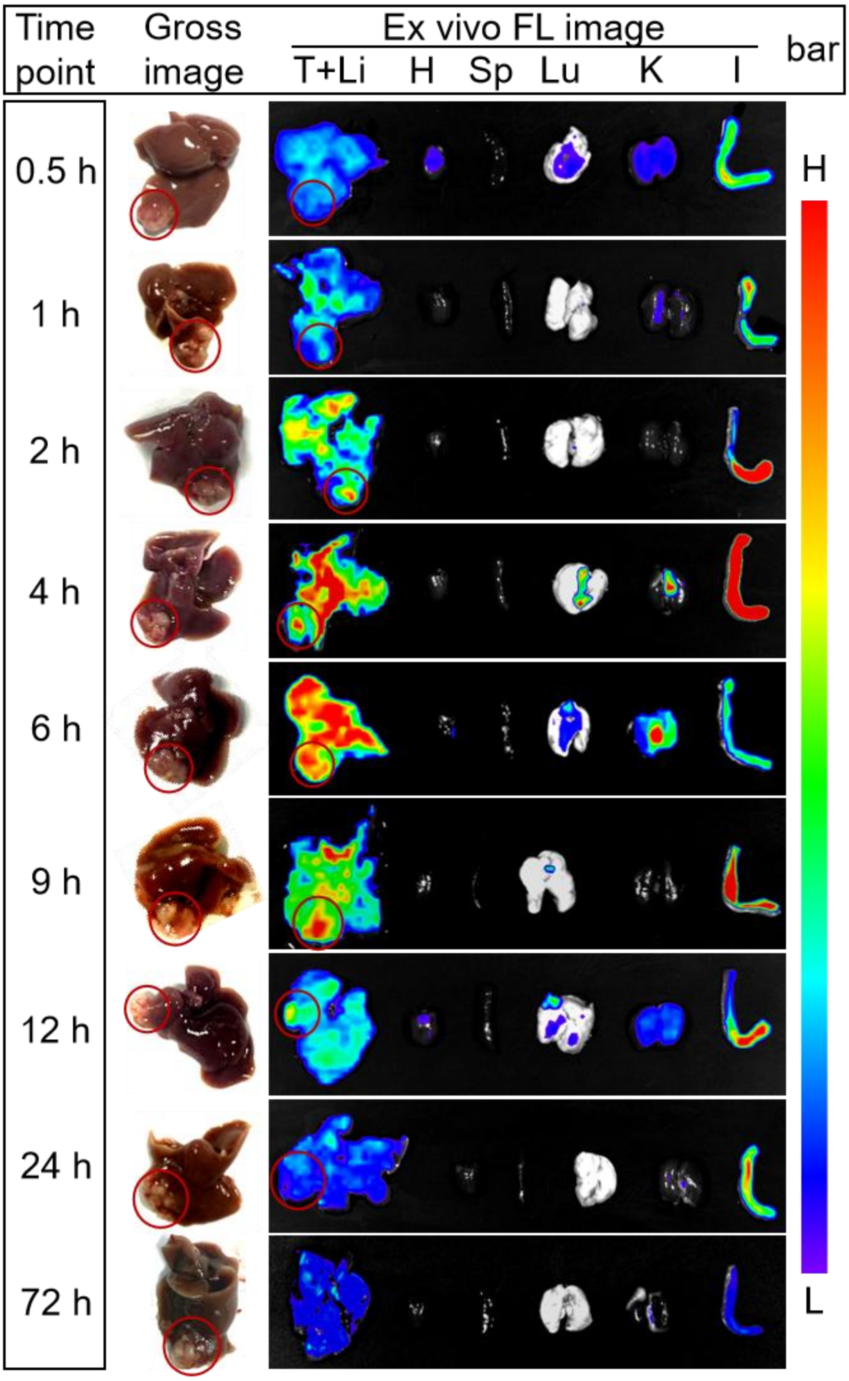

Fig. S33. In vivo biodistribution of SPD2 in an orthotopic liver tumor model. Ex vivo fluorescence (FL) images of tumors (T; demarcted by red dashed circles) and major organs, including the heart (H), liver (Li), spleen (Sp), lungs (Lu), kidneys (K), and intestine (I), collected at 0.5, 1, 2, 4, 6, 9, 12, 24 and 72 h after i.v. injection of SPD2 nanoparticles (10 mg/kg) in C57BL/6 mice bearing orthotopic Hepa1-6 liver tumors. Representative images from 3 biologically independent mice per time point are shown.

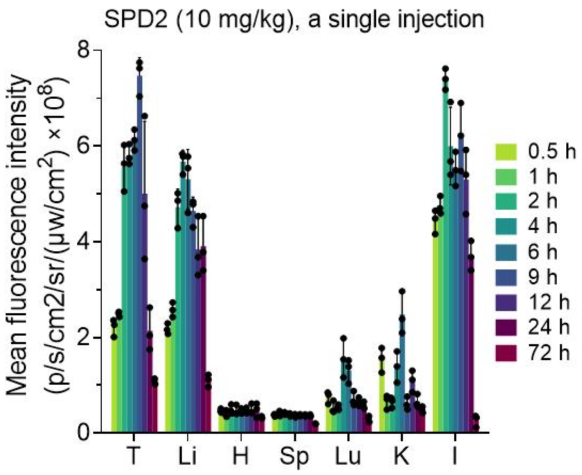

Fig. S34. Quantitative analysis of SPD2 biodistribution in an orthotopic liver tumor model. Fluorescence intensity in tumor (T) and major organs, including the heart (H), liver (Li), spleen (Sp), lungs (Lu), kidneys (K), and intestine (I), collected at 0.5, 1, 2, 4, 6, 9, 12, 24 and 72 h after i.v. injection of SPD2 nanoparticles (10 mg/kg) in C57BL/6 mice bearing orthotopic Hepa1-6 liver tumors. Data are presented as mean ± SD (n = 3 biologically independent mice per time point).

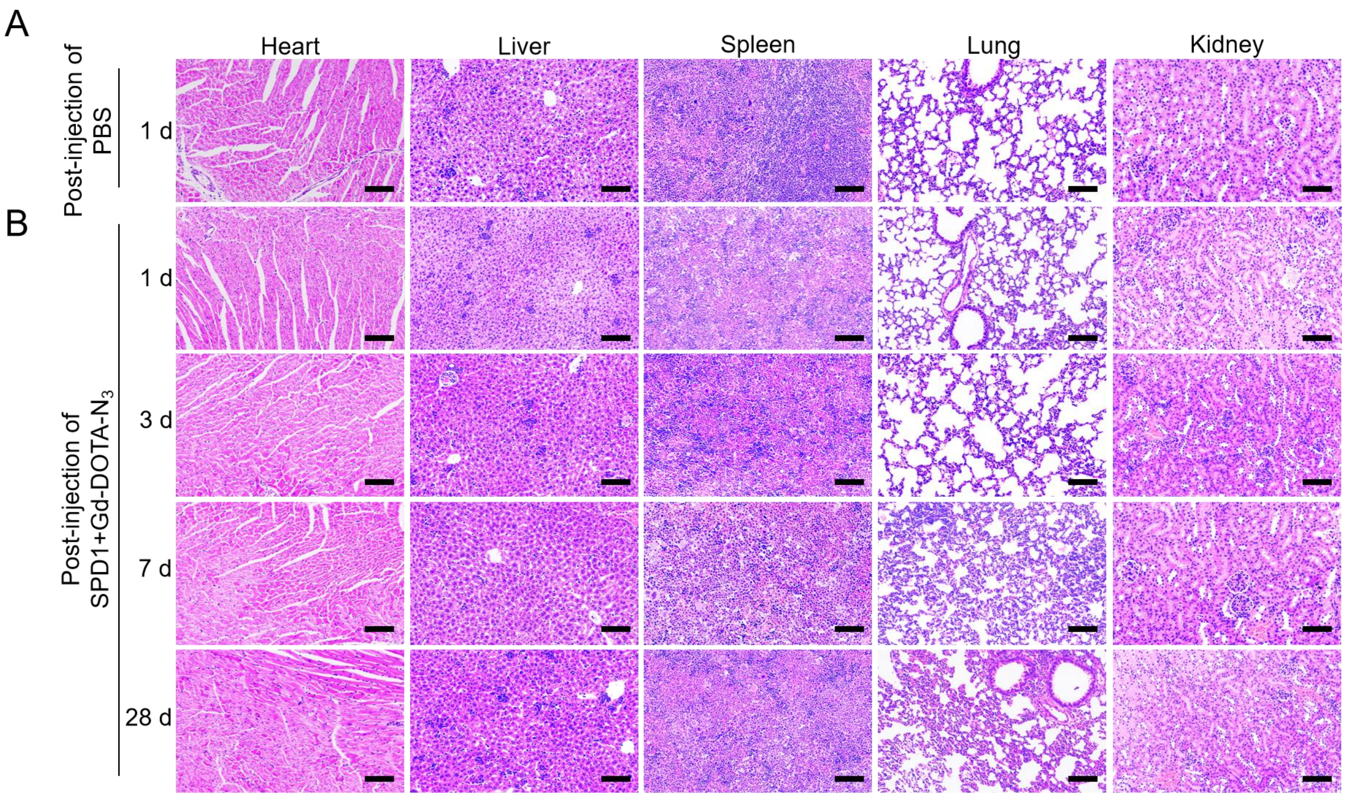

Fig. S35. In vivo biocompatibility of SPD1 assessed by H&E staining. Histological sections of heart, liver, spleen, lungs, and kidneys from C57BL/6 mice: (A) Day 1 following a single i.v. injection of PBS (200 µL; Control); (B) Days 1, 3, 7 and 28 following sequential i.v. injection of SPD1 (10 mg/kg) and Gd-DOTA-N_3 (0.1 mmol/kg). Representative images from 3 biologically independent mice per group and time point. Scale bar, 100 µm.

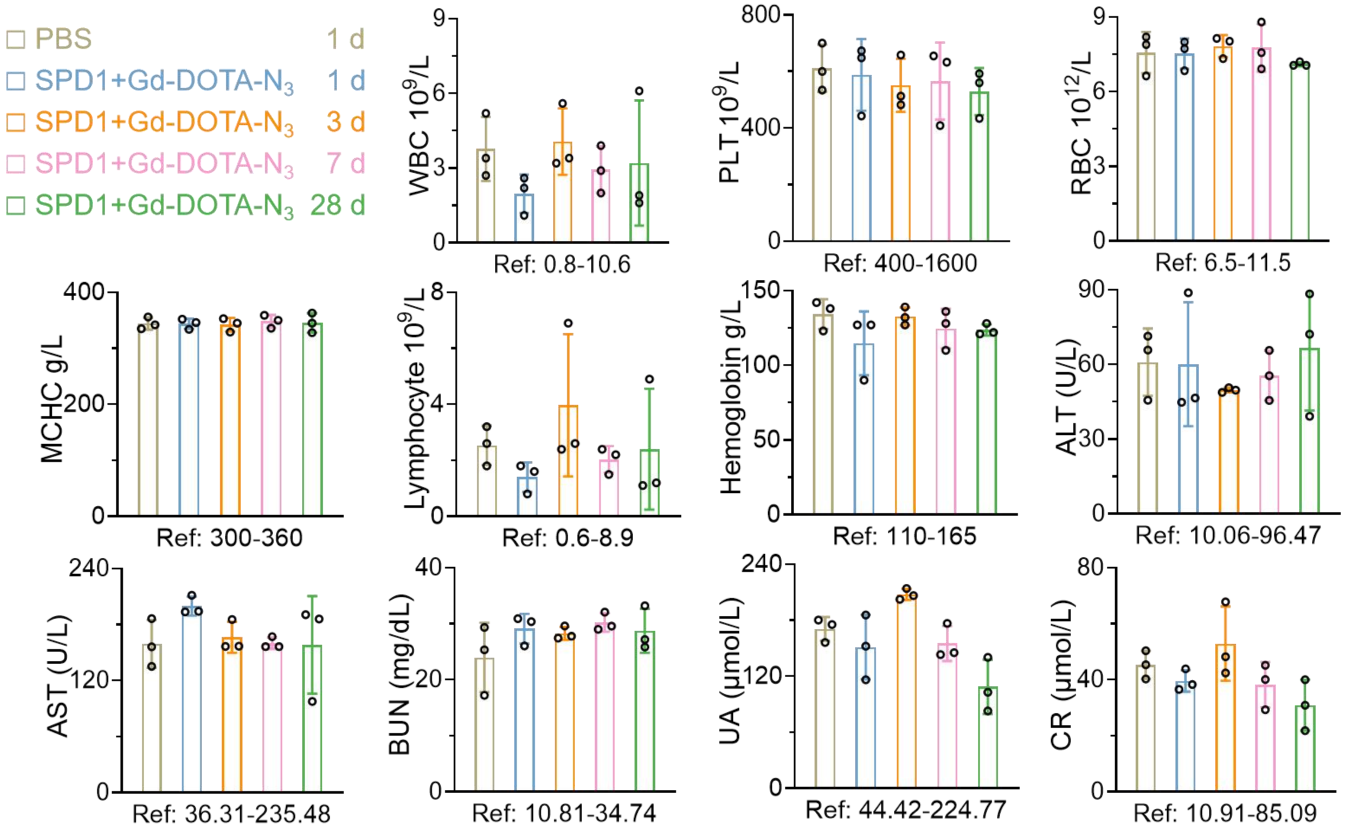

Fig. S36. Hematological and biochemical safety profiling of SPD1 nanoparticles in healthy mice. Hematological parameters—including white blood cells (WBC), platelets (PLT), red blood cells (RBC), mean corpuscular hemoglobin concentration (MCHC), lymphocytes, and hemoglobin—and serum biochemical markers—including alanine aminotransferase (ALT), aspartate transaminase (AST), blood urea nitrogen (BUN), uric acid (UA), and creatinine (CR)—were assessed in C57BL/6 mice on Day 1 following a single i.v. injection of PBS (200 μL; control), and on Days 1, 3, 7, and 28 following sequential i.v. administration of SPD1 (10 mg/kg) and Gd-DOTA-N_3 (0.1 mmol/kg). Data are presented as mean ± SD (*n* = 3 biologically independent mice per group and time point).

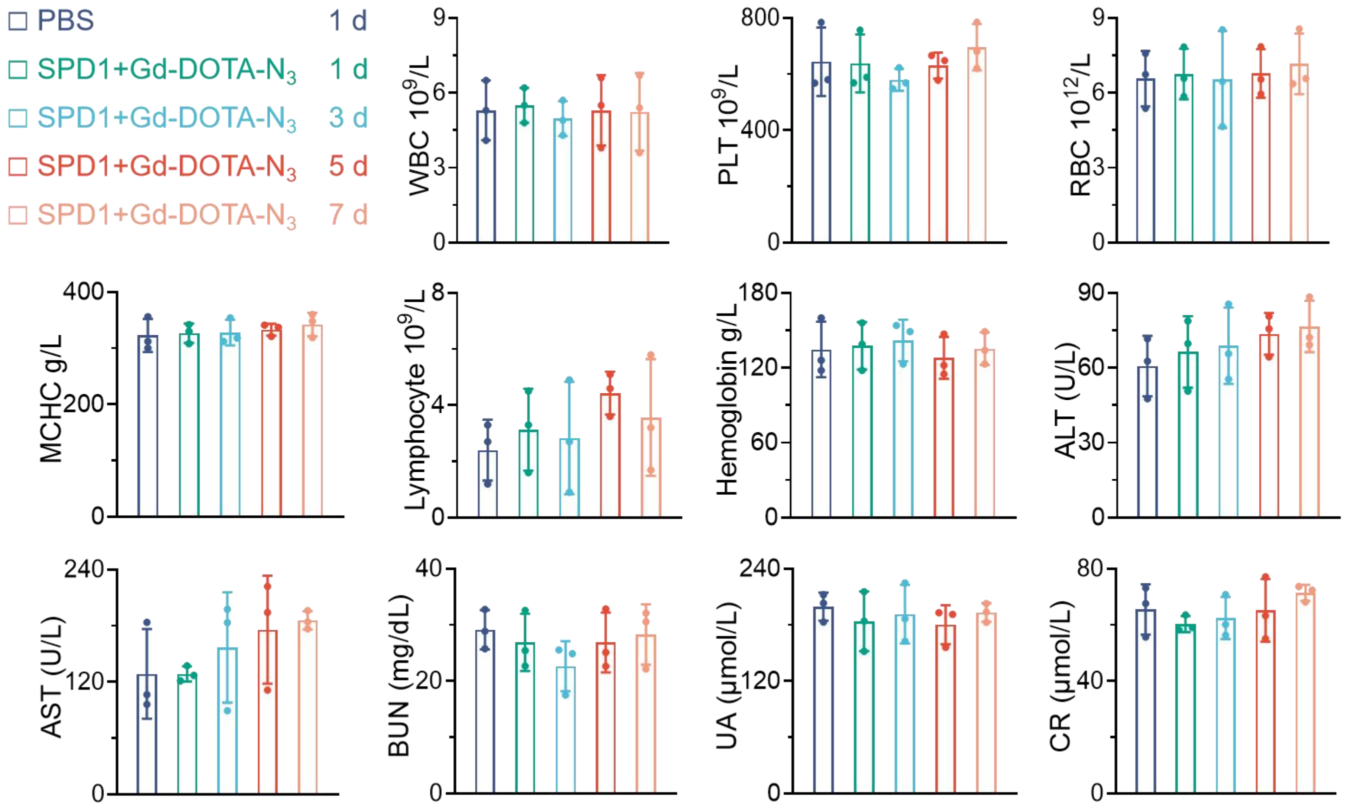

Fig. S37. Hematological and biochemical safety profiling of SPD1 nanoparticles in C57BL/6 mice bearing Hepa1-6 tumor. Hematological parameters—including WBC, PLT, RBC, MCHC, lymphocytes, and hemoglobin—and serum biochemical markers—including ALT, AST, BUN, UA and CR—were assessed in C57BL/6 mice bearing Hepa1-6 tumor on Day 1 following a single i.v. injection of PBS (200 μL; control), and on Days 1, 3, 5, and 7 following sequential i.v. administration of SPD1 (10 mg/kg) and Gd-DOTA-N_3 (0.1 mmol/kg). Data are presented as mean ± SD (*n* = 3 biologically independent mice per group and time point).

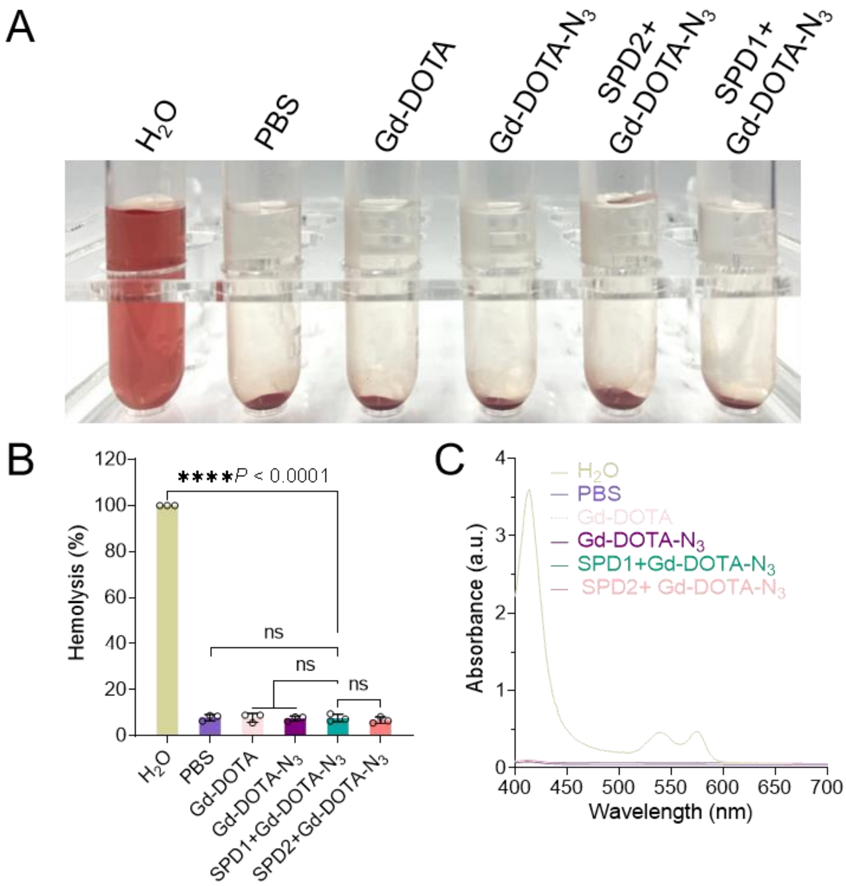

Fig. S38. Hemocompatibility assessment. (A) Photographs, (B) UV-vis absorption spectra, and (C) hemolysis percentage of RBCs after 6 h incubation at 37°C with PBS (negative control), Gd-DOTA (0.5 mg/mL), Gd-DOTA-N_3 (0.5 mg/mL), SPD1 (0.5 mg/mL)+Gd-DOTA-N_3 (0.5 mg/mL), SPD2 (0.5 mg/mL)+Gd-DOTA-N_3 (0.5 mg/mL), and deionized water (positive control). Data are presented as mean ± SD (n = 3 biologically independent experiments). Statistical significance was determined by one-way ANOVA followed by Tukey's multiple comparison test. ns, not significant.

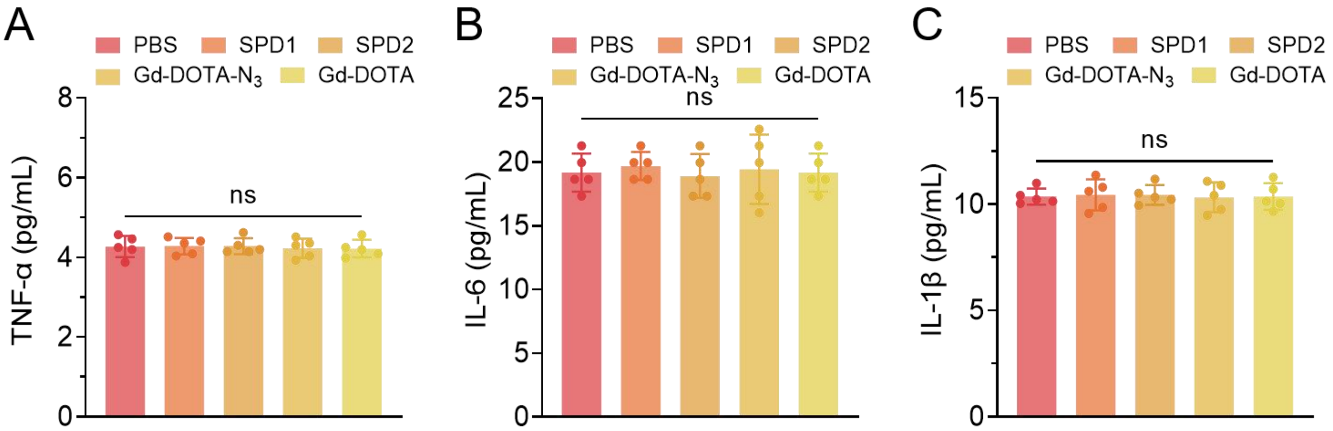

Fig. S39. Pro-inflammatory cytokines, including serum TNF-α (A), IL-6 (B), and IL-1β (C) quantified 6 h after a single i.v. administration of the five indicated treatments. Data are presented as mean ± SD ($n = 5$ independent experiments). Statistical analysis was performed using ordinary one-way ANOVA followed by Tukey's multiple-comparisons test; ns, not significant.

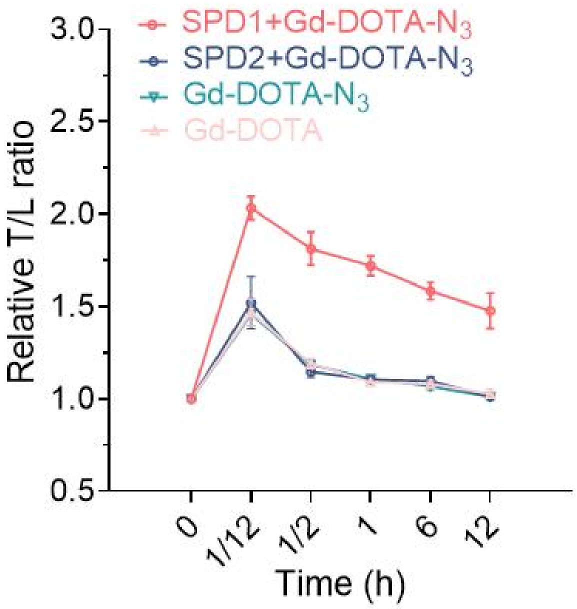

Fig. S40. Longitudinal tumor-to-liver (T/L) T_1 signal intensity ratios in mice following administration of SPD1+Gd-DOTA-N_3, SPD2+Gd-DOTA-N_3, free Gd-DOTA-N_3, or Gd-DOTA. Mice (n = 3 per group) were subjected to T_1-weighted MRI at designated time points post-injection. SPD1+Gd-DOTA-N_3 exhibited sustained elevation in T/L signal ratio compared to other groups, indicating enhanced tumor accumulation and retention. Data represent mean ± SD from 3 biologically independent mice.

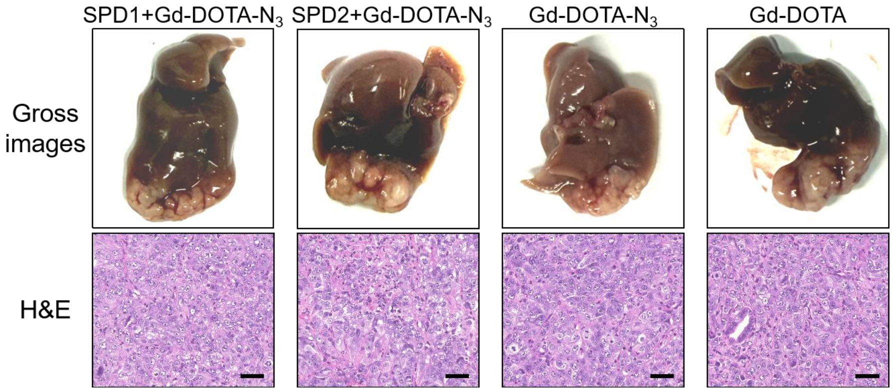

Fig. S41. Gross morphology and histopathological validation of orthotopic Hepa1-6 tumor-bearing mice. Representative macroscopic images (top) and H&E staining (bottom) of liver tissues from mice in four experimental groups. macroscopic images reveal tumor nodules predominantly localized in the liver lobes. HE staining shows dense and disorganized tumor cell architecture with enlarged nuclei and increased nuclear-to-cytoplasmic ratios. Scale bars, 40 µm.

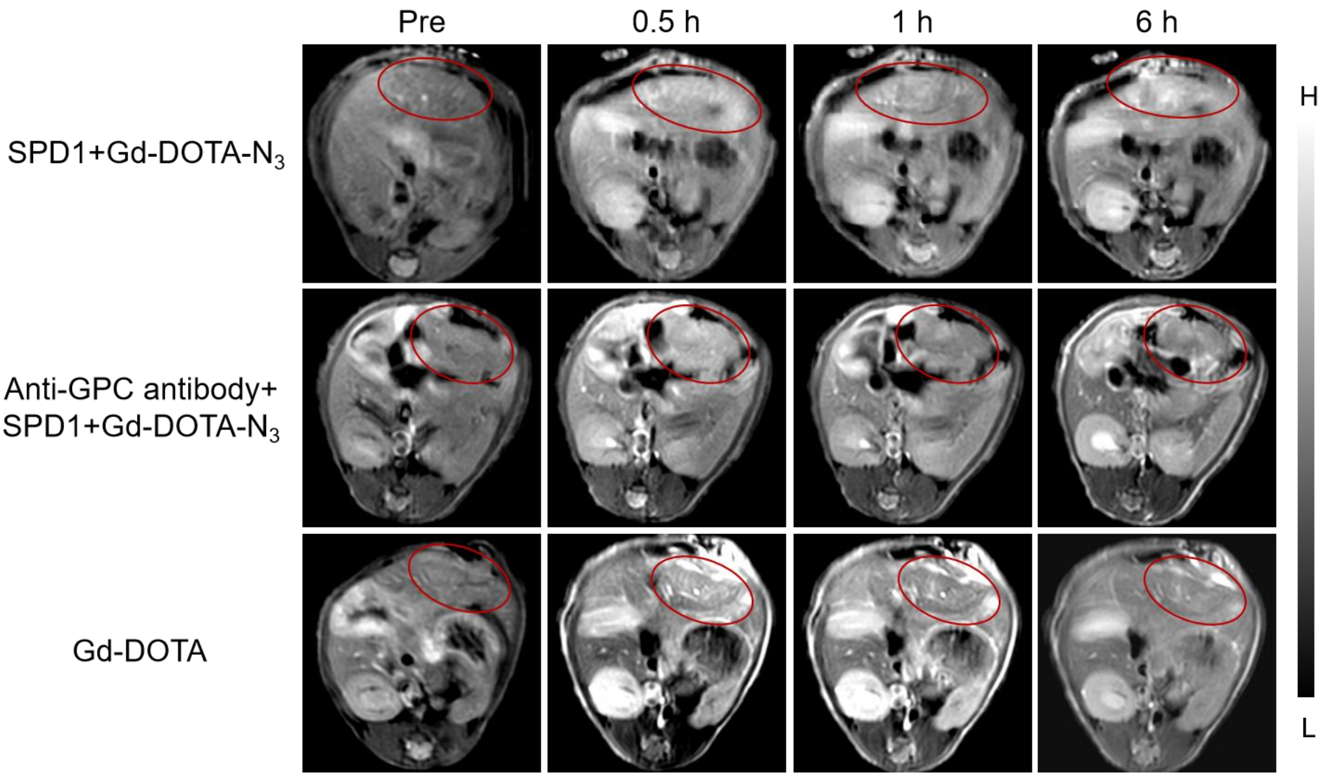

Fig. S42. MRI detection of liver tumors with or without GPC3 blocking in orthotopic Hepa1-6 tumor-bearing C57BL/6 mice. T_1-weighted MR images were acquired at indicated time points following i.v. injection of Gd-DOTA-N_3 (0.1 mmol/kg) and SPD1 (10 mg/kg, 6 h later), with or without preadministration of anti-GPC3 antibody (0.6 mg/kg) to block ligand-receptor interactions. Red circles mark tumor regions. Images shown are representative of three biologically independent mice per group.

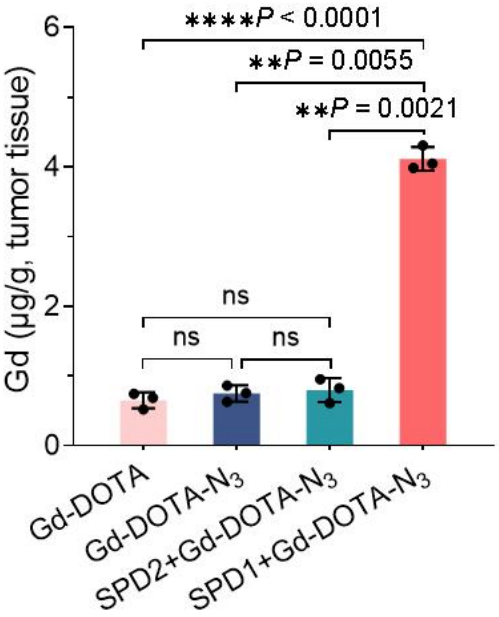

Fig. S43. Quantification of Gd accumulation in orthotopic tumors 1 h post-Gd injection across four treatment protocols. Mice were treated with SPD1+Gd-DOTA-N_3, SPD2+Gd-DOTA-N_3, Gd-DOTA-N_3 alone, or Gd-DOTA alone. Gd content in tumor tissues was measured by ICP-MS. SPD1+Gd-DOTA-N_3 exhibited significantly enhanced tumor accumulation compared to other groups. Data are presented as mean ± SD (n = 3 mice). Statistical significance was determined by one-way ANOVA with Turkey's multiple comparisons test.

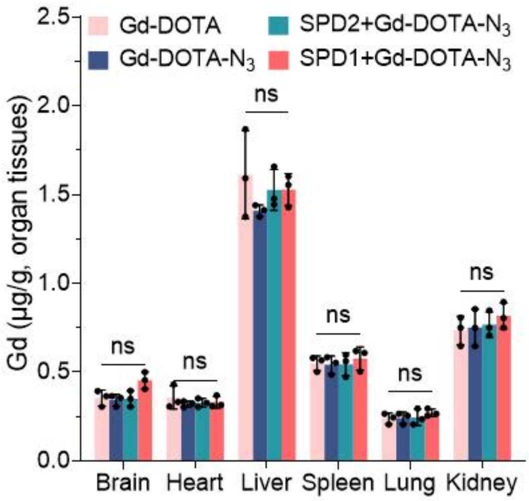

Fig. S44. Bio-distribution of Gd in major organs. Gd content in major organs of orthotopic Hepa1-6 tumor-bearing C57BL/6 mice quantified by ICP-MS at 1 h following: (i) sequential i.v. administration of SPD1 (10 mg/kg) and Gd-DOTA-N_3 (0.1 mmol/kg, 6 h later), (ii) SPD2 (10 mg/kg) and Gd-DOTA-N_3 (0.1 mmol/kg, 6 h later), (iii) Gd-DOTA-N_3 alone (0.1 mmol/kg, i.v.), or (iv) Gd-DOTA alone (0.1 mmol/kg, i.v.). Data are presented as mean ± SD (n = 3 biologically independent mice).

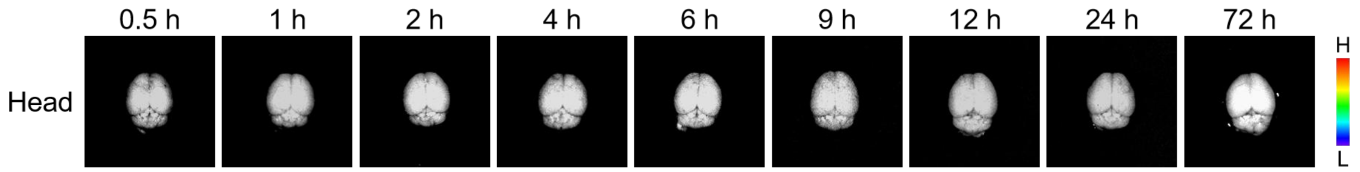

Fig. S45. In vivo head biodistribution of SPD1 in an orthotopic liver tumor model. Representative ex vivo fluorescence images of the head collected at 0.5, 1, 2, 4, 6, 9, 12, 24, and 72 h after i.v. injection of SPD1 nanoparticles (10 mg/kg) in C57BL/6 mice bearing orthotopic Hepa1-6 liver tumors.

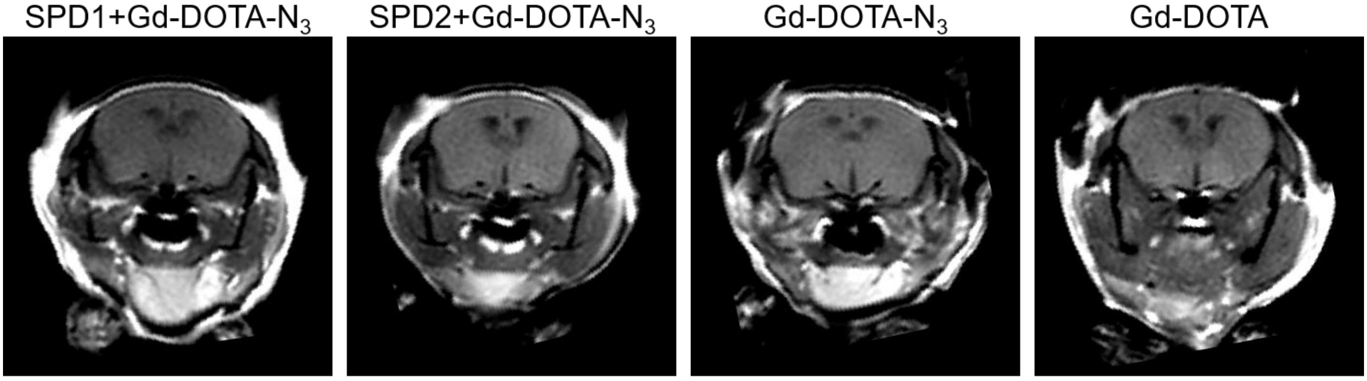

Fig. S46. Long-term cerebral safety assessment by MRI. T_1-weighted MRI was performed on Day 7 after administration of four formulations (SPD1+Gd-DOTA-N_3, SPD2+Gd-DOTA-N_3, Gd-DOTA-N_3 and Gd-DOTA) to assess potential Gd retention in the basal ganglia. Representative coronal images from all groups show comparable signal intensities and no abnormal signal foci, indicating the absence of detectable long-term Gd deposition under any treatment condition.

Table S1. Key pharmacokinetics parameters of SPD1 and SPD2

Parameters	SPD1	SPD2	*P* value
C_{max} (μg/mL)	71.76 ± 5.54	68.65 ± 8.15	0.614
AUC_{0-24} (μg·h/mL)	164.43 ± 3.96	79.96 ± 4.06	< 0.0001
$T_{1/2z}$ (h)	6.29 ± 0.96	3.58 ± 1.00	0.028
CL (mL/h/kg)	1.13 ± 0.02	11.09 ± 1.19	< 0.0001
MRT (h)	7.13 ± 0.27	2.46 ± 0.13	< 0.0001

Note: *P* value was obtained by the t test; C_{max}, maximum plasma concentration; AUC_{0-24}, area under the concentration-time curve from 0 to 24 h; $T_{1/2z}$, terminal elimination half-life; CL, systemic clearance; MRT, mean residence time.

Table S2. Key pharmacokinetics parameters of Gd in different groups

Group	SPD1+Gd-DOTA-N_3	SPD2+Gd-DOTA-N_3	Gd-DOTA-N_3	Gd-DOTA	*P* Value
C_{max} (μg/mL)	123.20 ± 12.10	132.00 ± 10.86	130.63 ± 4.78	115.93 ± 16.66	0.4228
AUC_{0-24} (μg·h/mL)	115.85 ± 6.32	124.10 ± 2.72	120.84 ± 3.74	116.53 ± 10.38	0.7404
$T_{1/2z}$ (h)	3.26 ± 0.31	3.30 ± 0.59	3.71 ± 0.87	3.82 ± 1.13	0.3065
CL ((mg)/(μg/mL)/h)	7.09 ± 0.37	6.59 ± 0.17	6.76 ± 0.15	7.06 ± 0.62	0.4475
MRT (h)	2.29 ± 0.14	2.18 ± 0.32	2.52 ± 0.26	2.52 ± 0.40	0.3104

Note: *P* value was obtained by one-way ANOVA with a Tukey's post hoc test. C_{max}, maximum plasma concentration; AUC_{0-24}, area under the concentration-time curve from 0 to 24 h; $T_{1/2z}$, terminal elimination half-life; CL, systemic clearance; MRT, mean residence time.
